# Understanding the Mechanism of Triplet‐Triplet Energy Transfer in the Photocatalytic [2 + 2] Cycloaddition: Insights From Quantum Chemical Modeling

**DOI:** 10.1002/jcc.70155

**Published:** 2025-06-11

**Authors:** Eunji Lee, Hyejin Moon, Jiyong Park, Mu‐Hyun Baik

**Affiliations:** ^1^ Department of Chemistry Korea Advanced Institute of Science and Technology (KAIST) Daejeon Republic of Korea; ^2^ Center for Catalytic Hydrocarbon Functionalizations Institute for Basic Science (IBS) Daejeon Republic of Korea

**Keywords:** chiral induction, marcus theory, photocatalysis, photocycloaddition, triplet‐triplet energy transfer

## Abstract

We investigate the mechanism of [2 + 2] photocycloaddition reaction of 3‐(but‐3‐enyl)oxyquinolone using a chiral xanthone‐containing triplet sensitizer. Quantum chemical computer models were utilized to examine the substrate‐catalyst encounter complex structures, which were classified into *syn*‐ and *anti‐*adducts. The photoactivation steps of the substrate were analyzed based on the Marcus equation of electron transfer, including intersystem crossings (ISC) and outer sphere triplet‐triplet energy transfer (TTEnT). Our results show that the calculated rates of ISC are comparable for the two adducts, while the rates of TTEnT differ due to the orbital overlap between the donor and acceptor sites. After the TTEnT, a stereospecific cyclization occurs, completing the catalytic cycle. We propose a strategy to improve stereoselectivity by exploiting the intrinsic difference in TTEnT rates between the two encounter complex isomers.

## Graphical abstract

The study explores a photocatalytic reaction to activate an intramolecular [2 + 2] cycloaddition of a 3‐(but‐3‐enyl)oxyquinolone substrate using a chiral xanthone catalyst. Computational analysis of two substrate‐catalyst encounter complexes reveals different rates for energy transfer due to orbital overlap. A strategy is proposed to improve stereoselectivity and enhance substrate scope based on the energy transfer rate differences.

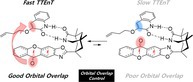

## 1 Introduction

The photoactivated [2 + 2] cycloaddition of enones and alkenes provides a valuable route to cyclobutanes, circumventing the constraints posed by the orbital symmetry‐forbidden nature of thermal [2 + 2] reactions in the ground state [1]. Unfortunately, traditional approaches that rely on high‐powered UV‐light sources typically suffer from low degrees of stereo‐ and regioselectivity and undesirable side reactions, which limit their practical utility. Recently, photocatalytic reactions utilizing triplet photosensitizers have emerged as promising alternatives, offering superior light absorption efficiencies and product yields by delivering the precise amount of energy needed for activation to the desired substrate. In addition, organic triplet sensitizers incorporating stereospecific hydrogen bonding scaffolds have been developed, achieving remarkable enantioselectivity in [2 + 2] photocycloadditions [2, 3, 4, 5, 6, 7, 8, 9]. Inorganic triplet sensitizers, such as chiral Ir(III) photocatalysts bearing hydrogen bonding scaffolds, have also been demonstrated to yield high enantioselectivities [10, 11, 12].

The mechanistic understanding of the [2 + 2] photocycloadditions of enones and alkenes was pioneered by Corey and De Mayo [13, 14, 15]. This mechanism involves the photoexcitation of the enone substrate, followed by intersystem crossing (ISC) to yield a triplet biradical species, which then undergoes a stepwise [2 + 2] cycloaddition with the ground state alkene substrate [16]. In photocatalytic processes, the photoexcitation and rapid ISC within the photocatalyst allow the sensitizer to access the triplet excited state manifold. Subsequently, a triplet‐triplet energy transfer (TTEnT) occurs, which exchanges the triplet character of the sensitizer with the singlet character of the enone substrate via a two‐electron exchange mechanism known as the Dexter process [17, 18, 19, 20]. After the TTEnT, the substrate accesses its triplet manifold and undergoes the bond‐forming events in a stepwise manner [21, 22].

Previously, we examined the origins of stereoselectivity in intra‐ and intermolecular [2 + 2] cycloadditions activated by chiral iridium triplet sensitizers using density functional theory (DFT) calculations [10, 11, 23]. We also studied the activation barriers and rates of intramolecular [2 + 2] photocycloadditions assisted by Brønsted acids [24]. The rates of triplet energy transfer between the hydrogen‐bonded donor and acceptor moieties, however, have not been characterized yet, mainly due to experimental and theoretical challenges in precisely quantifying the rates of TTEnT between the photosensitizer and the substrate.

**FIGURE 1:**
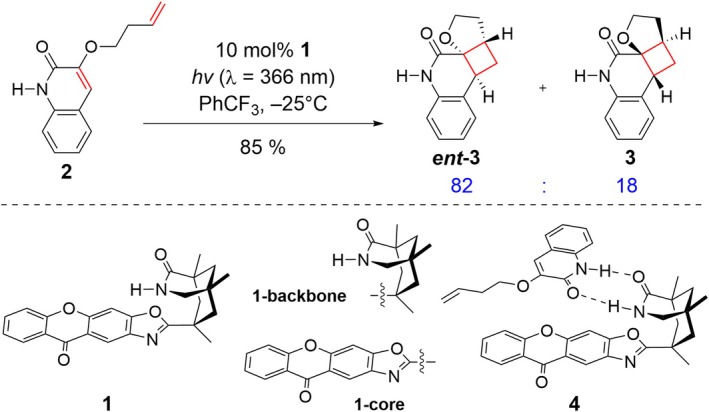
Enantioselective [2 + 2] cycloaddition reaction of **2** mediated by chiral organic photocatalyst **1**.

In this study, we investigate the detailed mechanism of the enantioselective intramolecular [2 + 2] photocycloaddition of an enone substrate **2** using the xanthone‐containing chiral organic photocatalyst **1**, previously reported by the Bach group (Figure 1) [4]. Photocatalyst **1** consists of an organic photoactive core **1‐core** and the lactam backbone **1‐backbone** that binds the substrate **2** using two hydrogen bonds to afford the encounter complex **4**. Quantum chemical analysis based on the uncontroversial structure of the donor–acceptor adduct enhances our intuition about how the donor and acceptor couple in the TTEnT process, which has been elusive in earlier examples [25, 26]. The organic photocatalyst **1** plays three roles: (i) as a photosensitizer that absorbs light energy and transfers it to the substrate; (ii) as a chiral catalyst that provides a stereospecific environment; (iii) as a facilitator of TTEnT that places the donor and acceptor pair in close proximity to ensure proper orbital overlap. Unlike previous computational mechanistic studies based on multireference ab initio calculations [25, 26, 27] we apply a simple two‐state description of the electronic coupling based on the fragment excitation difference (FED) method in combination with time‐dependent density functional (TD‐DFT) calculations to quantify the rates of TTEnT [28, 29]. Finally, we propose a novel design strategy that utilizes the TTEnT process to induce stereospecific outcomes.

## 2 Methods

### 2.1 Conformer Search on the Ground Electronic Manifold

We employed the CREST software developed by Grimme's group to generate viable encounter complexes of the substrate and photosensitizer in the ground state, namely, ***syn‐*4** and ***anti‐*4** [30]. The generated conformers were subsequently optimized using QChem v5.0 software at the B3LYP‐D3/6‐31G(d,p) level of theory (*vide infra*). The conformers within 10 kcal/mol from the lowest‐in‐energy structure were considered to find relevant excited states encounter complexes and to explore the excited state potential energy surfaces.

### 2.2 Electronic Structures and Energies of the Ground and the Excited State Species

We optimized the geometries of photocatalyst–substrate encounter complexes in the ground state using the DFT calculations. Specifically, we used Becke's three‐parameter hybrid functional (B3LYP) [31, 32] augmented with Grimme's dispersion correction (D3) [33, 34] together with the double‐zeta quality Pople basis set (6‐31G(d,p)) [35, 36]. The optimized structures of intermediates and the transition states are subsequently verified with frequency calculation, rendering zero and one imaginary frequency respectively, which ensured that the intermediates were located at the energy minima and the transition states were situated at the saddle point of the potential energy surface. In addition, solvation energy contributions were computed using a self‐consistent reaction field (SCRF), with the dielectric constant of the solvent used (trifluorotoluene, Ɛ = 9.18) [37, 38, 39]. Also, the single point electronic energies were computed using a triple‐zeta quality basis set (cc‐pVTZ(‐f)) [40]. We utilized Jaguar 9.1 software [41] for the purpose.

The Gibbs free energies of the optimized intermediates and transition states were calculated as follows:

(1) $$G\left(\mathrm{sol}\right)=G\left(\mathrm{gas}\right)+G\left(\text{solv}\right)$$

(2) $$G\left(\mathrm{gas}\right)=H\left(\mathrm{gas}\right)-\mathrm{TS}\left(\mathrm{gas}\right)$$

(3) $$H\left(\mathrm{gas}\right)=E\left(\mathrm{SCF}\right)+\mathrm{ZPE}$$

(4) $$\begin{array}{c} \Delta G\left(\mathrm{sol}\right)=\sum G\left(\mathrm{sol}\right)\;\text{for products}\;-\;\sum G\left(\mathrm{sol}\right)\;\text{for reactants} \end{array}$$

where $G\left(\mathrm{sol}\right)$ is the solution‐phase Gibbs free energy, $G\left(\mathrm{gas}\right)$ is the gas phase Gibbs free energy, $G\left(\text{solv}\right)$ is the solvation energy, $H\left(\mathrm{gas}\right)$ is the gas‐phase enthalpy, T is the temperature which is 298.15 K, $S\left(\mathrm{gas}\right)$ is the gas‐phase entropy, $E\left(\mathrm{SCF}\right)$ is the self‐consistent electronic energy, ZPE is the zero‐point energy, and $\Delta G\left(\mathrm{sol}\right)$ is the solution‐phase Gibbs free energy of an intermediate or a transition state structure, relative to the reactant(s). For the gas phase Gibbs free energy, enthalpy in the gas phase was added to the entropy correction $-\mathrm{TS}\left(\mathrm{gas}\right)$, which was derived from the frequency calculations. Finally, $H\left(\mathrm{gas}\right)$ is the summation of $E\left(\mathrm{SCF}\right)$ and ZPE, where the latter was computed from the frequency calculations too. Note that the entropy we refer to comprises the vibrational, rotational, and translational entropies of the solute(s), whereas the entropies of the solvent were implicitly incorporated in $G\left(\text{solv}\right)$. Computed energy components of the DFT optimized structures are summarized in Table S1.

We optimized geometries of excited state species based on the time‐dependent density functional theory (TD‐DFT) and investigated the rates of ISC and TTEnT. The excited state conformers were optimized using QChem v5.0 software at the TD‐CAM‐B3LYP‐D3/6‐31G(d,p) level of the theory. We used a range‐separated hybrid functional developed by Yanai et al. (CAM‐B3LYP) [42], complemented with Grimme's dispersion correction (D3), in combination with the double‐zeta quality Pople basis set (6‐31G(d,p)), namely TD‐CAM‐B3LYP‐D3/6‐31G(d,p) level of the theory. For the TD‐DFT calculations, we used QChem v5.0 software for the computations of excited state electronic structure [43].

In all optimized lowest‐in‐energy singlet excited state (S_1_) species, ***syn*‐5** and ***anti*‐5**, a predominant ^1^(n‐π*) transition character was observed on the photoactive core as described in Figure 5. The ***syn*‐6** and ***anti*‐6** conformers were obtained via the geometry optimizations following the triplet electronic manifold, which exhibited ^3^(π‐π*) activation characters localized at the photosensitizer. Decomposition of the identified excited state species into the canonical orbitals is summarized in Table S2. Also, the electronic energies of the excited state species are listed in Table S3.

We optimized 12 conformers for ***syn*‐6** and 5 conformers for ***anti*‐6**. Finally, we optimized ***syn*‐** and ***anti*‐7** conformers, characterized by ^3^(π‐π*) transition on the substrate. For the excited state structure optimizations, we utilized the STATE_FOLLOW option implemented in QChem software to follow the corresponding electronic surfaces. The lowest‐in‐energy conformers for each excited state were used to compute the rates for ISC and TTEnT. All optimized conformers for ***syn*‐** and ***anti*‐6** are analyzed in the paper.

We note that recent benchmark studies showed that TD‐DFT is a good compromise between the computational amenability and the accuracy for modeling excited‐state properties of sizable molecules [44, 45, 46]. Specifically, the method has been shown to predict excitation energies of low‐lying excited states with fidelity that is in excellent agreement with high‐level ab initio calculations.

### 2.3 Marcus Theory of Excited State Electronic Transition

**FIGURE 2:**
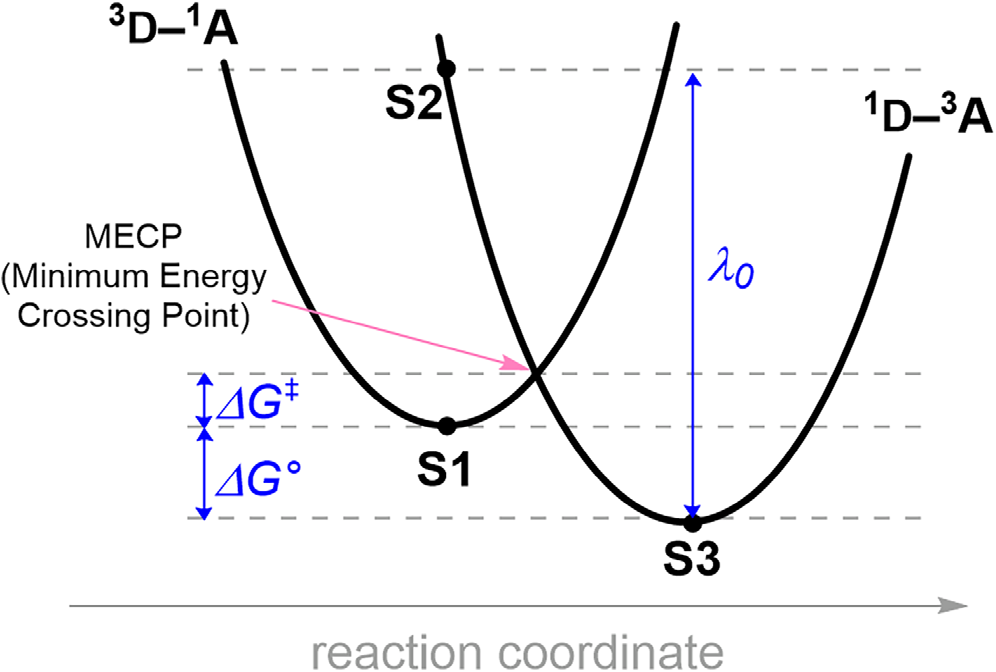
Diabatic potential energy surfaces of triplet‐triplet energy transfer (TTEnT).

The rates of ISC and TTEnT were computed on the basis of Marcus theory of electron transfer [47]. When an electronic transition proceeds from one diabatic electronic state to the other, for example from ^3^D‐^1^A to ^1^D‐^3^A (D‐donor and A‐acceptor), the process is described as a reaction on the hypothetical reaction coordinate that crosses over the minimum energy crossing point (MECP), which behaves as the activation barrier ($∆G^{‡}$) of the reaction. The activation barrier is determined using three states: S1 and S3, which represent the lowest‐energy geometries for the ^3^D‐^1^A and ^1^D‐^3^A states, respectively, and S2, which has the electronic structure of ^1^D‐^3^A but is in the relaxed geometry of ^3^D‐^1^A. The driving force ($∆G^{{}^{\circ}}$) is calculated from the energy difference between S1 and S3, while the reorganization energy (λ) is derived from the energy difference between S2 and S3, as illustrated in Figure 2. The activation barrier ($∆G^{‡}$) is derived by assuming that both potential energy surfaces are quadratic with the same curvature,

(5) $$∆G^{‡}=\frac{{\left(∆G^{{}^{\circ}}+\;\lambda \right)}^{2}}{4\lambda }$$

The Marcus rate is defined as follows:

(6) $$\begin{array}{c} k_{\text{Marcus}}=\frac{{\left|V\right|}^{2}}{\hbar }\sqrt{\frac{\pi }{\lambda k_{B}T}}\exp\left[\frac{-{\left(∆G^{{}^{\circ}}+\;\lambda \right)}^{2}}{4\lambda k_{B}T}\right] \end{array}$$

where the definition of each term is discussed in detail in the following sections. Of note, larger electronic coupling (V) and small effective activation barrier ($∆G^{‡}$) are the factors that enhance the rate of electronic transfer.

Despite its widespread use in physical science, the applicability of the Marcus equation in radiationless processes remains a topic of continued discussion [48]. Specifically, concerns have been raised that the Gaussian dependence on the energy gap (Δ*G*) contradicts the predictions deduced from the Fermi golden rule (FGR), which asserts an exponential decay of the resultant rate constant. However, Jang and Newton demonstrated that when Δ*G* is comparable to –λ, the Marcus formula serves as a valid approximate form derived from FGR [49]. In our case, the magnitudes of Δ*G* and –λ are comparable, as shown in Table 1 below. This suggests that the use of the Marcus formula would not significantly compromise the reliability of the computed rate constants.

**TABLE 1:** Detailed parameters for the computations of *k*_ISC_ and *k*_TTEnT_.

Transition	Rate constant (sec^−1^)	$V_{\mathrm{SOC}}$ or $V_{\text{TTEnT}}$ (cm^−1^)	$∆G^{{}^{\circ}}$(kcal/mol)	λ (kcal/mol)
*syn*‐5 → *syn*‐6	$1.4\times 10^{11}$	20	−13.61	13.59
*syn*‐6 → *syn*‐7	$3.7\times 10^{12}$	208	−10.36	17.53
*anti*‐5 → *anti*‐6	$6.8\times 10^{10}$	16	−15.40	17.27
*anti*‐6 → *anti*‐7	$4.0\times 10^{10}$	31	−9.93	19.19

### 2.4 Computations of the Rates of TTEnT

The TTEnT also known as Dexter energy transfer [20] can be rationalized as a concerted two‐electron transfer between triplet‐state donor and singlet‐state acceptor molecules. As such, the rate of TTEnT can be described employing Marcus theory of electron transfer as well:

(7) $$\begin{array}{c} k_{\text{TTEnT}}=\frac{{\left|V_{\text{TTEnT}}\right|}^{2}}{\hbar }\sqrt{\frac{\pi }{\lambda k_{B}T}}\exp\left[\frac{-{\left(∆G^{{}^{\circ}}+\;\lambda \right)}^{2}}{4\lambda k_{B}T}\right] \end{array}$$

where $V_{\text{TTEnT}}$ is the electronic coupling that is computed using the FED method implemented in QChem v5.0 [28, 29]. Here, λ is the reorganization energy and $∆G^{{}^{\circ}}$ is the energetic driving force, ℏ is the Planck constant divided by 2*π*, *k*_*B*_ is the Boltzmann constant, and *T* is the temperature, respectively. The electronic coupling is calculated between the initial and the final electronic states, using the initial state's geometry (i.e., between S1 and S2 in Figure 2) [50, 51].

With the structural ensemble of excited state geometries, we analyzed the Boltzmann‐weight averaged $V_{\text{TTEnT}}$ sampled from ***syn*‐6** and ***anti‐*6** shown in Figure 6. For each conformer, we used the Boltzmann factor of electronic energy as the statistical weight:

(8) $$\left\langle V_{\text{TTEnT}}\right\rangle =\frac{\sum_{i}\left|V_{i}\right|\exp\left(-\beta E_{i}\right)}{\sum_{i}\exp\left(-\beta E_{i}\right)}$$

(9) $$\mathrm{sd}\left(V_{\text{TTEnT}}\right)=\sqrt{\left\langle V_{\text{TTEnT}}^{2}\right\rangle }-{\left\langle V_{\text{TTEnT}}\right\rangle }^{2}$$

where $\left\langle V_{\text{TTEnT}}\right\rangle$ is the Boltzmann‐weight averaged coupling, $\beta =k_{B}T$ is the Boltzmann factor, *i* is the index of conformer, and $\mathrm{sd}\left(V_{\text{TTEnT}}\right)$ is the standard deviation of the computed couplings.

### 2.5 Computations of the Rates of ISC

The rates of ISC were computed on the basis of Marcus theory:

(10) $$\begin{array}{c} k_{\mathrm{ISC}}=\frac{{\left|V_{\mathrm{SOC}}\right|}^{2}}{\hbar }\sqrt{\frac{\pi }{\lambda k_{B}T}}\exp\left[\frac{-{\left(∆G^{{}^{\circ}}+\;\lambda \right)}^{2}}{4\lambda k_{B}T}\right] \end{array}$$

where $V_{\mathrm{SOC}}$ is the strength of spin‐orbit coupling (SOC). The method has been utilized to quantify ISC rates in organic electronic materials [52, 53]. We calculate the SOC between the singlet and triplet states at the relaxed geometry of the singlet state, using the Breit‐Pauli spin‐orbit Hamiltonian to simulate vertical excitation [54]. We used QChem v5.0 software to compute the strength of SOC.

## 3 Results and Discussion

**FIGURE 3:**
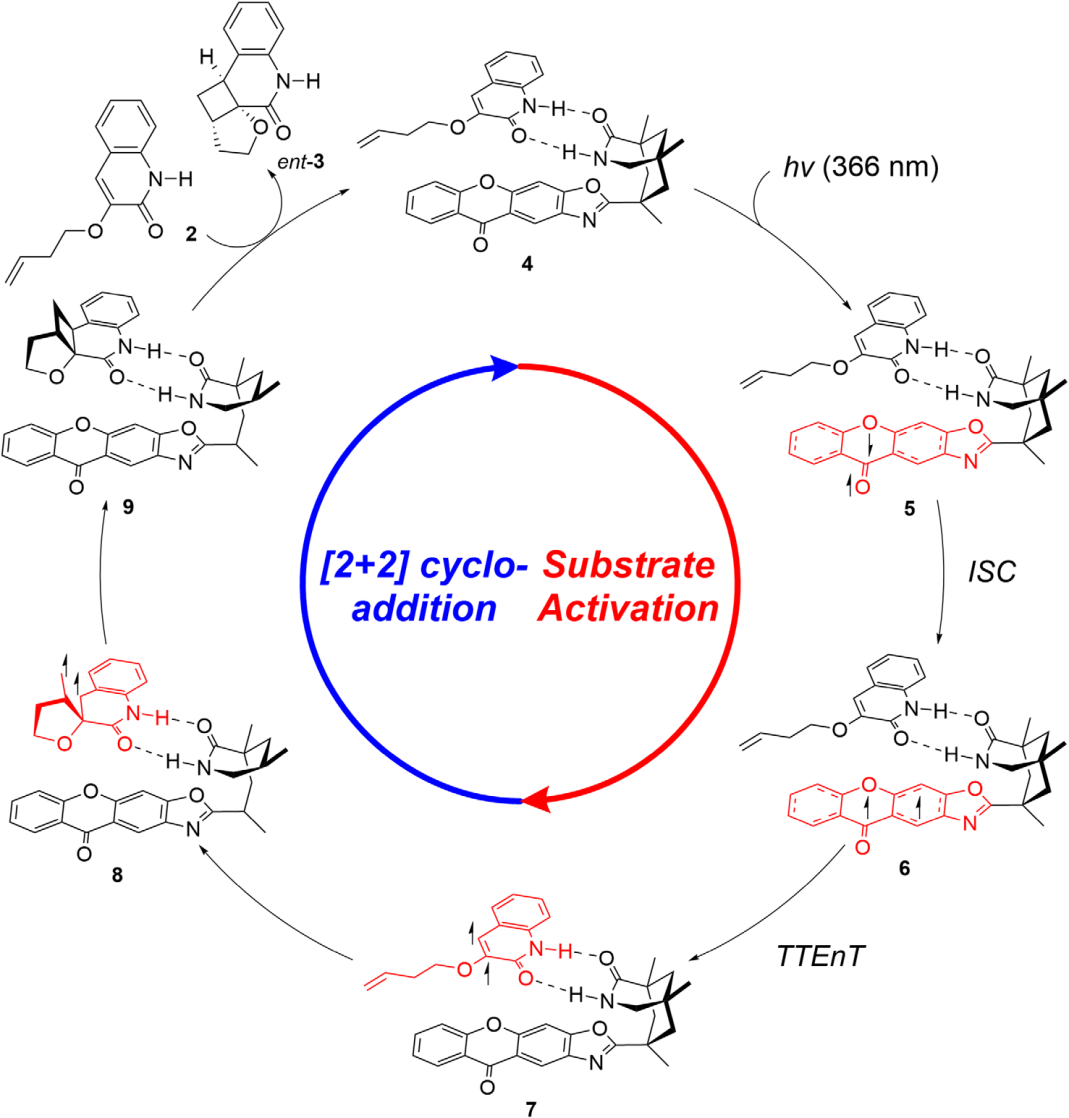
Proposed catalytic cycle of the [2 + 2] photocycloaddition reaction of **2** starting from the encounter complex **4**.

Figure 3 shows the proposed mechanism of the intramolecular [2 + 2] cycloaddition reaction mediated by the chiral organic photocatalyst **1**. The reaction begins with the association of **1** and **2** through hydrogen bonding to form the encounter complex **4**. Absorption of light by the photosensitizer **1‐core** [2] yields the first singlet excited species **5**, following Kasha's rule [55]. Subsequently, ISC gives access to the triplet species **6**, which can carry out the TTEnT to activate the substrate and form the triplet adduct **7**, enabling the enantioselective [2 + 2] cycloaddition to occur. The reaction proceeds via an intermediate **8**, which undergoes radical recombination to generate the product complex **9**. Finally, the product ***ent*‐3** is released, and another equivalent of substrate **2** binds to restart the catalytic cycle.

**FIGURE 4:**
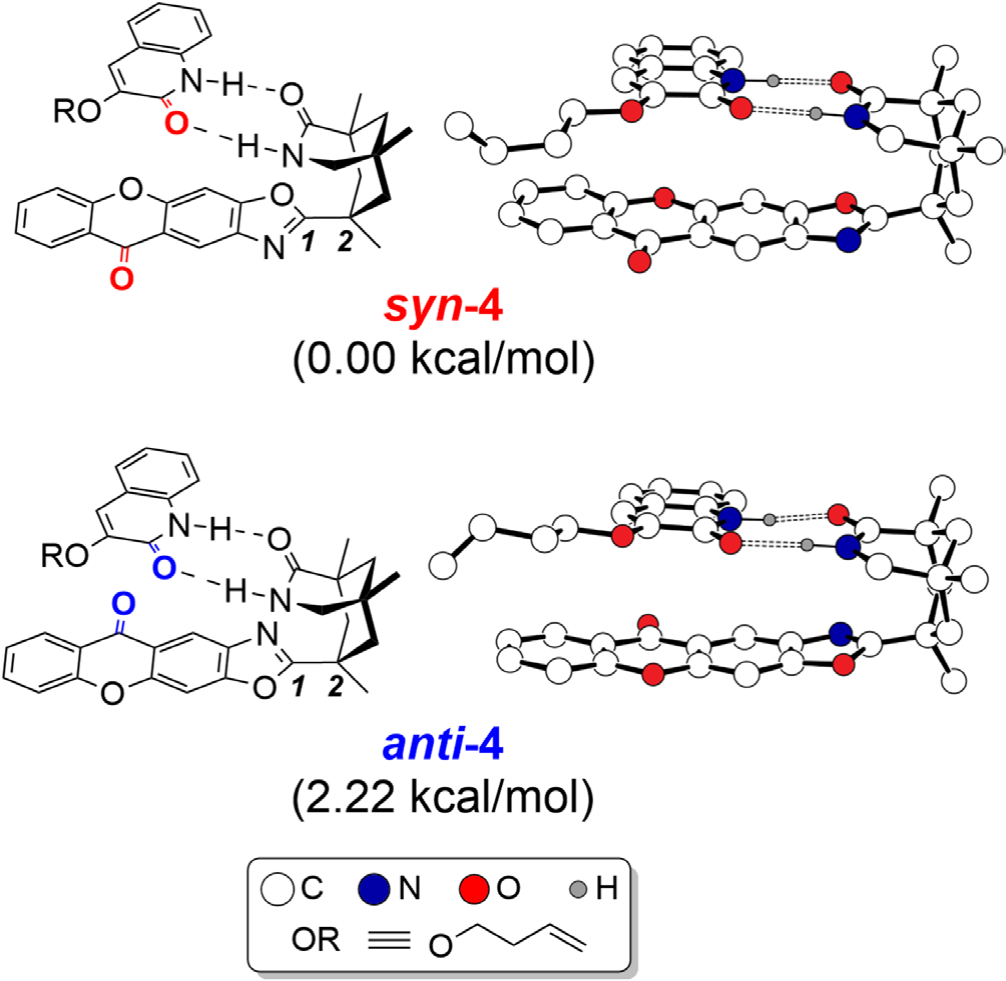
Viable structures of catalyst–substrate adducts optimized by DFT calculations. Hydrogens attached to carbons are omitted for clarity.

Viable structures of the catalyst–substrate encounter complex are shown in Figure 4. Experimentally observed enantioselectivity originates from the stereo‐selective binding of the substrate by a strategically placed hydrogen bond donor/acceptor pair on the chiral catalyst, ensuring that the substrate can only bind in a way that exposes the same molecular face to the photoactive **1‐core**, as illustrated for the encounter complex **4** in Figure 3. During the stereo‐selective [2 + 2] cycloaddition, the **1‐core** sterically protects one of the molecular faces of substrate **2** and forces the intramolecular [2 + 2] cycloaddition to proceed in a stereoselective fashion. While exploring possible structures for this key encounter complex, we realized that the xanthone‐containing fragment **1‐core** can freely rotate around the C1–C2 single bond that connects it to the **1‐backbone** when no substrate is present. As a result, both faces of **1‐core** can be exposed to substrate **2**, affording two distinct encounter complexes, labeled as ***syn‐*4** and ***anti‐*4**. The *syn* and *anti* denote the spatial arrangement of the carbonyl group of **1‐core** to that of **2**. The Gibbs free energy difference between the lowest energy structures of these two encounter complexes is 2.2 kcal/mol, favoring ***syn‐*4** with a predicted population ratio of 86:1 under equilibrium conditions at −25°C. The energetic preference of ***syn‐*4** was accredited to the conformational preference of photosensitizer **1**. When in the conformation of ***syn*‐4**, (***syn*‐1**), it is 2.2 kcal/mol lower in electronic energy (ΔE(SCF)) than that of ***anti‐*4** (Figure S1). The thermodynamic preference for the *syn*‐adduct suggests that there is a built‐in stereoselectivity for which of the two possible faces of xanthone will be in contact with the substrate. This is an additional stereochemical complexity that has not been appreciated thus far. In this specific case, exploiting this innate stereoselectivity is not possible as both encounter complexes produce the same enantiomer, ***ent‐*3**. That is, both ***syn‐*4** and ***anti‐*4** block the same face of the substrate and the stereoselective [2 + 2] cycloaddition will proceed in identical ways.

**FIGURE 5:**
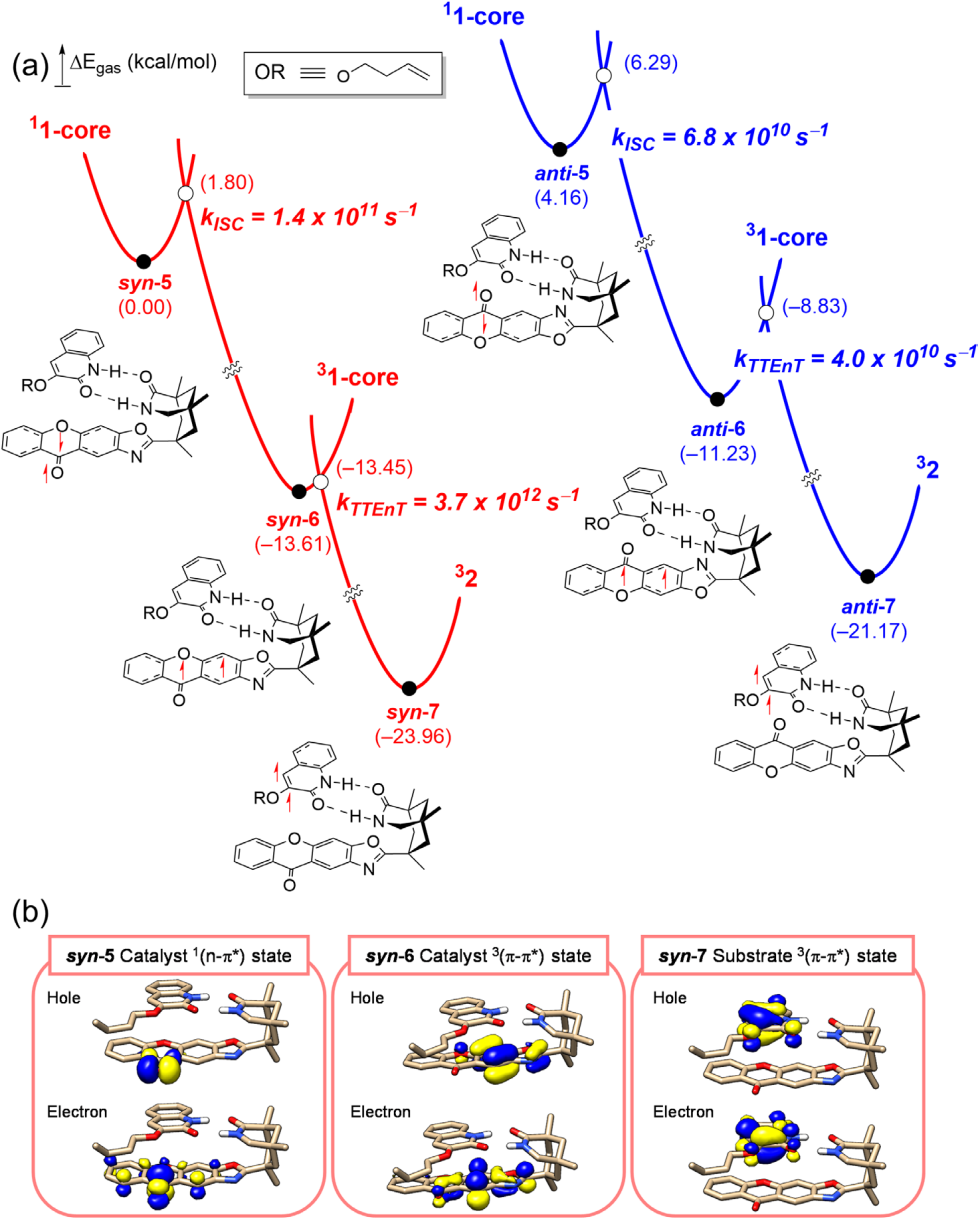
(a) Computed energy profile of the ISC and TTEnT steps and (b) the hole and electron pairs of natural transition orbitals of the three excited states.

Figure 5a depicts the energy profiles of the photophysical relaxation process leading to the formation of triplet excited state substrates. To compute the rates of ISC and TTEnT, we employed a combination of Marcus theory of electron transfer and TD‐DFT at the TD‐CAM‐B3LYP‐D3/6‐31G(d,p) level of the theory. Details of these calculations are provided in Methods. Upon UV/Vis irradiation, high‐energy singlet excited states are initially populated, subsequently relaxing to the first excited singlet state following Kasha's rule [55].

Two lowest‐in‐energy singlet excited state species, namely ***syn‐*5** and ***anti‐*5**, were optimized. The natural transition orbitals of ***syn‐*5**, visualized in Figure 5b, reveal that the excited state species bear characteristics of *n* → π* transitions, involving the transfer of an electron from the nonbonding orbital of carbonyl to the π* orbital of **1‐core**.

Moving forward, the intermediates ***syn‐*5** and ***anti‐*5** undergo ISC, transforming into the triplet species ***syn‐*6** and ***anti‐*6**, respectively. The calculated ISC rates from ***syn*‐5** to ***syn*‐6** and from ***anti‐*5** to ***anti‐*6** are 1.4 × 10^11^ s^−1^ and 6.8 × 10^10^ s^−1^, corresponding to activation barriers of 1.8 and 2.1 kcal/mol, respectively. Based on transition‐state theory of adiabatic reaction and the Eyring equation, the rate and activation energy barrier are correlated by the equation: *k* =(*k*_B_ *T/h*)exp(–*ΔG* ^*‡*^ */RT*), where *k* is the rate constant, *k*_B_ is the Boltzmann constant, *h* is the Planck constant, *ΔG* ^*‡*^ is the energetic barrier of activation, *R* is the gas constant, and *T* is temperature. Thus, the ISC rates are comparable for both the *syn‐* and *anti‐*isomers, highlighting that the electronic transition occurs within **1‐core**, independent of the structural orientation toward the hydrogen‐bonded substrate. These computed rates of ISC and the nature of the electronic transition that changes from ^1^(*n* → π*) to ^3^(π → π*) are in good agreement with previous reports on the photophysical properties of xanthone [56, 57, 58] and a recent computational study based on the wavepacket dynamics calculations [59].

Next, we investigated the rates of TTEnT from ***syn‐*6** to ***syn‐*7** and from ***anti‐*6** to ***anti‐*7**. The TTEnT involves a simultaneous exchange of two electrons, known as the Dexter energy transfer, where proper overlap between the frontier orbitals of the donor–acceptor pair is crucial. We hypothesized that the *syn*‐ and *anti*‐isomers would facilitate the TTEnT at different rates due to the difference in degrees of orbital overlap. Indeed, our calculations indicated that the TTEnT is approximately 100 times faster in the ***syn‐*6** than in the ***anti‐*6** isomer: the computed rates of TTEnT are 3.7 × 10^12^ s^−1^ and 4.0 × 10^10^ s^−1^, corresponding to activation barriers of 0.2 and 2.4 kcal/mol at −25°C, respectively (Figure 5a) [56]. Thus, the *syn‐*isomer is innately advantageous for TTEnT and likely responsible for the successful [2 + 2] photocycloaddition reaction. As mentioned above, both structural isomers produce the same product in this case. The innate facial selectivity, however, is an interesting feature that deserves additional attention in the future for designing other stereoselective versions of this useful reaction.

Comparing the three components of TTEnT within the framework of Marcus theory, namely, the electronic coupling ($V_{\text{TTEnT}}$), the thermodynamic driving force ($∆G^{{}^{\circ}}$), and the reorganization energy (λ), we found that the electronic coupling contributed the most to the difference in the rates of TTEnT (Table 1). Of note, the $V_{\text{TTEnT}}$ is a direct measure of orbital overlap between the initial and the final electronic states of TTEnT in the Marcus equation [19, 60]. For the *syn*‐adduct, the *V*_TTEnT_ between the initial and the final states of TTEnT (***syn‐*6** and ***syn‐*7**) is 208 cm^−1^, which is 7 times higher than that of the *anti*‐isomer (31 cm^−1^). The difference in the strengths of electronic coupling accounts for the 49‐fold higher rate of TTEnT in the *syn*‐adduct over the *anti‐*counterpart.

**FIGURE 6:**
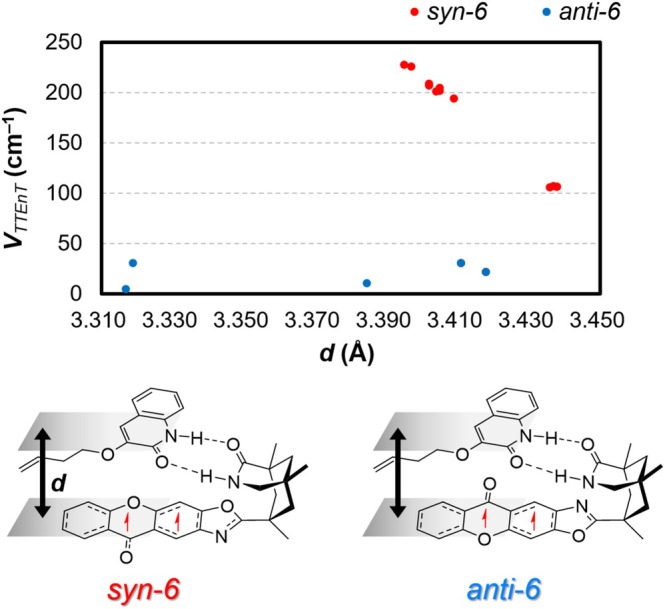
Relationship between coupling constant *V*_TTEnT_ and distance (*d*) between **1‐core** and **2**.

**FIGURE 7:**
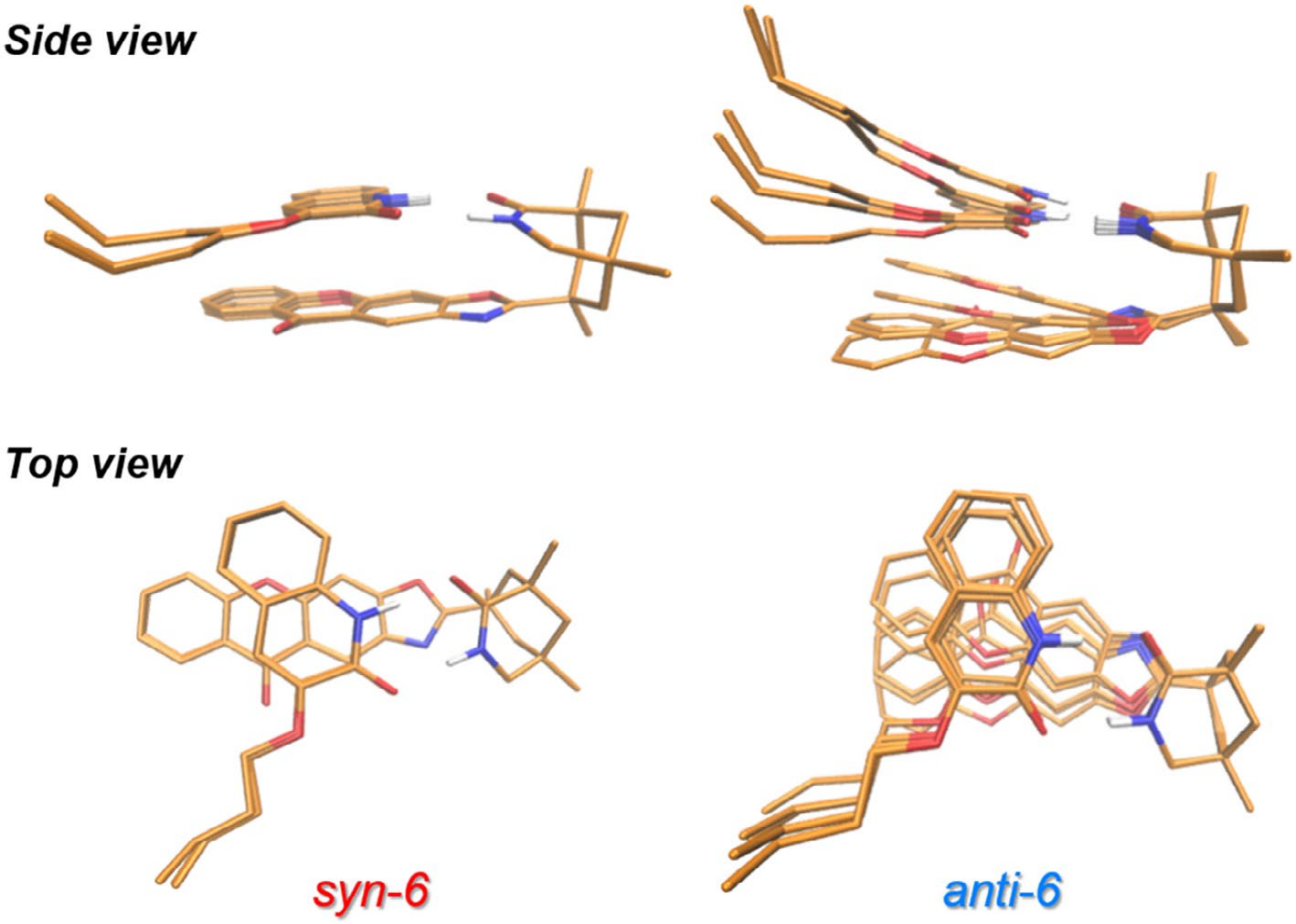
Overlapped structure of 12 conformers for ***syn*‐6** and 5 conformers for ***anti*‐6**, shown from side and top views.

We investigated how the structural differences of the catalyst–substrate encounter complexes influence the $V_{\text{TTEnT}}$. Figure 6 shows the distribution of $V_{\text{TTEnT}}$ computed from conformers of ***syn‐*6** and ***anti‐*6**. Previous investigations of intramolecular TTEnT revealed a strong correlation between the electronic coupling strength ($V_{\text{TTEnT}}$) and the intermolecular distances (***d***) between the triplet‐sensitizer and the acceptor [50]. Prompted by these findings, we computed $V_{\text{TTEnT}}$ from a set of *syn*‐ and *anti‐*conformers, varying the distance (***d***) between the triplet donor (**1‐core**) and acceptor **2**. To identify the conformational ensemble, we performed a conformational search, which yielded 12 local minima for ***syn‐*6** and 5 for ***anti‐*6**. We used the CREST software developed by Grimme's group for the conformational search. The conformational energies of the identified local minima are comparable to those of the lowest energy structure (Table S4). As the superposition of the 12 *syn*‐conformer structures illustrates (Figure 7), the structural difference between them is small with structural fluctuations in the branched alkene chain being the dominant difference. Thus, all optimized *syn*‐conformers exhibit nearly identical arrangements between **1‐core** and **2**. In contrast, the five conformers of ***anti‐*6** showed diverse arrangements between **1‐core** and **2**. The computed $V_{\text{TTEnT}}$ for *syn*‐conformers ranged from 106 to 228 cm^−1^, with a Boltzmann‐weighted average of 195 ± 26 cm^−1^. On the other hand, the maximum $V_{\text{TTEnT}}$ for *anti*‐conformers was 31 cm^−1^, and their Boltzmann‐weighted average was 30 ± 1 cm^−1^. Here, we used the Boltzmann factor of electronic energy ($\exp\left(-\Delta E\left(\mathrm{SCF}\right)/k_{B}T\right)$) as the statistical weight for each conformer. Comparing the statistically representative $V_{\text{TTEnT}}$ values with those of the lowest‐energy conformers (Table 1), we conclude that the $V_{\text{TTEnT}}$ values and TTEnT rates calculated from the most stable conformers are statistically significant. It is also noted that square averaged coupling constants ($\left\langle V^{2}\right\rangle$) were $3.89\pm 0.8\times 10^{5}$ cm^−2^ for ***syn‐6*** and 926±51 cm^−2^ for **anti‐6**, respectively. The finding suggested the fluctuation in $V^{2}$ would affect 20% and 5% of the computed rates of TTEnT for ***syn‐6*** and ***anti‐6***, respectively.

Interestingly, the *V*_TTEnT_ exhibited a linear correlation with the intermolecular distance (*d*) in *syn‐*conformers (Figure 6, red). However, such a dependence could not be observed for the *anti‐*conformers (Figure 6, blue). These observations suggest that the *syn‐*complexes possess a characteristic arrangement between **1‐core** and **2**, leading to higher *V*_TTEnT_ values and showing a linear correlation with the intermolecular distance *d*.

**FIGURE 8:**
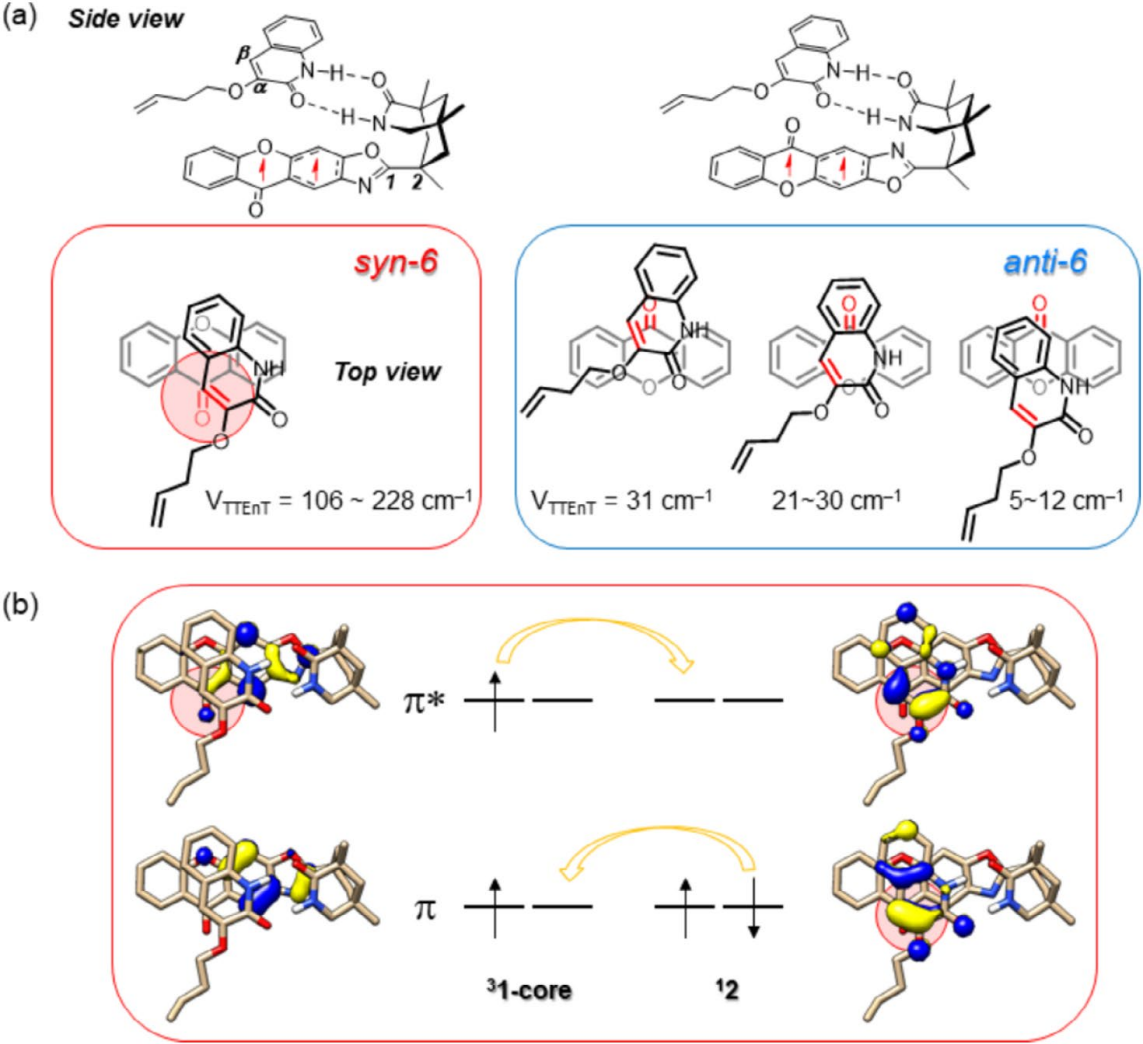
(a) Lewis structure representations of the optimized geometries for ***syn*‐6** and ***anti*‐6**, shown from the side and top views. (b) Top view of the natural transition orbitals involved in the two‐electron transfer process for ***syn*‐6**, with regions of strong orbital overlap highlighted in red circles.

The enhanced electronic coupling ($V_{\text{TTEnT}}$) in the *syn‐*adducts can be attributed to the favorable orbital overlap between the photosensitizer **1‐core** and the substrate **2**, which function as the triplet energy donor and the acceptor, respectively. The Lewis structures of ***syn*‐6** and ***anti*‐6** are illustrated in Figure 8a. As detailed in Figure 7, the arrangement of **1‐core** and **2** in ***syn*‐6** is unique. In contrast, their arrangement in ***anti*‐6** can be classified into three different structure types, none of which offers an ideal donor–acceptor orbital overlap. The natural transition orbitals of ***syn*‐6** before and after the TTEnT process are represented in Figure 8b. The TTEnT can be described as a concerted two‐electron exchange between **1‐core** and **2**: one electron in the π* of **1‐core** migrates to the π* orbital of C_α_=C_β_ double bond in **2** and one electron in the π orbital of C_α_=C_β_ double bond in **2** moves to the π level of **1‐core**. Consequently, the spatial proximity between the π and π* levels of **1‐core** and **2** increases the electronic coupling ($V_{\text{TTEnT}}$). In the *syn*‐conformer, the donor–acceptor orbitals show good spatial overlap, as the C_α_=C_β_ double bond of **2** aligns with the carbonyl π and π* orbitals of **1‐core**. On the contrary, the overlap is weak in the *anti‐*conformers as the double bond of **2** and the π and π* orbitals of the carbonyl group of **1‐core** are spatially separated (Figure 8a and Figure S2). As a consequence, the *syn*‐adducts demonstrate higher electronic couplings than the *anti‐*counterparts.

**FIGURE 9:**
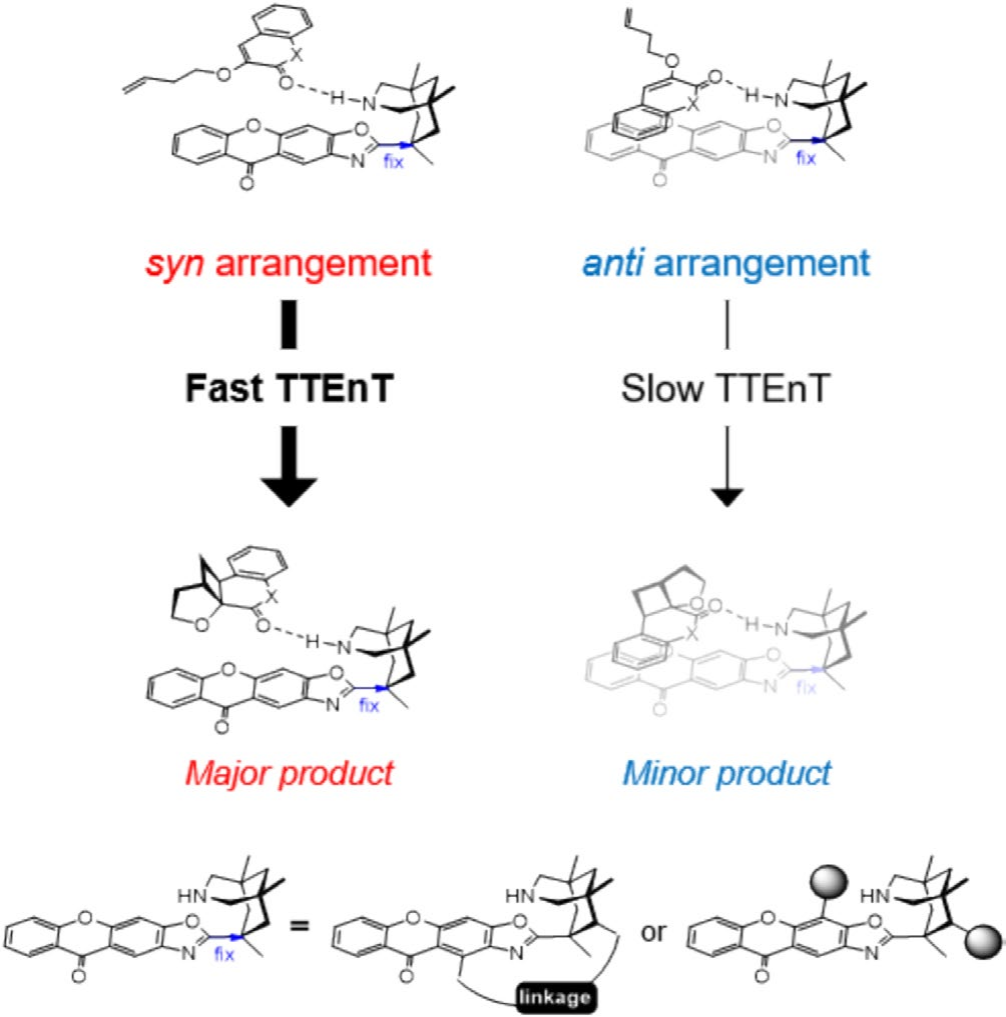
Design proposal for a chiral photocatalyst that utilizes different triplet‐triplet energy transfer rates.

Based on these findings, we propose a novel design principle for photocatalysts that leverages the intrinsic preference in the rate of TTEnT. By restraining the rotation of **1‐core** (Figure 9), the photocatalyst will induce asymmetric activation in favor of the *syn‐*adducts, making the [2 + 2] cycloaddition more favorable for the *syn*‐adducts over the *anti‐*adducts. This restraint can potentially be achieved by introducing an additional linkage between the **1‐core** and **1‐backbone** or by incorporating sterically bulky substituents. In this system, the **1‐backbone** does not require two hydrogen bonding sites for asymmetric induction. Instead, one hydrogen bonding site can arrange the substrate in both *syn*‐ and *anti*configurations, with the *si*‐face attack being facile. This is because the TTEnT process is 100 times faster in the *syn*‐adduct compared to the *anti‐*adduct due to efficient orbital interaction. As such, the cycloaddition in the *syn*‐encounter complex will be preferred, leading to enantioselective reactions.

**FIGURE 10:**
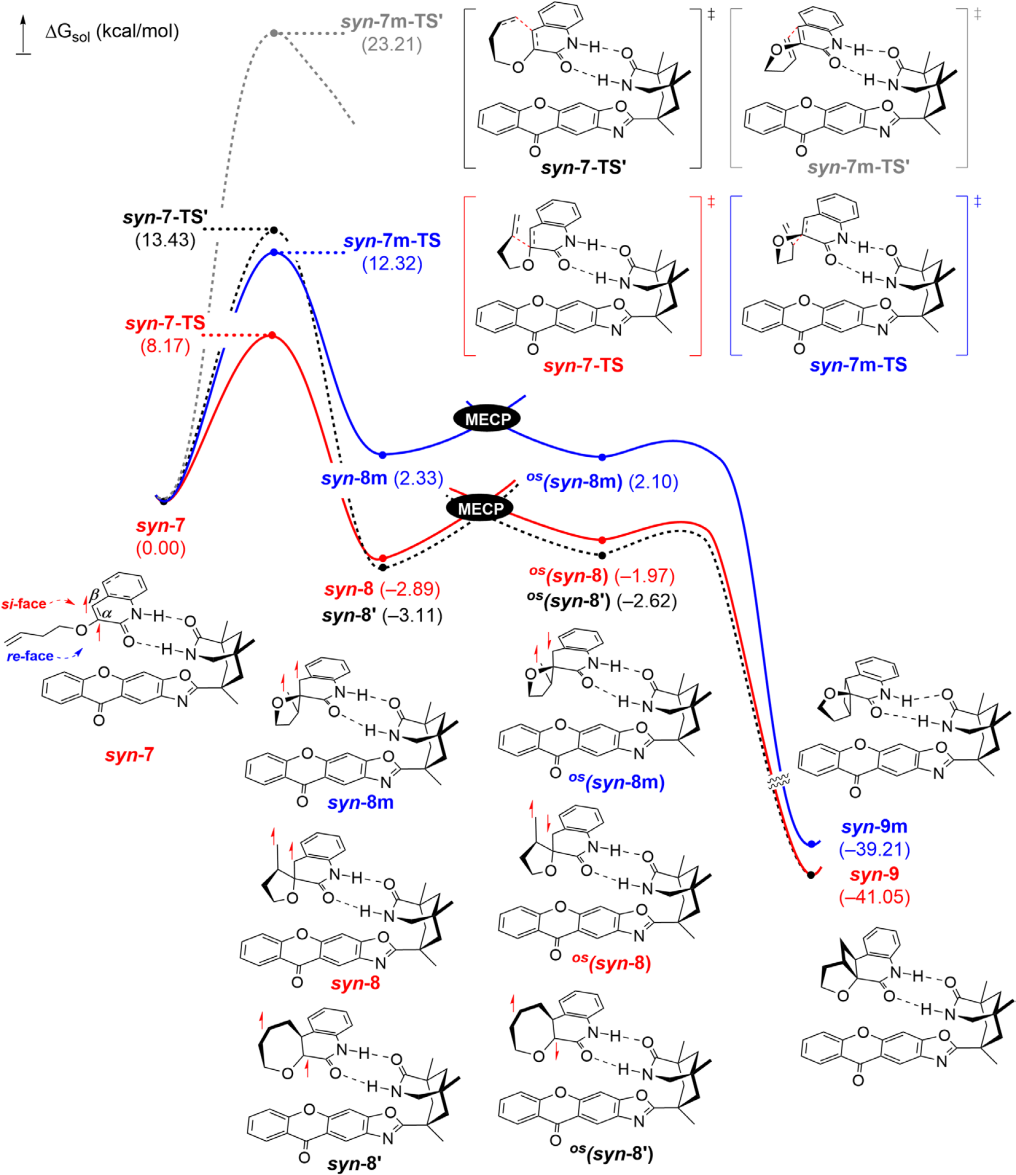
Calculated free energy profile for the bond‐forming steps of the enantioselective [2 + 2] cycloaddition.

Finally, we delved into the detailed bond‐forming steps in the enantioselective [2 + 2] cycloaddition. Upon completion of the TTEnT, the cyclization process ensues, during which the bond formation events take place, as shown in Figure 10. We optimized the structures of intermediates and the transition states at the UB3LYP‐D3/cc‐pVTZ(−f)//UB3LYP‐D3/6‐31G(d,p)/SCRF(ε = 9.18) level of theory. For each located structure, Gibbs free energy in the solution phase (ΔG_sol_) was computed, incorporating the solvation energy, the zero‐point energy, and the entropic contributions. (See Methods details). First, we followed the reaction trajectory leading to the major product shown in red. In ***syn‐7***, the vinyl moiety approaches the C_α_=C_β_ double bond on the quinolone ring from the *si*‐face, whereas the *re*‐face attack is hindered due to the steric constraint imposed by the photosensitizer. Our calculations suggest that the [2 + 2] cycloaddition occurs in a stepwise manner, forming the two C–C bonds sequentially, in good agreement with previous investigations [21, 22]. The preferred pathway involves bond formation between the internal carbon of the vinyl group and the Cα of the quinolone core, with an activation barrier of 8.2 kcal/mol (***syn*‐7‐TS**), yielding a five‐membered spirocyclic intermediate ***syn*‐8**. On the other hand, the coupling between the terminal carbon of the vinyl group and the C_β_ carbon of quinolone was predicted to be notably slower, with a barrier of 13.4 kcal/mol (***syn*‐7‐TS′**), resulting in a 7‐membered ring intermediate, ***syn*‐8′**. The lower energy of ***syn*‐7‐TS** over ***syn*‐7‐TS′** is attributed to the favorable orbital interaction of the former. As shown in Figure S3, the two pairs of π‐antibonding orbitals of the approaching alkenes show in‐phase overlaps in ***syn‐7‐TS***, while only a pair of π‐antibonding orbitals exhibit favorable interaction in ***syn‐7‐TS*′**. After the formation of the first C–C bond, ISC to the singlet manifold follows to yield the open‐shell singlet species, ^***os***^ ***(syn‐8)*** and ^***os***^ ***(syn‐8′)*** (Figure S4). We note that the MECP between the triplet and the open‐shell singlet species were not located here. Previously, the Zhong, and Liao group explored the mechanism of bond‐forming steps in the intramolecular [2 + 2] cycloaddition of a quinolone substrate. The authors located the MECP between the triplet and singlet 1,4‐diradical species which lies only 0.1 kcal/mol higher than the lowest‐energy triplet 1,4‐diradical species [22]. Finally, the ring‐closing step is calculated to be barrierless, leading to the formation of the cyclobutane functionality. This DFT result is in good agreement with CASSCF(6,6)/6‐31G* level of calculations that estimated the barriers of similar ring closing processes to be ~1 kcal/mol [16].

We also followed the reaction pathway of the *re*‐face attack, which resulted in a minor product. The computed activation barrier of the first bond‐forming step leading to a five‐membered ring intermediate ***syn*‐8 m** is 12.3 kcal/mol (***syn*‐7 m‐TS**), which is 4.1 kcal/mol higher than that of the major product (***syn*‐7‐TS**). The activation barrier for an eight‐membered intermediate was 23.2 kcal/mol (***syn*‐7 m‐TS′**), precluding its viability. In short, the comparison of the reaction energy profiles leading to the major and the minor product highlights the role of chiral constrict provided by the hydrogen bonding interaction between the chiral photosensitizer and the substrate.

Finally, we discuss the possibility of TTEnT in solution mediated by the random collision between the photosensitizer **1** and substrate **2**. Albeit the hydrogen‐bonded encounter complexation is highly likely the major interaction between the two, at an elevated temperature, the random collision between **1** and **2** may also mediate TTEnT. In such circumstances, poor stereoselectivity is expected as the substrate may proceed with the [2 + 2] cycloaddition in an achiral environment. Indeed, in the previous experiments, Bach and collaborators observed that the enantiomeric excess (*ee*) gradually decreased from 64% to 46% when the reaction temperature was elevated from −25°C to room temperature [4]. Also, many triplet sensitizers that do not mediate hydrogen bonding interaction have been shown to activate substrates via TTEnT. It is important to note that the hydrogen bonding interaction between the photocatalyst and the substrate is a crucial prerequisite in designing an effective photocatalyst, as it ensures a more controlled and efficient interaction between the two molecules. This fundamental aspect should be carefully considered in the future research and development of new photocatalysts.

## 4 Conclusions

In conclusion, we have elucidated the mechanism of the [2 + 2] photocycloaddition of enone **2**, catalyzed by the chiral organic photocatalyst **1**. We quantified the TTEnT process within a hydrogen‐bonded donor–acceptor structure. Through orbital analysis of this well‐defined structure, we demonstrated that the *syn*‐adduct adopts a geometry that maximizes orbital overlap between the carbonyl group of **1‐core** and the C_α_=C_β_ bond of **2**, which explains the stronger electronic coupling of the *syn*‐isomer compared to the *anti‐*isomer. Based on this analysis, we proposed a design principle: orbital overlap dictates the TTEnT rate, offering a means to control enantioselectivity. In addition, this work demonstrates the effectiveness of combining Marcus theory and time‐dependent DFT to investigate the underlying mechanisms of how excited states are accessed and what determines efficient coupling between photocatalysts and substrates.

Moving forward, this technical framework for modeling TTEnT processes can pave the way for additional research. For example, recent reports by the groups of Bach, Weaver, and Gilmore describe photocatalytic deracemization reactions of a racemic mixture of allene [61] and E/Z‐olefins [62, 63, 64]. These transformations exploit the TTEnT as the key mechanistic step that activates the double bonds, initiating the *cis*‐*trans* isomerization. The modeling framework and analysis methods presented in this work hold the potential to offer insight into the electronic driving forces governing energy transfer and chemical reaction patterns in such photocatalytic processes.

## Conflicts of Interest

The authors declare no conflicts of interest.

## Supporting information


**Data S1.** Supporting Information.

## Data Availability

The data that supports the findings of this study are available in the Supporting Information of this article.
