# Supplementary material for: Understanding the Mechanism of Triplet‐Triplet Energy Transfer in the Photocatalytic [2 + 2] Cycloaddition: Insights From Quantum Chemical Modeling
Source: J Comput Chem. 2025 Jun 11;46(16):e70155. doi: 10.1002/jcc.70155 (PMC12152828; doi:10.1002/jcc.70155)
Supplement: Supplementary file 1 — Data S1. Supporting Information. [file JCC-46-0-s001.pdf]

Supporting Information

Understanding the Mechanism of Triplet-Triplet Energy Transfer in the Photocatalytic [2+2] Cycloaddition: Insights from Quantum Chemical Modeling

Eunji Lee[a,b], Hyejin Moon[a,b], Jiyong Park,[b,a*] and Mu-Hyun Baik[b,a*]

[a]Department of Chemistry, Korea Advanced Institute of Science and Technology (KAIST), Daejeon, 34141, Republic of Korea
[b]Center for Catalytic Hydrocarbon Functionalizations, Institute for Basic Science (IBS), Daejeon, 34141, Republic of Korea
Email: jiyongpa@ibs.re.kr, mbaik2805@kaist.ac.kr

Table of Contents

Supporting Tables and Figures S2
TD-DFT Optimized Geometries S9
DFT Optimized Geometries S26
Computed Vibrational Frequencies S38

Supporting Tables and Figures

Table S1. Computed energy components for DFT-optimized structures.

	**E(SCF)/(eV)**<br><br>B3LYP-D3/cc-pVTZ(-f)	**ZPE/(kcal/mol)**<br><br>B3LYP-D3/6-31G(d,p)	**S(gas)/(cal/mol)**<br><br>B3LYP-D3/6-31G(d,p)	**G(solv)/(kcal/mol)**<br><br>B3LYP-D3/6-31G(d,p)/PB(ε=9.18)
***syn*-4**	-56785.926	432.09	233.89	-18.91
***anti*-4**	-56785.805	432.28	232.30	-20.14
***syn*-7**	-56781.472	427.84	241.71	-15.34
***syn*-7-TS**	-56781.294	428.65	232.76	-14.76
***syn*-7-TS'**	-56780.985	428.56	233.35	-16.35
***syn*-7m-TS**	-56781.125	429.25	232.51	-15.17
***syn*-7m-TS'**	-56780.681	429.82	230.46	-15.70
***syn*-8**	-56781.745	428.65	237.17	-14.08
***syn*-8'**	-56781.865	430.52	227.04	-16.44
***syn*-8m**	-56781.543	429.90	234.02	-15.73
**^{os}(*syn*-8)**	-56781.736	428.51	234.14	-14.15
**^{os}(*syn*-8')**	-56781.852	430.93	228.94	-16.10
**^{os}(*syn*-8m)**	-56781.543	429.51	233.49	-15.71
***syn*-9**	-56783.713	433.28	224.16	-15.39
***syn*-9m**	-56783.598	433.94	228.02	-15.71

Table S2. Decomposition of the identified excited state species of encounter complexes *syn*- and *anti*- **5**, **6**, **7**. The geometries were optimized at the TD-CAM-B3LYP-D3/6-31G(d,p) level of the theory. Canonical orbitals are shown at isodensity of 0.05. The orbital energies written in the parentheses are in eV.

State	From	(Amplitude)^2	To
***syn*-5** ([1]**1-core**)	HOMO–4 (–8.205) 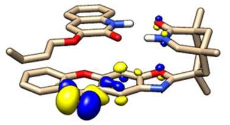	0.66	LUMO (–0.864) 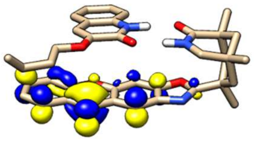
	HOMO–3 (–8.193) 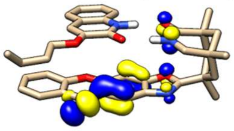	0.25	
***syn*-6** ([3]**1-core**)	HOMO–1 (–7.019) 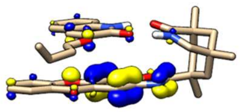	0.52	LUMO (–0.745) 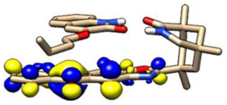
		0.10	LUMO+1 (–0.339) 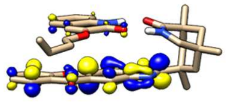
		0.05	LUMO+2 (–0.515) 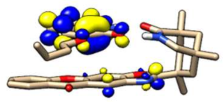
	HOMO (–6.645) 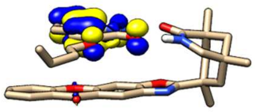	0.13	LUMO (–0.745) 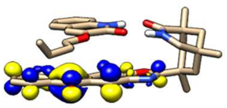
***syn*-7** ([3]**2**)	HOMO (–6.460) 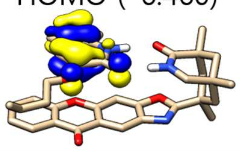	0.86	LUMO+1 (–0.208) 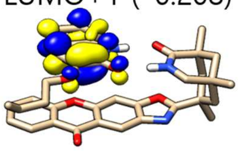

<table>
<tr><th>State</th><th>From</th><th>(Amplitude)^2</th><th>To</th></tr>
<tr><td rowspan="2">anti-5 (^{1}1-core)</td><td>HOMO–4 (–8.170)
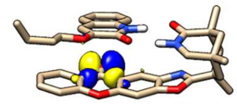</td><td>0.69</td><td rowspan="2">LUMO (–0.810)
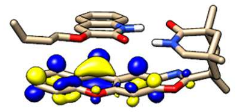</td></tr>
<tr><td>HOMO–1 (–7.167)
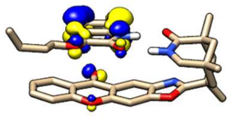</td><td>0.17</td></tr>
<tr><td rowspan="2">anti-6 (^{3}1-core)</td><td rowspan="2">HOMO–1 (–6.887)
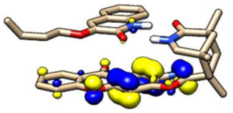</td><td>0.60</td><td>LUMO (–0.589)
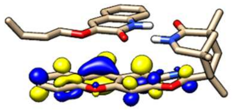</td></tr>
<tr><td>0.18</td><td>LUMO+1 (0.446)
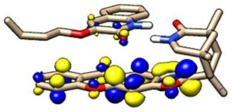</td></tr>
<tr><td>anti-7 (^{3}2)</td><td>HOMO (–6.329)
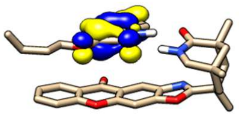</td><td>0.87</td><td>LUMO+1 (–0.025)
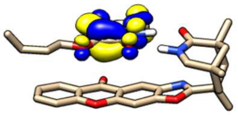</td></tr>
</table>

Table S3. Computed electronic energies for the lowest-in-energy conformers of TD-DFT optimized structures.

	***syn*-5**	***anti*-5**	***syn*-6**	***anti*-6**	***syn*-7**	***anti*-7**
E(SCF)/(eV)<br><br>TD-CAM-B3LYP-D3/6-31G(d,p)	–56734.734	–56734.553	-56735.324	–56735.221	–56735.773	–56735.652

Table S4. Comparison of electronic energies for conformers of ***syn*-6** and ***anti*-6**. The lowest-in-energy conformers for ***syn-6*** and ***anti-6*** are utilized to calculate rates of ISC and TTEnT.

***syn*-6** conformer	**1**	**2**	**3**	**4**	**5**	**6**	**7**	**8**	**9**	**10**	**11**	**12**
**ΔE_{gas}(rel)** (kcal/mol)	0.00	0.03	0.05	0.07	0.08	0.18	1.21	1.21	1.22	1.22	2.00	2.02

***anti*-6** conformer	**1**	**2**	**3**	**4**	**5**
**ΔE_{gas}(rel)** (kcal/mol)	0.00	2.62	3.29	6.21	6.46

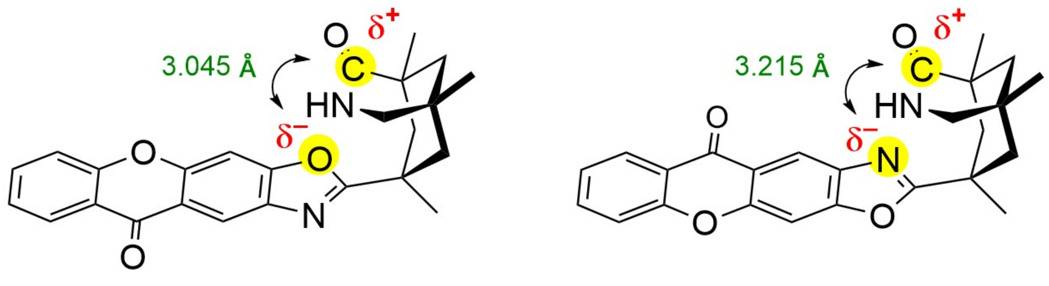

***syn*-1**
$\Delta\Delta E_{SCF}$ = 0.00 kcal/mol

***anti*-1**
$\Delta\Delta E_{SCF}$ = 2.21 kcal/mol

Figure S1. Electronic energy difference ($\Delta\Delta E_{SCF}$) of the two conformers of photocatalyst **1.** The syn preference in free energy is primarily due to the 2.2 kcal/mol lower SCF electronic energy (E_{SCF}) of ***syn*-1** compared to ***anti*-1**. Electronic structure analysis suggested a more favorable Coulombic interaction between **1-core** and **1-backbone** in ***syn*-1** than in ***anti*-1**. In ***syn*-1**, the carbonyl carbon of **1-backbone**, which bears a partial positive charge, faces the oxygen atom of **1-core** at a distance of 3.045 Å, whereas in ***anti*-1**, the distance to a partial negative nitrogen is 3.215 Å. The finding manifested a stronger Coulombic attraction in ***syn*-1**, making this configuration more electronically favorable.

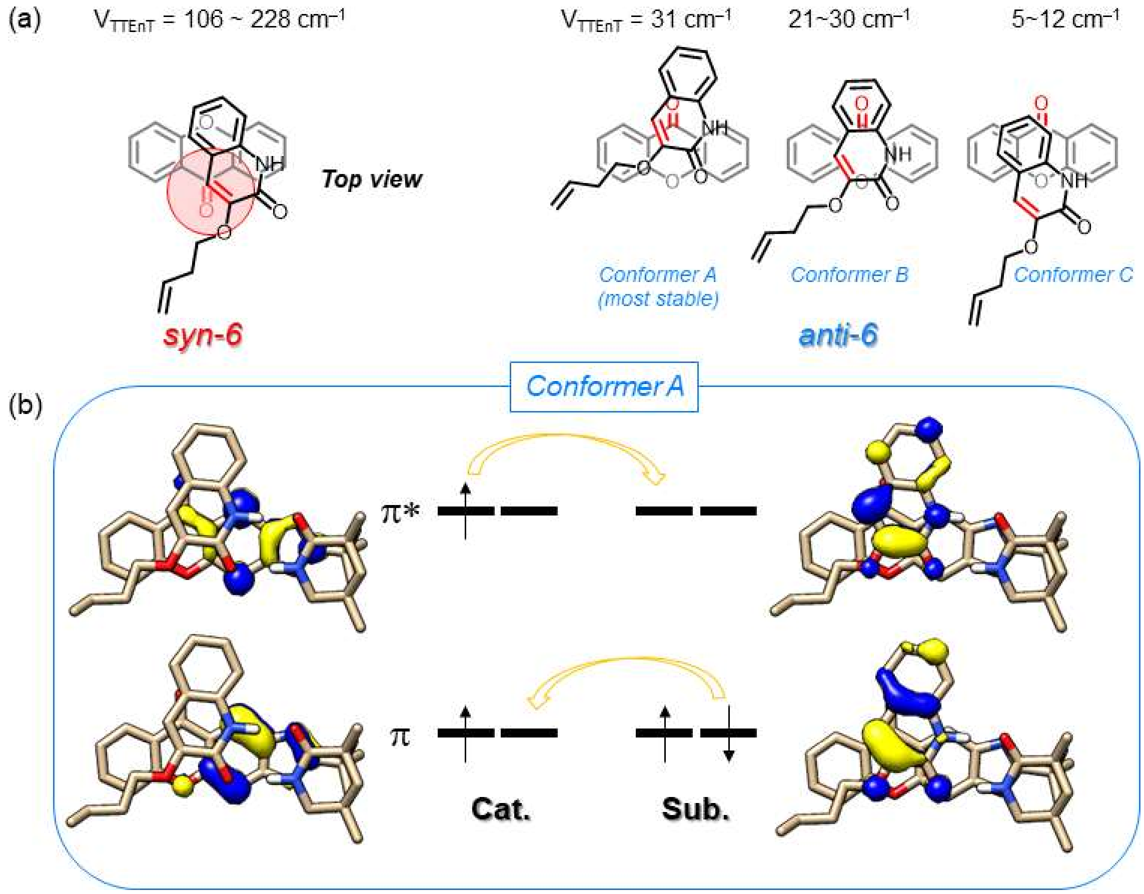

Figure S2. (a) Lewis structure representations of the optimized geometries for ***syn*-6** and ***anti*-6**, shown from top views, illustrating the distinctive donor-acceptor overlap in the syn-conformer with large coupling constant (V_{TTEnT}) and more varied overlap patterns in the anti-conformers with small V_{TTEnT}. (b) Top view of the natural transition orbitals involved in the two-electron transfer process for ***anti*-6** in its most stable conformer. The renditions show a lesser degree of orbital overlap between the donor and acceptor orbitals than ***syn*-6**.

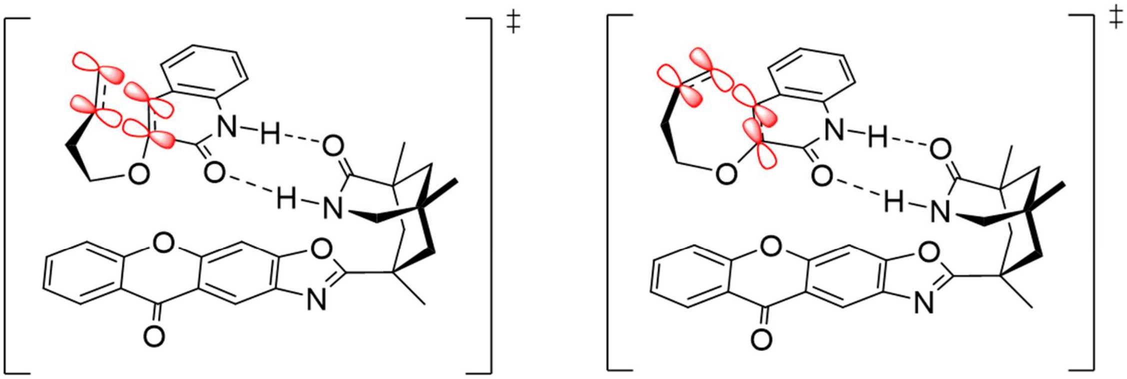

***syn*-7-TS**
SOMO

***syn*-7-TS'**
SOMO

Figure S3. Schematic illustration of SOMO orbitals participating in the first-bond forming step.

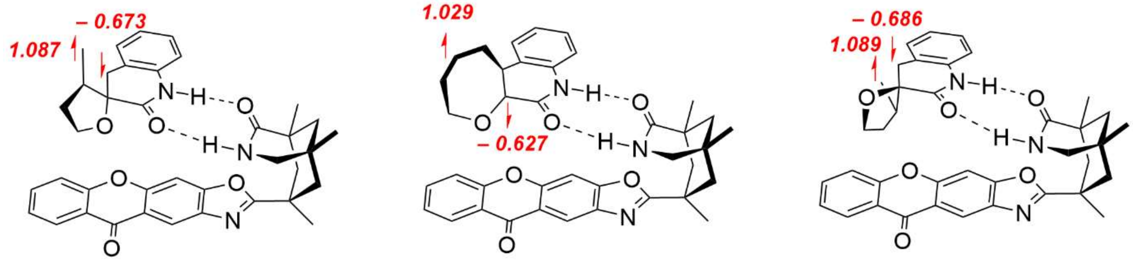

***os*(*syn*-8)** ***os*(*syn*-8')** ***os*(*syn*-8m)**

Figure S4. Mulliken spin densities of the identified open-shell intermediates

TD-DFT Optimized Geometries

================================
syn-5
================================

C	0.3144171317	-4.0186343842	-2.4065833795
C	0.1963716876	-3.1051088183	-3.6290605994
C	-0.8391854157	-2.0082567982	-3.3742129706
C	-0.4027120361	-1.1739947546	-2.1501839809
C	-0.0486676409	-1.9455549572	-0.8650547224
C	0.8093148623	-3.1809650829	-1.2060519284
H	-0.0905085457	-3.6917797328	-4.5103474699
H	1.1759797781	-2.6611119088	-3.8454894507
H	-1.1663145655	-0.4257576817	-1.9203068880
H	0.5015031861	-0.6287304632	-2.4455849859
H	0.9114443798	-3.8217194820	-0.3272821777
H	1.8131040297	-2.8218642490	-1.4611693530
C	1.3006009863	-5.1561690588	-2.6634735852
C	-0.9380214407	-1.0656094457	-4.5751188854
C	-1.0535758278	-4.6215104009	-2.0759165911
C	-2.2024038426	-2.6991616337	-3.2060555311
N	-2.1444561117	-3.9490395655	-2.4605116160
H	-3.0522127646	-4.3732191056	-2.2241581386
H	2.2968194625	-4.7519224099	-2.8654317038
H	1.3512878333	-5.8205358638	-1.8003848918
H	0.9922211046	-5.7526267332	-3.5264054626
H	-1.1774037541	-1.6174416443	-5.4888251236
H	0.0071086329	-0.5396117331	-4.7376819547
H	-1.7188507169	-0.3146351092	-4.4196070575
C	0.7361218810	-1.0265366560	0.0898143792
H	1.6755542930	-0.7094913141	-0.3715074963
H	0.9698279260	-1.5462317132	1.0229183773
H	0.1498149984	-0.1358560788	0.3300247940
C	-1.3017589008	-2.3452010690	-0.1399826518
O	-1.2020828362	-3.4217793146	0.7030465429
N	-2.4733478661	-1.8184259999	-0.2153077537
C	-5.1737303685	-3.6728257842	1.5812676504
C	-4.6431702932	-2.6399991518	0.7955176956
C	-4.3126636288	-4.6532821234	2.1470968600
H	-5.2905101923	-1.9066070422	0.3335860393
C	-3.2718395484	-2.6289324818	0.5875821893
C	-2.9470867422	-4.6461815854	1.9457940682
H	-2.3277671070	-5.4388454554	2.3432857362
C	-2.4709349107	-3.6181982229	1.1507381069
C	-8.4398618453	-5.1380273959	2.8327327497
C	-8.8476681721	-6.2566672121	3.5433641881
C	-7.0728267416	-4.9151795890	2.5840064805
H	-9.9043792199	-6.4199396067	3.7262458313
C	-7.9084614799	-7.1676172036	4.0192697292
C	-6.1394004417	-5.8406869246	3.0898584712
H	-8.2290725116	-8.0450432380	4.5697886137
C	-6.5495945189	-6.9564432773	3.7937767860
H	-5.7933391821	-7.6510121256	4.1402366788
H	-9.1662905307	-4.4283632432	2.4542821185
C	-6.5811358089	-3.8207790773	1.8122174829
O	-4.7998896800	-5.6857112143	2.8972606349
O	-7.4279559276	-2.9710109712	1.2898843833
H	-2.6097712226	-2.9166846676	-4.2010144816
H	-2.9051754286	-2.0333007823	-2.6981281563
O	-1.1330541038	-5.6871488726	-1.4441540143
C	-4.6589806442	-9.3617015452	1.6573133778
H	-5.5834618502	-9.7785769915	2.0459856475
C	-3.4384477958	-9.8687539925	2.0564251947
C	-4.7303511310	-8.2956152831	0.7490854066
C	-2.2541886151	-9.3191082675	1.5495934366
H	-3.3959651287	-10.6926010673	2.7607007271
C	-3.5338776739	-7.7580632081	0.2515254095
C	-5.9714739139	-7.7080704912	0.3216177660
C	-2.2944001942	-8.2706522080	0.6514790627
H	-1.2955818052	-9.7186250857	1.8635655264
H	-1.3895011960	-7.8290465274	0.2457590127
N	-3.5952936656	-6.7023358589	-0.6334171014
C	-4.7300716976	-6.0647768779	-1.0305770452
H	-2.7026214837	-6.3115357670	-0.9833312437
C	-5.9858672974	-6.6344000172	-0.5025268823
H	-6.8923331367	-8.1287660359	0.7061034927
O	-7.0717890260	-5.9608360553	-0.9217212470
O	-4.6994838770	-5.0836075878	-1.7784521713
C	-8.3441163939	-6.4328790821	-0.5068284565
C	-9.3722867442	-5.3960189372	-0.9248900951
H	-8.3666056717	-6.5609746192	0.5816356372
H	-8.5496716540	-7.4078762385	-0.9687540481
C	-10.7384118188	-5.7676912006	-0.4301420548
H	-9.0555118435	-4.4349666881	-0.5025001553
H	-9.3660937270	-5.2928611433	-2.0136684362
C	-11.7740117415	-6.0614835140	-1.2058174431
H	-10.8543912944	-5.8117338426	0.6529863568
H	-11.6947075409	-6.0341879927	-2.2892329960
H	-12.7393864862	-6.3340054442	-0.7922972428

================================
syn-6(conformer1/12)
================================

C	0.295306792	-4.012137206	-2.515483683
C	0.230028493	-3.036335021	-3.691528248
C	-0.853091566	-1.986246493	-3.438211449
C	-0.557278071	-1.243315093	-2.113568707
C	-0.160766212	-2.068354583	-0.870245170
C	0.763287332	-3.240912810	-1.266932642
H	0.010095837	-3.575482998	-4.620745747
H	1.209216198	-2.559552452	-3.821736219
H	-1.399819661	-0.591886669	-1.862043925
H	0.295036380	-0.585397801	-2.317033382
H	0.869852126	-3.927980266	-0.423979600
H	1.758597092	-2.835570265	-1.482621049
C	1.266896505	-5.157135672	-2.798653266

C -0.877742240 -0.945450940 -4.560478128
C -1.089542203 -4.610233983 -2.244915184
C -2.197469745 -2.723942529 -3.453392201
N -2.152611225 -4.020111284 -2.796458772
H -3.069337572 -4.454522765 -2.617319212
H 2.278247912 -4.765972668 -2.943559538
H 1.271339129 -5.865969312 -1.969855790
H 0.978041405 -5.698021680 -3.704128787
H -1.018708637 -1.423193113 -5.534105848
H 0.060421654 -0.384413503 -4.594153926
H -1.693464135 -0.230677745 -4.413230306
C 0.583449301 -1.149459499 0.122159143
H 1.504333604 -0.771105050 -0.330048235
H 0.843004796 -1.693105787 1.033394695
H -0.040346670 -0.294257805 0.396613076
C -1.355547043 -2.600668162 -0.145283551
O -1.111964592 -3.442548914 0.896320410
N -2.651303658 -2.387828746 -0.351355714
C -5.058703039 -4.279105303 1.820616611
C -4.704070473 -3.388529903 0.768523846
C -4.062367708 -4.878285835 2.586617289
H -5.465422930 -2.979212956 0.121359477
C -3.284836560 -3.150963645 0.556532122
C -2.644816176 -4.669381874 2.378823045
H -1.924489905 -5.202752833 2.980090106
C -2.337136498 -3.810826316 1.349568999
C -7.965248222 -5.997732866 3.500562895
C -8.154499746 -6.902834640 4.527021112
C -6.677893959 -5.567335836 3.160528454
H -9.156096433 -7.231206281 4.783380654
C -7.056132630 -7.399450644 5.240858761
C -5.604027867 -6.080300553 3.885506531
H -7.208201162 -8.111744921 6.044372039
C -5.773848947 -6.989627270 4.922555393
H -4.899878589 -7.365033840 5.441218696
H -8.792686574 -5.592712622 2.928866726
C -6.452066353 -4.629324316 2.052581294
O -4.317605374 -5.723616727 3.587704301
O -7.377362300 -4.195855301 1.360073654
H -2.508109269 -2.876288321 -4.493882706
H -2.968152692 -2.125061095 -2.960148571
O -1.200545739 -5.618834733 -1.525690424
C -5.122476132 -8.667118038 1.817023479
H -6.089930854 -8.990039232 2.189637470
C -3.959154426 -9.106774559 2.416578233
C -5.079434178 -7.780239451 0.731233961
C -2.717000620 -8.670732702 1.939644034
H -4.008722411 -9.787893943 3.259295587
C -3.824415779 -7.354136530 0.265708370
C -6.257776684 -7.263448719 0.090606996
C -2.642943677 -7.799595145 0.870282230
H -1.803986258 -9.017953814 2.411680244
H -1.692687116 -7.445307004 0.483640547
N -3.773157293 -6.471974147 -0.792181646
C -4.852329586 -5.927564893 -1.424138069
H -2.841680954 -6.169895691 -1.125752651
C -6.167957665 -6.372670155 -0.925768302
H -7.221802793 -7.579730881 0.468883469
O -7.186287568 -5.794759189 -1.576860326
O -4.723511980 -5.113970456 -2.342454987
C -8.498965514 -6.043994909 -1.091447313
C -9.461353410 -5.250178688 -1.957575181
H -8.560133051 -5.727217592 -0.043664400
H -8.726779742 -7.116704767 -1.157651281
C -10.873731232 -5.415631363 -1.480329295
H -9.160948721 -4.197886071 -1.912330085
H -9.363931648 -5.577054940 -2.997109717
C -11.834561680 -6.028439712 -2.160389620
H -11.092451006 -5.012084518 -0.492122537
H -11.651012580 -6.445402824 -3.147144276
H -12.839312371 -6.132512604 -1.764131265

==============================
syn-7
==============================

C 0.341182285 -4.000332650 -2.389242838
C 0.219044017 -3.102335635 -3.623181239
C -0.830294000 -2.014088764 -3.387169558
C -0.412499394 -1.160627461 -2.170088420
C -0.058234766 -1.913962850 -0.874484928
C 0.818538268 -3.141408961 -1.196793209
H -0.057094032 -3.702715552 -4.498669819
H 1.194747133 -2.650140906 -3.840520821
H -1.186625743 -0.418969624 -1.954509724
H 0.487394892 -0.607882629 -2.464736024
H 0.926817898 -3.770084094 -0.310225125
H 1.817755979 -2.769868608 -1.451848142
C 1.343497547 -5.127991045 -2.625812783
C -0.931734777 -1.086310476 -4.599395616
C -1.021737542 -4.618334412 -2.060735426
C -2.186234176 -2.719200952 -3.219990708
N -2.117748292 -3.957253122 -2.456532210
H -3.020211544 -4.391456037 -2.225023048
H 2.336681878 -4.714234436 -2.823621657
H 1.393944450 -5.782569387 -1.755225701
H 1.049833837 -5.737775866 -3.484557203
H -1.159528079 -1.651088872 -5.508121496
H 0.008744438 -0.552059039 -4.762184149
H -1.721603564 -0.342146435 -4.457383414
C 0.705435690 -0.975642065 0.078665122
H 1.644593084 -0.651376231 -0.377969638
H 0.938172454 -1.483214288 1.018686659
H 0.106113475 -0.090022625 0.305081473
C -1.311149601 -2.323822896 -0.155323759
O -1.198279585 -3.401078155 0.691858711
N -2.487034707 -1.814277031 -0.237954968

C -5.161447678 -3.727539377 1.530660980
C -4.646810894 -2.688307350 0.750262265
C -4.297848094 -4.686934319 2.095119387
H -5.333110244 -1.984149552 0.296509516
C -3.282105403 -2.639858087 0.556705629
C -2.920121157 -4.659238492 1.907761571
H -2.289959766 -5.446625299 2.298293944
C -2.464673336 -3.624383537 1.125994332
C -8.395175843 -5.244450577 2.776171195
C -8.791828910 -6.347233637 3.504430153
C -7.039278471 -5.011043579 2.516738243
H -9.843214471 -6.524894829 3.702503649
C -7.825921296 -7.240224308 3.988112880
C -6.093744966 -5.900170477 3.025111024
H -8.134148753 -8.114129706 4.552787463
C -6.481713849 -7.023142717 3.756656213
H -5.715847246 -7.706144672 4.104078055
H -9.108574255 -4.531377958 2.377230423
C -6.618143796 -3.861951342 1.697346407
O -4.761317843 -5.739076833 2.830261937
O -7.422778557 -3.094121648 1.187525357
H -2.582784989 -2.954753553 -4.215292763
H -2.901441525 -2.054801516 -2.727399866
O -1.096369247 -5.678214747 -1.422365283
C -4.721916590 -9.389748832 1.692469295
H -5.646619554 -9.841107975 2.037680816
C -3.508057121 -9.821840462 2.166050484
C -4.809339933 -8.327166764 0.736306473
C -2.318195586 -9.226061973 1.716103283
H -3.465966235 -10.628208796 2.890911226
C -3.569774207 -7.747345588 0.278004459
C -6.008553700 -7.841839083 0.252862020
C -2.356147958 -8.194026676 0.776330791
H -1.362185851 -9.571944782 2.093116213
H -1.444267419 -7.734672561 0.407687982
N -3.607208406 -6.754842693 -0.679970619
C -4.762654287 -6.103422424 -1.091070905
H -2.715586408 -6.352898260 -0.999869752
C -5.997112495 -6.657799614 -0.618644782
H -6.942677873 -8.296595138 0.550754823
O -7.091997575 -5.961795514 -0.931208807
O -4.685578930 -5.100367690 -1.818797323
C -8.383097594 -6.436298708 -0.545871451
C -9.371282516 -5.313254746 -0.808039390
H -8.387722546 -6.697290776 0.515614990
H -8.631440524 -7.330686055 -1.129279859
C -10.740244345 -5.697965960 -0.330087110
H -9.009515423 -4.424412432 -0.277961712
H -9.383304903 -5.080234746 -1.876447318
C -11.797333999 -5.874968551 -1.112179519
H -10.840804891 -5.854952699 0.744072955
H -11.735939090 -5.731227207 -2.187527480
H -12.764075723 -6.160706065 -0.710979815

================================
anti-5
================================

C 0.4151839134 -3.9054700717 -2.2721425674
C 0.1822049994 -3.0447548534 -3.5172185463
C -0.9699556048 -2.0683901221 -3.2751404791
C -0.6461379853 -1.1774383148 -2.0543559746
C -0.1300117626 -1.8575479205 -0.7697159513
C 0.8672408462 -2.9810790047 -1.1218577372
H -0.0487132710 -3.6812630628 -4.3802393386
H 1.1027461766 -2.4995928964 -3.7593504349
H -1.5097125031 -0.5474898408 -1.8188230215
H 0.1509253265 -0.4955517369 -2.3735369983
H 1.0714872902 -3.5716629954 -0.2265691572
H 1.8091118904 -2.5156520332 -1.4355344586
C 1.4974005617 -4.9547401031 -2.5228857981
C -1.1694644456 -1.1502467477 -4.4837346076
C -0.8836811082 -4.6385616953 -1.9018438811
C -2.2408901770 -2.9130177658 -3.1168498372
N -2.0454067867 -4.1153100076 -2.3244043629
H -2.8888676371 -4.6758153364 -2.1466778180
H 2.4462920331 -4.4663210006 -2.7633376961
H 1.6294691673 -5.5823390423 -1.6410349198
H 1.2264895200 -5.6058912865 -3.3585889142
H -1.3434531228 -1.7310293457 -5.3942187529
H -0.2883441473 -0.5234259435 -4.6478073322
H -2.0295421691 -0.4893769964 -4.3351948683
C 0.5647760972 -0.8095389268 0.1178256900
H 1.4175834122 -0.3692533579 -0.4065481862
H 0.9211504935 -1.2705440069 1.0414873715
H -0.1265456886 -0.0037993129 0.3820940502
C -1.2288745334 -2.4700308944 0.0531019984
O -2.5302620490 -2.1436599507 -0.2299281672
N -1.0759759418 -3.3462093755 0.9822649749
C -4.2073294235 -4.9951662749 2.1527927992
C -2.8308623299 -4.7321323253 2.1575213114
C -5.0811733139 -4.2178231401 1.3453691977
H -2.1528733254 -5.3491478570 2.7307312289
C -2.3678381810 -3.7215552667 1.3282352772
C -4.6295232913 -3.1925085240 0.5387123797
H -5.3146408809 -2.6531376138 -0.1012107017
C -3.2605611721 -2.9838948361 0.5565183865
C -6.7753278598 -7.5136384460 3.3399751938
C -8.1178829483 -7.7799597518 3.1193107331
C -6.1634176167 -6.4008172123 2.7367023081
H -8.5781684546 -8.6423769018 3.5897034159
C -8.8743858136 -6.9466376100 2.2998532468
C -6.9481409655 -5.5672003807 1.9176712572
H -9.9248431833 -7.1548444148 2.1276082062
C -8.2853305146 -5.8353018130 1.6974940946
H -8.8443970075 -5.1719387959 1.0476893231
H -6.1743618199 -8.1644687650 3.9630290176

C	-4.7751583101	-6.0961291033	2.8754491324
O	-6.4233340104	-4.4718513196	1.2961091523
O	-3.9998480630	-6.8659405337	3.5867380742
H	-2.5902411281	-3.2101816226	-4.1133918063
H	-3.0370395843	-2.3203946510	-2.6572495099
O	-0.8555603767	-5.6814205468	-1.2336880513
H	-5.0876623674	-10.1310193952	2.1889676864
C	-4.2098351061	-9.5480680163	1.9269781548
C	-3.0210265622	-9.7237282967	2.6091642995
C	-4.3074237145	-8.6180387314	0.8815627182
C	-1.9017799028	-8.9609022070	2.2647744247
H	-2.9591088270	-10.4460294775	3.4156420099
C	-1.9721229845	-8.0297517575	1.2451735836
H	-0.9700213651	-9.0924523873	2.8043680605
C	-3.1750512725	-7.8622303573	0.5489891180
H	-1.1150628053	-7.4269545246	0.9618176253
N	-3.2689229040	-6.9291584017	-0.4622398973
C	-5.5209734396	-8.3862387411	0.1481272560
C	-5.5844769476	-7.4339467147	-0.8118992089
H	-6.3925724420	-8.9735526600	0.4090906998
C	-4.4042682863	-6.6126993612	-1.1461414893
O	-6.6646110609	-7.1229192173	-1.5533671592
O	-4.4295032485	-5.7053233710	-1.9823951835
H	-2.4109106998	-6.4186721239	-0.7336355103
C	-7.8168034302	-7.9349599304	-1.3944025036
C	-8.8495803763	-7.4735375752	-2.4084558362
H	-8.2046834837	-7.8427388885	-0.3709915154
H	-7.5597491100	-8.9888198267	-1.5667581457
C	-10.1157223302	-8.2689353653	-2.2863928447
H	-9.0441908412	-6.4088094868	-2.2367747093
H	-8.4284993207	-7.5684806767	-3.4134237069
C	-10.5829164535	-9.0942077764	-3.2145296800
H	-10.6612236085	-8.1600229255	-1.3489949609
H	-10.0661427324	-9.2337209673	-4.1603358771
H	-11.4986103260	-9.6579158389	-3.0698142239

==============================

anti-6 (conformer1/5)

==============================

C	0.509972535	-3.862885630	-2.235550134
C	0.309058665	-2.964196966	-3.457867010
C	-0.889113940	-2.039668621	-3.241288505
C	-0.634873890	-1.172557390	-1.987468255
C	-0.201735345	-1.891991915	-0.693161137
C	0.846189440	-2.981160403	-1.013638155
H	0.149415402	-3.576211384	-4.353956094
H	1.218659845	-2.375908291	-3.630289485
H	-1.507491170	-0.543072236	-1.787267309
H	0.182735583	-0.489274709	-2.244758133
H	0.993917865	-3.611866258	-0.133823693
H	1.798295340	-2.481000085	-1.226321852
C	1.648418874	-4.855545791	-2.465708559
C	-1.074318380	-1.097534108	-4.433160779
C	-0.771109033	-4.650387224	-1.945969157
C	-2.135816060	-2.934115462	-3.156762542
N	-1.940929169	-4.140313406	-2.366099363
H	-2.782442026	-4.697473036	-2.163943145
H	2.585424870	-4.318180493	-2.638385279
H	1.763712229	-5.511074044	-1.602202616
H	1.448320741	-5.483890528	-3.337877147
H	-1.182176031	-1.658873109	-5.365726882
H	-0.214304000	-0.430466053	-4.540909303
H	-1.967055326	-0.476568438	-4.306877274
C	0.411068837	-0.868541749	0.283530958
H	1.299218813	-0.405075710	-0.155897977
H	0.699169465	-1.358479231	1.216425357
H	-0.307837012	-0.078474357	0.518323283
C	-1.362942700	-2.528564285	0.001205951
O	-2.626660490	-2.154907817	-0.327208014
N	-1.323198324	-3.500167887	0.909216178
C	-4.608148937	-5.051616442	1.862850691
C	-3.194842741	-4.867149723	1.967351412
C	-5.357507083	-4.223977711	1.040624772
H	-2.603570321	-5.544698414	2.564758417
C	-2.607006456	-3.819851647	1.141382787
C	-4.801482244	-3.129876076	0.268030025
H	-5.435798049	-2.549240804	-0.384846360
C	-3.433046994	-3.004389080	0.362547842
C	-7.470900802	-7.242198280	2.972475202
C	-8.829242892	-7.340956382	2.734619262
C	-6.713173840	-6.240029556	2.358586719
H	-9.410914223	-8.120408997	3.215000923
C	-9.458734047	-6.434083899	1.872503374
C	-7.362425945	-5.353098459	1.503611952
H	-10.525077482	-6.510313212	1.687298949
C	-8.728048588	-5.434492881	1.252856708
H	-9.185075618	-4.719499408	0.578748457
H	-6.947435861	-7.924744684	3.632634874
C	-5.261731986	-6.131806020	2.589134341
O	-6.679389682	-4.351110645	0.872394688
O	-4.658879254	-6.908152569	3.325964548
H	-2.419781180	-3.228416534	-4.174892049
H	-2.977097291	-2.377550064	-2.735150018
O	-0.718329658	-5.710186453	-1.309446035
H	-4.458931410	-10.487576112	2.070344638
C	-3.623432551	-9.875788919	1.744345943
C	-2.359369572	-10.088199503	2.252864294
C	-3.855902314	-8.858056841	0.808799019
C	-1.291568367	-9.284596625	1.832524415
H	-2.192135357	-10.873101112	2.982299139
C	-1.491357159	-8.274371709	0.913910830
H	-0.298361048	-9.452421797	2.235610101
C	-2.777477655	-8.059289307	0.406776747
H	-0.680924631	-7.635180772	0.578752166
N	-3.006075072	-7.035554110	-0.490395432
C	-5.150460602	-8.579433635	0.249920789

C -5.336112748 -7.547604569 -0.606374428
H -5.981589996 -9.202649529 0.556492217
C -4.212476030 -6.680049050 -1.002069978
O -6.504797484 -7.181158712 -1.174522690
O -4.342229239 -5.694051122 -1.740489013
H -2.190413909 -6.481868813 -0.802229309
C -7.624544806 -8.023505895 -0.953818730
C -8.780434502 -7.482497981 -1.777050030
H -7.884575333 -8.036228419 0.112107406
H -7.386770077 -9.051629998 -1.258332928
C -10.033374951 -8.266066018 -1.520705055
H -8.923981890 -6.431957717 -1.501633151
H -8.513262579 -7.509662710 -2.837218561
C -10.661886820 -9.008686931 -2.422939408
H -10.419865933 -8.220034238 -0.502701242
H -10.300905434 -9.083368353 -3.445252894
H -11.560189697 -9.566441609 -2.179629137

==============================
anti-7
==============================

C 0.565632532 -3.865918906 -2.308624066
C 0.355941013 -2.902921584 -3.480591356
C -0.868748403 -2.023287357 -3.229764774
C -0.653306255 -1.225057777 -1.924800097
C -0.215065747 -2.004860449 -0.667536895
C 0.861491230 -3.047382491 -1.033054737
H 0.226055984 -3.466254106 -4.412754774
H 1.251460411 -2.281592052 -3.605108022
H -1.544012169 -0.630120725 -1.701707625
H 0.150850930 -0.509838129 -2.131922327
H 1.011874820 -3.721994847 -0.187112751
H 1.803273017 -2.512404866 -1.203726331
C 1.734452043 -4.811923903 -2.577611957
C -1.063659878 -1.020592219 -4.369136730
C -0.697739831 -4.703082895 -2.086038199
C -2.093678552 -2.948782159 -3.207594905
N -1.876985967 -4.186483545 -2.475209586
H -2.703059333 -4.783031981 -2.339930706
H 2.659395270 -4.240253219 -2.697720231
H 1.850819683 -5.516368335 -1.753741889
H 1.567057932 -5.390254110 -3.490276892
H -1.148482978 -1.531823780 -5.332404003
H -0.218574159 -0.328802778 -4.430368761
H -1.971922855 -0.427721592 -4.219944134
C 0.356544019 -1.018369265 0.368067277
H 1.223032770 -0.494728983 -0.045061618
H 0.665159988 -1.551270209 1.269913143
H -0.392322093 -0.272183663 0.649502371
C -1.363490996 -2.704873929 0.005086722
O -2.637632415 -2.304753377 -0.327923786
N -1.296938744 -3.647329398 0.874330622
C -4.589022153 -5.109868862 1.873392073
C -3.197770456 -4.967934123 1.914468099
C -5.368291387 -4.267273147 1.056351225
H -2.617891081 -5.659848408 2.512626539
C -2.624932264 -3.981038033 1.137395397
C -4.813651903 -3.250684550 0.288335573
H -5.430878268 -2.632736122 -0.349444728
C -3.444753534 -3.150449395 0.363755023
C -7.415111756 -7.352883890 3.011535327
C -8.771983609 -7.482979177 2.789322043
C -6.688858802 -6.320276071 2.409322728
H -9.330221055 -8.285986794 3.257660881
C -9.426851130 -6.564886092 1.958524736
C -7.355970506 -5.424028495 1.576242906
H -10.494564201 -6.656725188 1.785931693
C -8.728458111 -5.537693242 1.351062105
H -9.213343199 -4.818253998 0.701650857
H -6.867762251 -8.034750930 3.653151248
C -5.238883431 -6.182024738 2.648415704
O -6.723145512 -4.399074398 0.951596697
O -4.632250182 -6.910515407 3.417702050
H -2.359786530 -3.197950347 -4.242733709
H -2.952283838 -2.433391625 -2.768405557
O -0.627140517 -5.804840610 -1.530611714
H -4.553645110 -10.135086652 2.342126171
C -3.704479202 -9.591294134 1.941810884
C -2.454869717 -9.740222149 2.485409004
C -3.933885110 -8.699184349 0.845958313
C -1.367812064 -9.011513020 1.974891213
H -2.305940857 -10.415366805 3.321251620
C -1.546341201 -8.127660589 0.908216134
H -0.382663988 -9.128535539 2.412427056
C -2.798666635 -7.967859523 0.340639078
H -0.717371700 -7.553270540 0.507575374
N -2.978294876 -7.098456128 -0.718239914
C -5.177137487 -8.499435955 0.280456271
C -5.333815217 -7.481569483 -0.768285075
H -6.037964537 -9.032822615 0.656728111
C -4.206261396 -6.735134256 -1.246085985
O -6.523895224 -7.135105135 -1.272885198
O -4.277161365 -5.813263060 -2.078321579
H -2.152068165 -6.600664317 -1.073554744
C -7.652531975 -7.972277285 -1.016768927
C -8.816667672 -7.424550395 -1.822185504
H -7.890018948 -7.964330228 0.051681944
H -7.427336452 -9.000957899 -1.318553389
C -10.065604703 -8.209564345 -1.548861526
H -8.954209225 -6.374433575 -1.541464820
H -8.564595620 -7.449668961 -2.885979110
C -10.701486854 -8.955655087 -2.442909799
H -10.441121098 -8.162644067 -0.526915532
H -10.351621662 -9.031326446 -3.468956914
H -11.595620267 -9.515097852 -2.188672206

==============================
syn-6 (conformer2/12)
==============================
C 0.295270425 -4.017873351 -2.513719054
C 0.234337876 -3.034173222 -3.683336392
C -0.841944875 -1.979185992 -3.421650798
C -0.539890345 -1.246396375 -2.092660445
C -0.146278811 -2.081811057 -0.855253208
C 0.770031550 -3.257319972 -1.261266707
H 0.009760607 -3.565794388 -4.615764502
H 1.216284448 -2.562663050 -3.811871409
H -1.378291782 -0.591747984 -1.835701293
H 0.315925188 -0.592117602 -2.293212885
H 0.874343383 -3.950278690 -0.422869149
H 1.767261668 -2.856371299 -1.476372594
C 1.259429767 -5.166927902 -2.805826536
C -0.861603093 -0.930935297 -4.537156689
C -1.092857725 -4.609337121 -2.245089831
C -2.190724902 -2.708413496 -3.439692617
N -2.153133613 -4.008827815 -2.790632808
H -3.072374387 -4.439139811 -2.614041235
H 2.272929711 -4.780980016 -2.949623986
H 1.260683045 -5.881387036 -1.981862185
H 0.965830553 -5.699780912 -3.714499050
H -1.007120936 -1.401418339 -5.513647030
H 0.080126984 -0.375788987 -4.568517698
H -1.672434714 -0.211843849 -4.383987417
C 0.605053697 -1.173682233 0.141648202
H 1.527320968 -0.797894390 -0.309868594
H 0.862984920 -1.724505533 1.049039269
H -0.013331589 -0.316615512 0.422397091
C -1.342992100 -2.611886779 -0.131917455
O -1.102625811 -3.462780078 0.903134146
N -2.638034446 -2.390943314 -0.334965206
C -5.052543489 -4.286658663 1.825008457
C -4.694606098 -3.390013427 0.779387086
C -4.058320319 -4.896385966 2.585551779
H -5.454557337 -2.972181530 0.136026725
C -3.274441574 -3.157677455 0.567689673
C -2.639978285 -4.692995924 2.377825136
H -1.921637391 -5.234083650 2.974566258
C -2.329149136 -3.828373558 1.354800860
C -7.965785230 -6.003816990 3.494794145
C -8.158554453 -6.915366577 4.514794298
C -6.676670088 -5.577088116 3.156793461
H -9.161489206 -7.240863074 4.769578186
C -7.061970056 -7.422338176 5.224174745
C -5.604635329 -6.100312589 3.877289951
H -7.216764647 -8.139736715 6.022611520
C -5.778025413 -7.016310154 4.907811524
H -4.905493621 -7.399737555 5.423045385
H -8.791719215 -5.590692067 2.926729718
C -6.447264360 -4.632252058 2.055570522
O -4.316783380 -5.747715501 3.580738423
O -7.371152652 -4.189674605 1.366750117
H -2.503515519 -2.852564755 -4.480710395
H -2.957179171 -2.107890849 -2.941893994
O -1.208730483 -5.622387579 -1.532797242
C -5.140634870 -8.677268807 1.791336112
H -6.109395983 -8.997684496 2.162724473
C -3.978952939 -9.128385546 2.385559842
C -5.094111254 -7.781897629 0.712690255
C -2.735064466 -8.695482347 1.910269912
H -4.031092784 -9.816104028 3.222749582
C -3.837456040 -7.359186552 0.248608053
C -6.270543883 -7.253263172 0.078045475
C -2.657596299 -7.816277862 0.847813243
H -1.823336478 -9.051736387 2.378061128
H -1.705877981 -7.464430914 0.462544536
N -3.782777244 -6.469189584 -0.802487702
C -4.859763391 -5.913934582 -1.428518536
H -2.850158533 -6.168377312 -1.134547246
C -6.177240095 -6.355262779 -0.931452554
H -7.235726671 -7.566825863 0.455655187
O -7.193156625 -5.766630556 -1.576609966
O -4.727841517 -5.094349948 -2.341052480
C -8.506688343 -6.014048284 -1.092670868
C -9.466042718 -5.209370218 -1.952103459
H -8.566105787 -5.705256241 -0.042447786
H -8.739032656 -7.085244474 -1.167204134
C -10.878879865 -5.372381564 -1.475364220
H -9.161070180 -4.158769017 -1.898796184
H -9.370571819 -5.528553719 -2.994195766
C -11.843034778 -5.974850219 -2.159933640
H -11.095097220 -4.976282484 -0.483593273
H -11.662202337 -6.384198316 -3.150371189
H -12.847991203 -6.077540151 -1.763834045

==============================
syn-6 (conformer3/12)
==============================
C 0.290108322 -4.008955787 -2.520155110
C 0.225565233 -3.021326684 -3.686232969
C -0.850306445 -1.967536906 -3.418155188
C -0.544719405 -1.238692384 -2.087711318
C -0.147335721 -2.078060959 -0.854098222
C 0.767904657 -3.252376300 -1.266554374
H -0.001336712 -3.549836142 -4.619868373
H 1.207068277 -2.549161518 -3.815896111
H -1.382646495 -0.585167597 -1.826327999
H 0.310295281 -0.583497744 -2.288720372
H 0.874473853 -3.948028891 -0.430672733
H 1.764531855 -2.850528843 -1.482793710
C 1.253917361 -5.156602680 -2.818922165
C -0.873706126 -0.915711810 -4.530256123
C -1.097022046 -4.601676164 -2.249171277

C -2.198836392 -2.697210635 -3.434533897
N -2.159223896 -3.998910060 -2.788323087
H -3.077777044 -4.428727388 -2.606917956
H 2.266868077 -4.769817960 -2.964339681
H 1.257767777 -5.873952727 -1.997478609
H 0.957779246 -5.686320266 -3.728587355
H -1.022122533 -1.383089196 -5.507797895
H 0.067741909 -0.360151057 -4.562744117
H -1.684320570 -0.197435565 -4.372201294
C 0.607173807 -1.173206265 0.143479639
H 1.527861472 -0.795667515 -0.309784233
H 0.868193473 -1.727161481 1.048076191
H -0.010446029 -0.317244703 0.429234310
C -1.342079946 -2.610189663 -0.129051532
O -1.099106829 -3.464457785 0.902577419
N -2.637876966 -2.389171037 -0.328265945
C -5.046348054 -4.294898556 1.829843531
C -4.691379583 -3.393215838 0.787198426
C -4.050181562 -4.906685480 2.585975306
H -5.452969137 -2.973897536 0.146739003
C -3.271726581 -3.159335197 0.573073330
C -2.632372556 -4.701295564 2.375960274
H -1.912302032 -5.244649505 2.968577891
C -2.324366430 -3.832304252 1.355676266
C -7.954807114 -6.020280589 3.499370216
C -8.144877806 -6.935996110 4.516165366
C -6.666691353 -5.591571132 3.160311916
H -9.147049472 -7.263124680 4.771864249
C -7.046434925 -7.445005941 5.221085803
C -5.592755391 -6.116876575 3.876248814
H -7.198999666 -8.165666138 6.017028682
C -5.763365975 -7.036998057 4.903561647
H -4.889550710 -7.421960587 5.415456782
H -8.782148891 -5.605277553 2.934731858
C -6.440375576 -4.641863607 2.062409751
O -4.305723842 -5.762470471 3.578108060
O -7.366076402 -4.196622207 1.377970164
H -2.513956245 -2.839186201 -4.475168615
H -2.964403044 -2.098051810 -2.933748827
O -1.210531480 -5.617106428 -1.539956155
C -5.140629540 -8.690419517 1.778589155
H -6.109212153 -9.011753176 2.149676035
C -3.978580671 -9.145308372 2.369162891
C -5.094785476 -7.789960139 0.704166512
C -2.735026564 -8.711181339 1.894252496
H -4.030141429 -9.837019978 3.203105157
C -3.838435146 -7.366040624 0.240406333
C -6.271709968 -7.257160308 0.073908549
C -2.658269677 -7.826964594 0.835811793
H -1.822941442 -9.070402511 2.359082936
H -1.706658750 -7.474191494 0.451057693
N -3.784415725 -6.470742609 -0.806431642
C -4.861896863 -5.910919480 -1.427551945
H -2.852048903 -6.169403110 -1.138111461
C -6.178971700 -6.354073114 -0.931220686
H -7.236622474 -7.571885012 0.451246587
O -7.195236691 -5.761258847 -1.572039739
O -4.730979421 -5.085893014 -2.335340094
C -8.508428570 -6.008760531 -1.087225684
C -9.467506165 -5.197450905 -1.940733240
H -8.565506604 -5.705552491 -0.035262447
H -8.743156439 -7.079035691 -1.167230033
C -10.879880840 -5.359898226 -1.462502211
H -9.160116713 -4.147843004 -1.882314521
H -9.374455078 -5.511273446 -2.984667393
C -11.846022357 -5.958173765 -2.147951013
H -11.093939235 -4.967292821 -0.468899010
H -11.667334608 -6.363845431 -3.140306790
H -12.850559163 -6.060804708 -1.750744855

==============================
syn-6 (conformer4/12)
==============================

C 0.288721007 -4.001093444 -2.518236583
C 0.225038436 -3.006587133 -3.678489592
C -0.848422177 -1.952309120 -3.403082277
C -0.540419160 -1.231952989 -2.068506311
C -0.143300219 -2.079461060 -0.840329714
C 0.769577547 -3.252575727 -1.260967463
H -0.003991517 -3.529070290 -4.614977356
H 1.207375905 -2.535716480 -3.806410937
H -1.377026090 -0.578657075 -1.802421688
H 0.315514519 -0.576970600 -2.266267598
H 0.876527929 -3.953262030 -0.429372744
H 1.766400622 -2.850853890 -1.476546711
C 1.249941681 -5.148912680 -2.824650614
C -0.870694788 -0.893684137 -4.508788149
C -1.099393078 -4.592950709 -2.249830570
C -2.198403003 -2.679070693 -3.422645628
N -2.160730575 -3.985098092 -2.785035970
H -3.080087132 -4.415183561 -2.608270625
H 2.263563606 -4.763350057 -2.968627632
H 1.253008692 -5.871269218 -2.007587256
H 0.951911174 -5.672488910 -3.737237111
H -1.020777176 -1.354842539 -5.489029195
H 0.071755079 -0.339677074 -4.538579645
H -1.679852006 -0.174876066 -4.345705941
C 0.613339412 -1.181831840 0.162024676
H 1.533959904 -0.802593634 -0.289972551
H 0.874713443 -1.741676685 1.062900139
H -0.002981793 -0.326942210 0.453679301
C -1.338151883 -2.614500102 -0.117616338
O -1.095462835 -3.476514891 0.907520788
N -2.633585893 -2.389139559 -0.313506273
C -5.043135410 -4.306413120 1.833609714
C -4.687766228 -3.397725592 0.797091148

C -4.047250005 -4.925573322 2.583843184
H -5.449503034 -2.971902636 0.161134168
C -3.267917219 -3.164509549 0.583122614
C -2.629324993 -4.721433645 2.373407817
H -1.909484839 -5.270455311 2.961056422
C -2.320964462 -3.845087106 1.359482783
C -7.952874586 -6.037038357 3.495290985
C -8.143529228 -6.958995774 4.506340055
C -6.664291526 -5.609028403 3.157027179
H -9.146098452 -7.285329276 4.761485590
C -7.045216756 -7.475289903 5.206147274
C -5.590490572 -6.141506179 3.867957740
H -7.198223116 -8.200913991 5.997471121
C -5.761683486 -7.068151553 4.889310530
H -4.887988380 -7.458598595 5.397274896
H -8.780186224 -5.616413802 2.934769044
C -6.437537475 -4.652536968 2.065261106
O -4.303130082 -5.787881763 3.570297345
O -7.363174187 -4.201148031 1.384853711
H -2.515159525 -2.813666623 -4.463754755
H -2.962086555 -2.081625124 -2.916976122
O -1.214217338 -5.612818563 -1.547180355
C -5.144211120 -8.703019244 1.753849129
H -6.112888457 -9.024954052 2.124177931
C -3.982150393 -9.164973621 2.338906864
C -5.098237421 -7.794620600 0.686117170
C -2.738454258 -8.730001934 1.865058998
H -4.033778636 -9.862879995 3.167663778
C -3.841778494 -7.370022729 0.223318813
C -6.275222059 -7.254772955 0.061858852
C -2.661598782 -7.838110580 0.813118872
H -1.826340317 -9.094765891 2.325492156
H -1.709861855 -7.484716692 0.429284708
N -3.787755482 -6.467314408 -0.817156687
C -4.865156300 -5.900876602 -1.432265526
H -2.855610004 -6.164683012 -1.148588341
C -6.182364218 -6.344541516 -0.936644410
H -7.240074120 -7.570458954 0.438608617
O -7.198468363 -5.745219409 -1.571628085
O -4.734078637 -5.069995288 -2.334658812
C -8.511352191 -5.995351502 -1.087532945
C -9.470626879 -5.176349277 -1.933463870
H -8.567212111 -5.700424309 -0.033191373
H -8.747018825 -7.064794926 -1.175693159
C -10.882619321 -5.341420960 -1.454933211
H -9.162242433 -4.127516847 -1.866776872
H -9.378927846 -5.481812619 -2.979984752
C -11.850298861 -5.932872061 -2.144129188
H -11.095120309 -4.957015307 -0.457771289
H -11.673459429 -6.330322360 -3.140121777
H -12.854403582 -6.037758657 -1.746448197

===============================

syn-6 (conformer5/12)
===============================
C 0.281716260 -4.027681386 -2.541589150
C 0.215684186 -3.039984511 -3.707499559
C -0.854238461 -1.981509584 -3.434346190
C -0.538714233 -1.253674925 -2.105642711
C -0.138826629 -2.094446569 -0.873714354
C 0.768912894 -3.273094219 -1.290459117
H -0.018222700 -3.567703756 -4.639838114
H 1.198578701 -2.572150984 -3.842239613
H -1.372324496 -0.596269710 -1.840209934
H 0.318225596 -0.602390183 -2.311189800
H 0.876362075 -3.969121549 -0.454995046
H 1.766277287 -2.875815323 -1.511675302
C 1.239086231 -5.179507657 -2.844970950
C -0.878591413 -0.929948883 -4.546673352
C -1.106526792 -4.614501244 -2.263899299
C -2.206018051 -2.705309111 -3.443825510
N -2.168810678 -4.007081839 -2.797643954
H -3.088127947 -4.434106855 -2.613733601
H 2.252978014 -4.797101022 -2.995301017
H 1.243825257 -5.896941819 -2.023607117
H 0.936153847 -5.707820838 -3.753194525
H -1.033807662 -1.397020469 -5.523314097
H 0.065080598 -0.378473176 -4.583962861
H -1.685308851 -0.208099071 -4.384970263
C 0.624754018 -1.192685449 0.119716827
H 1.544938658 -0.819562123 -0.338175047
H 0.887636011 -1.747419859 1.023294443
H 0.012363422 -0.333842908 0.408006362
C -1.332377522 -2.620616083 -0.142227468
O -1.088039397 -3.474950958 0.889060742
N -2.627892301 -2.393970303 -0.335321111
C -5.034474555 -4.287183632 1.835953895
C -4.680498480 -3.387785052 0.791027340
C -4.037427089 -4.902574963 2.588047303
H -5.443287952 -2.964813350 0.154422153
C -3.260858498 -3.160298212 0.570123389
C -2.619763435 -4.703444607 2.371248947
H -1.899221489 -5.249500632 2.960812904
C -2.312777169 -3.836691756 1.348613426
C -7.942496102 -5.998535532 3.520421905
C -8.131802113 -6.912311520 4.539143690
C -6.654148925 -5.575687630 3.174998873
H -9.134195128 -7.234905375 4.799713874
C -7.032380239 -7.425165324 5.239699380
C -5.579261204 -6.104733812 3.886733658
H -7.184373120 -8.144251739 6.037169952
C -5.749035613 -7.022938921 4.915862899
H -4.874497696 -7.411039010 5.424148996
H -8.770670755 -5.580676363 2.959109074
C -6.428805269 -4.628067879 2.075099436
O -4.292017376 -5.756233257 3.582219111

O	-7.355748617	-4.179613625	1.394435389
H	-2.527012620	-2.846078071	-4.482828685
H	-2.966439005	-2.102746977	-2.939314620
O	-1.220775202	-5.629772376	-1.554570351
C	-5.142513634	-8.677384459	1.791875973
H	-6.110025855	-8.993330459	2.170296980
C	-3.978615582	-9.136472013	2.375523358
C	-5.099765401	-7.779743819	0.714978881
C	-2.736278002	-8.709331393	1.891141787
H	-4.027721728	-9.826001359	3.211426617
C	-3.844616282	-7.362974008	0.241580359
C	-6.278408109	-7.243151451	0.091077631
C	-2.662557839	-7.827995282	0.830125325
H	-1.822757523	-9.071783330	2.350630659
H	-1.711971260	-7.480489914	0.438111624
N	-3.793434850	-6.470712019	-0.807877263
C	-4.872383253	-5.907692237	-1.423583438
H	-2.861849403	-6.173447941	-1.145627889
C	-6.188199010	-6.343773741	-0.917599370
H	-7.242232941	-7.552230492	0.475774466
O	-7.205956931	-5.749104026	-1.554269686
O	-4.743485865	-5.085589660	-2.334271158
C	-8.517364296	-5.992136438	-1.062634979
C	-9.478758732	-5.183186689	-1.915790767
H	-8.569446864	-5.684186794	-0.011779529
H	-8.754193590	-7.062381910	-1.136775000
C	-10.889638522	-5.342779102	-1.432301060
H	-9.170398873	-4.133585204	-1.862433407
H	-9.389374623	-5.500990220	-2.958850689
C	-11.858958363	-5.941409240	-2.112929697
H	-11.099771601	-4.947547142	-0.438892852
H	-11.684356489	-6.349715224	-3.104938462
H	-12.862217597	-6.041744436	-1.711916638

==============================
syn-6 (conformer6/12)
==============================

C	0.273192971	-3.994034796	-2.540213165
C	0.205845467	-2.975706232	-3.679420544
C	-0.852950898	-1.916344429	-3.370201614
C	-0.521254641	-1.224425754	-2.025941765
C	-0.118199655	-2.100386156	-0.819444851
C	0.776226348	-3.275428722	-1.274767108
H	-0.040069520	-3.476643024	-4.623321709
H	1.191644427	-2.512869786	-3.810153084
H	-1.347776034	-0.568063246	-1.736896908
H	0.339014410	-0.574473894	-2.221776496
H	0.885603618	-3.993417293	-0.458408925
H	1.774613288	-2.879682783	-1.494222932
C	1.219330568	-5.144660507	-2.881357568
C	-0.877925973	-0.836193309	-4.455054506
C	-1.117476948	-4.577838865	-2.267368002
C	-2.210491481	-2.628707915	-3.386988012
N	-2.179041964	-3.946363298	-2.773692039
H	-3.100091916	-4.371488899	-2.593494639
H	2.234959254	-4.766230819	-3.029951727
H	1.224824275	-5.883768848	-2.079397423
H	0.904738080	-5.646284003	-3.800619931
H	-1.044683588	-1.276775749	-5.442057112
H	0.069665298	-0.291085343	-4.485694716
H	-1.677757617	-0.112722643	-4.268147001
C	0.661336817	-1.231415776	0.190514591
H	1.579598568	-0.852444045	-0.266433019
H	0.929038317	-1.812246312	1.076150210
H	0.057848710	-0.376521627	0.507660259
C	-1.310819978	-2.635337047	-0.093217491
O	-1.067634129	-3.522488588	0.910206399
N	-2.605845983	-2.391769303	-0.270868764
C	-5.014921813	-4.331953602	1.855598593
C	-4.659965900	-3.403494923	0.836664428
C	-4.018673234	-4.977532753	2.582665006
H	-5.422957423	-2.957816342	0.215987531
C	-3.239674785	-3.180025880	0.614372608
C	-2.600599874	-4.783518586	2.362998542
H	-1.880910914	-5.352889653	2.931190463
C	-2.292639550	-3.887965481	1.365944386
C	-7.927269570	-6.067553769	3.507121268
C	-8.118771113	-7.006966480	4.501783306
C	-6.637319753	-5.646457397	3.165627086
H	-9.122422297	-7.327924462	4.759505121
C	-7.019799409	-7.547814074	5.181667923
C	-5.562806814	-6.203844535	3.856060841
H	-7.173354175	-8.286793550	5.960441684
C	-5.734826848	-7.148104873	4.861003046
H	-4.861019202	-7.557299693	5.353829686
H	-8.754995027	-5.627241811	2.962525812
C	-6.410500992	-4.669927359	2.091894407
O	-4.274432070	-5.858628781	3.552571937
O	-7.337496826	-4.195565601	1.429253686
H	-2.540474086	-2.741158507	-4.426648152
H	-2.962167287	-2.032813641	-2.861949466
O	-1.234330898	-5.612374645	-1.586856760
C	-5.167126260	-8.730751331	1.695685048
H	-6.135935573	-9.044881641	2.072326839
C	-4.005009262	-9.224527362	2.254034887
C	-5.120741480	-7.800759213	0.646705871
C	-2.760992896	-8.799333245	1.772298929
H	-4.056662752	-9.939634080	3.068026251
C	-3.864094063	-7.386098438	0.175799099
C	-6.297511521	-7.230406616	0.049321325
C	-2.683895679	-7.885922860	0.738961648
H	-1.848739373	-9.188771750	2.211833886
H	-1.731590168	-7.539529492	0.350102597
N	-3.809431583	-6.462572333	-0.846517066
C	-4.886023547	-5.866388912	-1.434297414

H	-2.877089490	-6.164565622	-1.181320228
C	-6.203503472	-6.303005577	-0.933012442
H	-7.262002329	-7.539279608	0.432537117
O	-7.218096269	-5.679542528	-1.546619227
O	-4.754805140	-5.016795915	-2.319090284
C	-8.530531786	-5.932160197	-1.063030494
C	-9.488826259	-5.098045082	-1.895177294
H	-8.582814548	-5.652712436	-0.004329114
H	-8.769982789	-6.999404398	-1.166320025
C	-10.900823780	-5.267007252	-1.418117356
H	-9.177969083	-4.051007590	-1.813218042
H	-9.398790941	-5.387942877	-2.946234874
C	-11.871260662	-5.842559682	-2.116811692
H	-11.111073662	-4.899991916	-0.413951770
H	-11.696957470	-6.222567720	-3.120050614
H	-12.875323143	-5.951070900	-1.719985930

==============================

syn-6 (conformer7/12)

==============================

C	0.292721728	-4.005125755	-2.499751722
C	0.220874434	-3.041704056	-3.685728317
C	-0.857016053	-1.985222374	-3.436299062
C	-0.545456774	-1.225187964	-2.125451638
C	-0.150372695	-2.037816426	-0.874432063
C	0.765487991	-3.220039578	-1.260923435
H	-0.007629544	-3.590751244	-4.607069031
H	1.200377067	-2.569127513	-3.828930243
H	-1.380440568	-0.562826474	-1.877566118
H	0.311929306	-0.578335429	-2.343257686
H	0.873356525	-3.897731031	-0.410701215
H	1.761231592	-2.820289822	-1.484898754
C	1.265755288	-5.150610198	-2.774992667
C	-0.889697433	-0.960240836	-4.572593363
C	-1.090367277	-4.603027146	-2.217525189
C	-2.203791054	-2.719426942	-3.427877765
N	-2.157536605	-4.007755334	-2.755801699
H	-3.074247917	-4.435102385	-2.559038460
H	2.275902784	-4.759040366	-2.927162771
H	1.274136269	-5.851349454	-1.939426337
H	0.974832150	-5.701060297	-3.674029173
H	-1.041428241	-1.450995317	-5.538119784
H	0.049663269	-0.402401359	-4.623235501
H	-1.701957102	-0.241082073	-4.427553218
C	0.599126987	-1.114218564	0.108619100
H	1.521868577	-0.745386298	-0.347493373
H	0.856332633	-1.650780863	1.024783251
H	-0.019805766	-0.253075336	0.375342925
C	-1.349298017	-2.553925866	-0.142412310
O	-1.105143464	-3.410867904	0.889821457
N	-2.631013941	-2.318166753	-0.340227273
C	-5.040744479	-4.241891330	1.787496797
C	-4.679503574	-3.321695811	0.772757542
C	-4.047161589	-4.864866168	2.548691329
H	-5.441980125	-2.879697386	0.147900633
C	-3.277996474	-3.089877931	0.567243566
C	-2.643488173	-4.653439765	2.352975160
H	-1.921709816	-5.204141625	2.937274749
C	-2.332326472	-3.770928288	1.344599984
C	-7.970300239	-5.978832194	3.414141353
C	-8.172394457	-6.904005819	4.419641286
C	-6.677172042	-5.555174116	3.086334402
H	-9.178092829	-7.226994372	4.666434560
C	-7.080814246	-7.427312036	5.125466376
C	-5.609288664	-6.093294057	3.803744252
H	-7.242504861	-8.155284161	5.912917816
C	-5.793665343	-7.023764498	4.820345090
H	-4.925493430	-7.419717009	5.333309990
H	-8.792317540	-5.551546982	2.850798159
C	-6.439734068	-4.593300860	2.002380703
O	-4.319897293	-5.742761139	3.522537862
O	-7.358176158	-4.138224767	1.315859258
H	-2.525127037	-2.883537014	-4.463415513
H	-2.967499153	-2.111461494	-2.935048107
O	-1.196734080	-5.611936563	-1.498907022
C	-5.120646689	-8.755914101	1.804694438
H	-6.087507596	-9.077210854	2.179815326
C	-3.956483679	-9.211237949	2.386306775
C	-5.083501162	-7.845503407	0.729514844
C	-2.714675858	-8.773332152	1.907460833
H	-4.002607338	-9.906924142	3.217350321
C	-3.822171130	-7.418699381	0.260539007
C	-6.255308386	-7.313084977	0.119279944
C	-2.644042185	-7.882456129	0.848965334
H	-1.799946861	-9.133187647	2.366255955
H	-1.693392266	-7.526610951	0.464714380
N	-3.771709763	-6.512426106	-0.777542750
C	-4.857271480	-5.935501370	-1.378814926
H	-2.843192882	-6.198187985	-1.103592087
C	-6.162949026	-6.384735543	-0.891501124
H	-7.219848680	-7.630393447	0.494314945
O	-7.189068946	-5.791139389	-1.512111888
O	-4.723970507	-5.083007541	-2.264506790
C	-8.501415002	-6.045672955	-1.025386912
C	-9.461608120	-5.218728151	-1.862095329
H	-8.554688955	-5.755387712	0.029677072
H	-8.736654611	-7.114008702	-1.120761237
C	-10.872138031	-5.385476038	-1.379863382
H	-9.150549492	-4.171339797	-1.786786002
H	-9.373713711	-5.515810790	-2.911324465
C	-11.842967112	-5.970774658	-2.069817629
H	-11.080605052	-5.007349339	-0.379450562
H	-11.670241084	-6.361442058	-3.069209260
H	-12.845871149	-6.076822369	-1.669450600

==============================

syn-6 (conformer8/12)

==============================

C	0.297125170	-4.008639262	-2.492910522
C	0.227677370	-3.046495692	-3.680106917
C	-0.850246177	-1.989362488	-3.433648237
C	-0.540763429	-1.228256596	-2.122892390
C	-0.147857058	-2.039728279	-0.870416773
C	0.768406084	-3.222505924	-1.254276273
H	0.000510598	-3.596470487	-4.601223413
H	1.207575927	-2.574412545	-3.822145428
H	-1.376026175	-0.565496869	-1.877020219
H	0.317093913	-0.581751620	-2.339864430
H	0.874708467	-3.899341034	-0.403179286
H	1.764602967	-2.823271585	-1.477158446
C	1.269711748	-5.155213649	-2.765221847
C	-0.880260512	-0.965415400	-4.570968119
C	-1.086768672	-4.605253238	-2.212245617
C	-2.197400818	-2.722999168	-3.427246015
N	-2.152449748	-4.011177730	-2.754818586
H	-3.069208899	-4.439054217	-2.560127421
H	2.280387617	-4.764587553	-2.916295954
H	1.276250698	-5.854830154	-1.928692265
H	0.979806188	-5.706622560	-3.664001736
H	-1.030162724	-1.457036235	-5.536342323
H	0.059378828	-0.407926962	-4.620213299
H	-1.692567879	-0.245823317	-4.428329596
C	0.600279415	-1.115345077	0.112925039
H	1.523865016	-0.747230854	-0.342050561
H	0.855866028	-1.651010052	1.030066730
H	-0.018836668	-0.253752732	0.377775271
C	-1.347951656	-2.555158791	-0.139791083
O	-1.105381796	-3.410829929	0.893828660
N	-2.629469828	-2.320166440	-0.340165865
C	-5.042217795	-4.241369480	1.786695412
C	-4.679501300	-3.322421927	0.771162882
C	-4.049633260	-4.863552150	2.549689036
H	-5.441190983	-2.881096036	0.144865758
C	-3.277774775	-3.090937068	0.567216076
C	-2.645777112	-4.652145293	2.355848096
H	-1.924789945	-5.202237126	2.941706946
C	-2.333160760	-3.770871431	1.346858054
C	-7.973878736	-5.977220023	3.410655906
C	-8.177235342	-6.902115073	4.416069127
C	-6.680353575	-5.553431757	3.084545113
H	-9.183202935	-7.225202916	4.661627743
C	-7.086478283	-7.425113188	5.123544699
C	-5.613334331	-6.091245929	3.803502637
H	-7.249229516	-8.152926144	5.910925780
C	-5.799031162	-7.021489676	4.820122997
H	-4.931475556	-7.417071712	5.334414837
H	-8.795162612	-5.550210608	2.846035453
C	-6.441503071	-4.592131577	2.000421335
O	-4.323655658	-5.740651973	3.524042622
O	-7.359084321	-4.137586428	1.312367155
H	-2.517225624	-2.887177624	-4.463227031
H	-2.961603077	-2.114675684	-2.935618945
O	-1.195262955	-5.612839013	-1.492100297
C	-5.129168415	-8.757009411	1.800242155
H	-6.096987637	-9.078483999	2.172728129
C	-3.966456243	-9.213118347	2.384101635
C	-5.089307817	-7.845388813	0.726147161
C	-2.723427135	-8.775087740	1.908470048
H	-4.014616564	-9.909582298	3.214378262
C	-3.826821914	-7.418521574	0.260381391
C	-6.259572075	-7.311815749	0.114038040
C	-2.650133875	-7.883268225	0.850965866
H	-1.809858580	-9.135672735	2.369000172
H	-1.698500920	-7.527399701	0.469170011
N	-3.773632951	-6.511155995	-0.776710632
C	-4.857724586	-5.933353748	-1.379876392
H	-2.844039124	-6.197423271	-1.099999340
C	-6.164546185	-6.382235719	-0.895347573
H	-7.225052926	-7.628982885	0.486744179
O	-7.189192636	-5.787082857	-1.517105854
O	-4.722584885	-5.080217992	-2.264675377
C	-8.502399619	-6.040303359	-1.032331884
C	-9.460586620	-5.211907054	-1.869930081
H	-8.556908075	-5.750599351	0.022850942
H	-8.738859750	-7.108312602	-1.128556975
C	-10.871828085	-5.376964124	-1.389218104
H	-9.148214790	-4.164944249	-1.794142022
H	-9.371985911	-5.508959010	-2.919108641
C	-11.842384658	-5.961873182	-2.079892767
H	-11.081111655	-4.998032193	-0.389281361
H	-11.668869201	-6.353339836	-3.078835273
H	-12.845833231	-6.066799689	-1.680597162

==============================

syn-6 (conformer9/12)

==============================

C	0.297011292	-4.009748455	-2.492655153
C	0.228856954	-3.047416031	-3.679721100
C	-0.848515216	-1.989673120	-3.433544583
C	-0.539165491	-1.229057977	-2.122422554
C	-0.146769278	-2.040860041	-0.869991162
C	0.768740591	-3.224277479	-1.253818164
H	0.001814862	-3.597054103	-4.601065025
H	1.209132094	-2.575937049	-3.821145782
H	-1.374274537	-0.566083151	-1.876609435
H	0.318939036	-0.582788254	-2.339074077
H	0.874224739	-3.901364412	-0.402816703
H	1.765334949	-2.825827074	-1.476333124
C	1.268477890	-5.157310197	-2.764924460
C	-0.877237574	-0.965309640	-4.570561848
C	-1.087487507	-4.605090304	-2.212266940
C	-2.196118744	-2.722450815	-3.428184970

N -2.152422387 -4.011144941 -2.756498303
H -3.069726598 -4.437876010 -2.561766190
H 2.279542767 -4.767653566 -2.915894524
H 1.274261413 -5.856970196 -1.928428065
H 0.978092685 -5.708337326 -3.663781610
H -1.027046605 -1.456499714 -5.536165130
H 0.062855051 -0.408536991 -4.619164909
H -1.689056478 -0.245141755 -4.428053417
C 0.601975493 -1.116981998 0.113355436
H 1.525694459 -0.749361405 -0.341740342
H 0.857364327 -1.652846121 1.030425725
H -0.016600343 -0.255035473 0.378327173
C -1.347149342 -2.555631720 -0.139391707
O -1.104921091 -3.409916520 0.895454605
N -2.628618477 -2.321037453 -0.340572423
C -5.042015680 -4.240485358 1.786794751
C -4.679004147 -3.322349731 0.770973099
C -4.049605536 -4.861854245 2.551025068
H -5.440389034 -2.881496805 0.143970837
C -3.277225949 -3.090922257 0.567315643
C -2.645779098 -4.650064403 2.357944201
H -1.924948275 -5.198239616 2.945778168
C -2.332828138 -3.769766686 1.348237422
C -7.974005779 -5.976620059 3.410001176
C -8.177598407 -6.900728003 4.416193054
C -6.680526135 -5.552316192 3.084496406
H -9.183610201 -7.224139536 4.661151516
C -7.087197248 -7.422421924 5.125006274
C -5.613833129 -6.088942414 3.804895477
H -7.250072873 -8.149573750 5.912967864
C -5.799671833 -7.018279739 4.822160692
H -4.932298395 -7.412924450 5.337475462
H -8.795152106 -5.550812082 2.844284620
C -6.441292346 -4.592221443 1.999452333
O -4.324019210 -5.737977261 3.525873244
O -7.358416078 -4.139462655 1.309540267
H -2.515487230 -2.885833805 -4.464421828
H -2.960174164 -2.113928910 -2.936576978
O -1.196892561 -5.611754910 -1.490934382
C -5.130048710 -8.755601917 1.800953172
H -6.097858844 -9.078392994 2.172326206
C -3.967391592 -9.209477282 2.386824909
C -5.090116187 -7.844642538 0.726363622
C -2.724441632 -8.769861832 1.912668514
H -4.015729648 -9.905429175 3.217515544
C -3.827608149 -7.415991974 0.262193546
C -6.260354119 -7.313356585 0.112201092
C -2.651048002 -7.878551075 0.854654938
H -1.810928290 -9.128653803 2.374704557
H -1.699449239 -7.521562565 0.473841768
N -3.774601255 -6.509191339 -0.775291784
C -4.858736797 -5.932664816 -1.379614373
H -2.845414706 -6.193082986 -1.097479801
C -6.165548413 -6.383862974 -0.897270964
H -7.225846602 -7.632420556 0.483276907
O -7.190323637 -5.791050898 -1.520942375
O -4.723303614 -5.078987703 -2.263822581
C -8.503599889 -6.043891587 -1.035835738
C -9.461650528 -5.215217458 -1.873307763
H -8.557674807 -5.754077702 0.019311156
H -8.740385153 -7.111819697 -1.132086951
C -10.872977386 -5.380576730 -1.392940193
H -9.149380221 -4.168265329 -1.797003618
H -9.372794978 -5.511840760 -2.922587800
C -11.843388114 -5.965218426 -2.084043039
H -11.082443845 -5.002109601 -0.392865296
H -11.669699837 -6.356210239 -3.083141891
H -12.846895939 -6.070375920 -1.684954363

==============================
syn-6 (conformer10/12)
==============================

C 0.297062160 -4.010117751 -2.493034180
C 0.228040263 -3.047803232 -3.680116173
C -0.849387715 -1.990157926 -3.433568030
C -0.539577261 -1.229272939 -2.122757487
C -0.147001507 -2.041055606 -0.870361320
C 0.768671642 -3.224264548 -1.254311735
H 0.000633274 -3.597552149 -4.601309117
H 1.208154592 -2.576147912 -3.822099379
H -1.374580550 -0.566193420 -1.876824034
H 0.318538230 -0.583096885 -2.339689742
H 0.874641205 -3.901207693 -0.403255205
H 1.765080018 -2.825503226 -1.477123048
C 1.269229550 -5.157022769 -2.765453804
C -0.878892160 -0.966105803 -4.570832093
C -1.087093617 -4.606236261 -2.212606990
C -2.196899711 -2.723106520 -3.427122907
N -2.152523748 -4.011439455 -2.754915824
H -3.069455174 -4.438672484 -2.559620398
H 2.280064945 -4.766757293 -2.916378988
H 1.275427589 -5.856790260 -1.929050043
H 0.979181113 -5.708167811 -3.664347937
H -1.029079799 -1.457587983 -5.536228656
H 0.061052806 -0.409136759 -4.620058925
H -1.690797845 -0.246075907 -4.428121002
C 0.601662225 -1.117091797 0.113027457
H 1.525376609 -0.749366781 -0.342007120
H 0.857073370 -1.652965015 1.030095062
H -0.017015607 -0.255222771 0.378013333
C -1.347264374 -2.556015972 -0.139665211
O -1.104707055 -3.411689638 0.894024286
N -2.628725251 -2.320522320 -0.339641604
C -5.041536022 -4.241219878 1.787617680
C -4.678817333 -3.322338083 0.772137121
C -4.048915025 -4.863772476 2.550428163

H -5.440417661 -2.880734978 0.145922447
C -3.277058757 -3.091132457 0.567948462
C -2.645090736 -4.652546548 2.356341143
H -1.924026678 -5.202412272 2.942343655
C -2.332437313 -3.771302422 1.347321406
C -7.973135244 -5.976940350 3.411928718
C -8.176496917 -6.901831736 4.417451791
C -6.679607850 -5.553357300 3.085599235
H -9.182531620 -7.224698425 4.663101095
C -7.085779213 -7.424964403 5.124747268
C -5.612579865 -6.091324708 3.804462928
H -7.248447023 -8.152698913 5.912210417
C -5.798230079 -7.021474303 4.821057693
H -4.930660183 -7.417175583 5.335249848
H -8.794463844 -5.549843877 2.847440734
C -6.440802040 -4.592072808 2.001419663
O -4.322834620 -5.740848625 3.524678551
O -7.358500586 -4.137435761 1.313623570
H -2.516875610 -2.887004932 -4.463098657
H -2.960792212 -2.114489822 -2.935368083
O -1.196188299 -5.613868229 -1.492613158
C -5.131489547 -8.756416671 1.800328282
H -6.099485738 -9.077489529 2.172699627
C -3.969038822 -9.213045727 2.384430865
C -5.091071226 -7.844848519 0.726271531
C -2.725759362 -8.775559196 1.909003341
H -4.017696779 -9.909472859 3.214724458
C -3.828322449 -7.418452877 0.260752694
C -6.261048139 -7.310738361 0.113986012
C -2.651927298 -7.883739327 0.851494609
H -1.812402807 -9.136465419 2.369718076
H -1.700072312 -7.528245010 0.469871984
N -3.774646372 -6.511124560 -0.776318090
C -4.858426724 -5.932638198 -1.379400897
H -2.844920972 -6.197482161 -1.099407388
C -6.165537933 -6.381323310 -0.895409687
H -7.226700965 -7.627660924 0.486465396
O -7.189753745 -5.786080806 -1.517761686
O -4.722826728 -5.079254162 -2.263868590
C -8.503334242 -6.038531971 -1.033401796
C -9.460604477 -5.209555817 -1.871483541
H -8.558055754 -5.748679561 0.021712340
H -8.740340836 -7.106411686 -1.129703167
C -10.872309861 -5.374274612 -1.392072503
H -9.147996673 -4.162707298 -1.795045586
H -9.371182796 -5.506242597 -2.920692372
C -11.842747522 -5.957686354 -2.084175917
H -11.082114526 -4.996308413 -0.391886673
H -11.668727110 -6.348138411 -3.083438061
H -12.846596525 -6.062311877 -1.685781115

==============================
syn-6 (conformer11/12)
==============================
C 0.590415477 -3.769512508 -1.810954221
C 0.693820423 -3.279098709 -3.256430152
C -0.587987725 -2.543867260 -3.645827328
C -0.825524061 -1.363571448 -2.673115549
C -0.645698540 -1.594042698 -1.156310115
C 0.543338543 -2.542327652 -0.882528274
H 0.856938713 -4.124141358 -3.935896390
H 1.563666848 -2.617773988 -3.353042366
H -1.812936950 -0.930022896 -2.857922389
H -0.092171866 -0.595872717 -2.944649834
H 0.522525133 -2.866065148 0.160991812
H 1.472365642 -1.979400084 -1.030212942
C 1.784571028 -4.645425625 -1.434280529
C -0.476340964 -1.965572222 -5.059228780
C -0.690339667 -4.587608406 -1.614510518
C -1.714281326 -3.584652166 -3.656737168
N -1.629117189 -4.539483035 -2.563257934
H -2.477898505 -5.100139389 -2.400641338
H 2.711147893 -4.068052956 -1.505106477
H 1.672726695 -5.021663785 -0.416817774
H 1.864949883 -5.503832984 -2.106722474
H -0.249958954 -2.751012439 -5.785906782
H 0.318760623 -1.216473672 -5.114953125
H -1.412591403 -1.485940161 -5.362201297
C -0.381786448 -0.235314508 -0.474778599
H 0.542880304 0.203835882 -0.858666404
H -0.286621608 -0.353279075 0.606861995
H -1.201165112 0.460514026 -0.675712618
C -1.881716972 -2.156331767 -0.527642301
O -1.810817683 -2.452683242 0.802802838
N -3.055436469 -2.417471593 -1.063813117
C -5.623217525 -3.986333233 1.162883243
C -5.126524379 -3.482393237 -0.063361356
C -4.821471242 -3.945203999 2.307319495
H -5.718545121 -3.566011823 -0.963357337
C -3.793500812 -2.953643783 -0.061019100
C -3.483521274 -3.430007292 2.326538366
H -2.902294277 -3.454112918 3.235999391
C -3.028216163 -2.971267552 1.111731204
C -8.564284189 -5.725722181 2.765903598
C -8.906843993 -6.196421523 4.018555279
C -7.330276053 -5.101221690 2.552959010
H -9.865826460 -6.678979528 4.173850191
C -8.018800798 -6.050139194 5.092269454
C -6.462604265 -4.975500641 3.637291631
H -8.290448886 -6.419485543 6.075112791
C -6.791994290 -5.439253556 4.905841622
H -6.075046824 -5.322248577 5.709579345
H -9.228380941 -5.818575793 1.913771664
C -6.939540366 -4.614082647 1.223021403
O -5.230346927 -4.403674113 3.497518491
O -7.662813964 -4.750484885 0.233985330

H	-1.676637770	-4.137958156	-4.602461212
H	-2.688587413	-3.091564623	-3.595970242
O	-0.833926428	-5.269548484	-0.584768533
H	-5.885267928	-8.301488200	3.200000592
C	-4.930375181	-7.868695574	2.917808223
C	-3.935321455	-7.693377502	3.856360361
C	-4.741725897	-7.464025824	1.580408897
C	-2.714778234	-7.114280609	3.485731517
H	-4.101140498	-7.998845774	4.883843021
C	-2.496083242	-6.711245175	2.177483066
H	-1.934127223	-6.975451079	4.226039877
C	-3.504272753	-6.882326034	1.227728468
H	-1.561285018	-6.254141145	1.869146056
N	-3.317122159	-6.453394980	-0.069049592
C	-5.749292763	-7.577785323	0.582317282
C	-5.531990623	-7.112654569	-0.695983643
H	-6.704611241	-8.001367160	0.862822423
C	-4.258478446	-6.491192244	-1.061656963
O	-6.408366844	-7.156037093	-1.706959993
O	-4.030931497	-6.018155643	-2.181385258
H	-2.403524152	-6.035743694	-0.312176633
C	-7.708840261	-7.664656741	-1.438002632
C	-8.443316456	-7.761284074	-2.764405201
H	-8.216849200	-6.990812050	-0.737800036
H	-7.637190429	-8.662821279	-0.987293085
C	-9.823496322	-8.316787590	-2.569905890
H	-8.484351684	-6.762220356	-3.210754145
H	-7.864987308	-8.399571926	-3.439073070
C	-10.217275088	-9.515882899	-2.980648760
H	-10.523198301	-7.683952913	-2.025002914
H	-9.546587837	-10.177432680	-3.523035600
H	-11.221918846	-9.882448035	-2.797302003

==============================

syn-6 (conformer12/12)

==============================

C	0.587882083	-3.774010448	-1.817802043
C	0.689466634	-3.277285224	-3.261204118
C	-0.590755550	-2.535991328	-3.644044138
C	-0.821898417	-1.359217159	-2.665375368
C	-0.638505508	-1.596974436	-1.149997315
C	0.547807754	-2.551006738	-0.883740576
H	0.847942947	-4.119620923	-3.945083393
H	1.561347291	-2.618514853	-3.356850742
H	-1.808464430	-0.921716676	-2.845321092
H	-0.086894702	-0.592665173	-2.935632385
H	0.528453990	-2.879463466	0.158330911
H	1.478528162	-1.990831784	-1.031238363
C	1.779767246	-4.656231170	-1.448579251
C	-0.480674471	-1.951645986	-5.055082049
C	-0.695276133	-4.588397533	-1.621625010
C	-1.720351587	-3.573145353	-3.657054226
N	-1.636124500	-4.532992582	-2.567882268
H	-2.486092478	-5.092349189	-2.407441928
H	2.708349413	-4.082084360	-1.519340027
H	1.669406345	-5.036967404	-0.432636714
H	1.854873558	-5.511609250	-2.125457721
H	-0.258518846	-2.734516678	-5.785826424
H	0.316650323	-1.204806526	-5.109353604
H	-1.416121940	-1.467705790	-5.353637146
C	-0.367488522	-0.242370374	-0.463210710
H	0.557923356	0.194831275	-0.847528510
H	-0.269980763	-0.365479128	0.617651727
H	-1.184609184	0.457558514	-0.658966675
C	-1.874786339	-2.157710257	-0.520574039
O	-1.801683792	-2.459786785	0.808535534
N	-3.050222259	-2.414311744	-1.055211869
C	-5.616057496	-3.987520522	1.171057402
C	-5.121149736	-3.479574635	-0.054502875
C	-4.811817576	-3.952454341	2.313739159
H	-5.715387731	-3.559330092	-0.953395838
C	-3.787158266	-2.953086402	-0.053082426
C	-3.472933595	-3.440050304	2.331877564
H	-2.889359770	-3.469138945	3.239683340
C	-3.019403839	-2.977055138	1.117866774
C	-8.556410838	-5.729026707	2.773037712
C	-8.896985896	-6.204667183	4.024295664
C	-7.321863673	-5.105460406	2.560051995
H	-9.856242703	-6.686612718	4.179648621
C	-8.006386557	-6.064149571	5.096708094
C	-6.451643686	-4.985482457	3.642971635
H	-8.276454805	-6.437312604	6.078552406
C	-6.779151970	-5.454191935	4.910267506
H	-6.060374841	-5.341602544	5.713011584
H	-9.222457666	-5.817173217	1.921927072
C	-6.933484773	-4.613031241	1.231324445
O	-5.218899508	-4.415050048	3.503071168
O	-7.659099629	-4.743817999	0.243227600
H	-1.686294710	-4.122475979	-4.605238313
H	-2.693000187	-3.077344586	-3.592228648
O	-0.838388680	-5.274241682	-0.594375286
H	-5.880522081	-8.312480657	3.190995815
C	-4.925955360	-7.880115661	2.907043935
C	-3.927071472	-7.710363510	3.842509277
C	-4.741640490	-7.470155911	1.570660971
C	-2.706965928	-7.131781934	3.469686597
H	-4.089416939	-8.019863816	4.869329440
C	-2.492385415	-6.723740426	2.162370128
H	-1.923324223	-6.997417971	4.207650537
C	-3.504474915	-6.889249136	1.215725515
H	-1.558017837	-6.266964727	1.852327437
N	-3.321638601	-6.455557456	-0.079942982
C	-5.753342967	-7.577696968	0.575999491
C	-5.540324396	-7.107407798	-0.701061327
H	-6.708243499	-8.000938344	0.858423172
C	-4.267229722	-6.486335642	-1.068729847

O -6.420767575 -7.144766750 -1.708700256
O -4.042807047 -6.008113272 -2.186853421
H -2.408840341 -6.037778272 -0.325788290
C -7.721229311 -7.651700179 -1.436616508
C -8.461504270 -7.741085656 -2.760278889
H -8.224843262 -6.979677524 -0.731485590
H -7.649934152 -8.651889473 -0.990358320
C -9.842477369 -8.293271333 -2.562020574
H -8.501520806 -6.740169155 -3.202560863
H -7.887944657 -8.378426706 -3.439894396
C -10.241734279 -9.489409419 -2.976063754
H -10.537855412 -7.660583579 -2.011424889
H -9.575475647 -10.150727809 -3.524158297
H -11.246695061 -9.853657847 -2.789862991

==============================
anti-6 (conformer2/5)
==============================
C 0.404949530 -3.901266975 -2.331122266
C 0.198860452 -2.993619634 -3.546287403
C -0.958336875 -2.027508465 -3.289592378
C -0.657516958 -1.186759311 -2.026676324
C -0.147032517 -1.908775221 -0.761614524
C 0.848373464 -3.029260370 -1.139378794
H -0.013845728 -3.596220040 -4.437647472
H 1.124343995 -2.440194208 -3.747244033
H -1.530109343 -0.575117859 -1.775083151
H 0.134780976 -0.484232094 -2.309448599
H 1.030126352 -3.656786856 -0.263846000
H 1.801448286 -2.562775285 -1.414743003
C 1.476106109 -4.956243553 -2.605579374
C -1.134331590 -1.060136429 -4.462704991
C -0.903893424 -4.626260990 -1.989488892
C -2.232302908 -2.878565097 -3.194940539
N -2.053520642 -4.112328055 -2.450020622
H -2.908428118 -4.655882203 -2.267875206
H 2.434566234 -4.472993838 -2.816732173
H 1.588271503 -5.615976174 -1.744723283
H 1.207458682 -5.572785736 -3.467961643
H -1.288966051 -1.602853440 -5.399699889
H -0.251158613 -0.426462366 -4.583884133
H -1.998181643 -0.406370008 -4.304854668
C 0.562679349 -0.891195994 0.154583253
H 1.433545527 -0.464293600 -0.351151224
H 0.895457348 -1.376258744 1.074709984
H -0.113263793 -0.074004730 0.422474748
C -1.248727348 -2.520982765 0.042127487
O -2.549844645 -2.299530245 -0.277247618
N -1.097495840 -3.373280601 1.058438756
C -4.185632884 -5.070571046 2.324901182
C -2.801516784 -4.735634178 2.360907196
C -5.030055868 -4.428164437 1.431579560
H -2.136455456 -5.254390311 3.034144609
C -2.336849574 -3.761756799 1.383534168
C -4.606593907 -3.387148685 0.511881579
H -5.306872966 -2.972894147 -0.197942240
C -3.257942603 -3.123127685 0.540652348
C -6.754227281 -7.479122169 3.675810518
C -8.075070707 -7.799850931 3.424906013
C -6.132508991 -6.436474315 2.982420358
H -8.548863387 -8.613233010 3.964048067
C -8.806620793 -7.076339184 2.474354114
C -6.881736397 -5.735386858 2.040850589
H -9.843449310 -7.329196965 2.278996527
C -8.213503001 -6.036893341 1.778878161
H -8.745968657 -5.461950321 1.029942761
H -6.153789621 -8.020593937 4.397965064
C -4.710953466 -6.115410816 3.197398533
O -6.330637453 -4.720977691 1.308062689
O -4.024561363 -6.701768679 4.029343456
H -2.560478142 -3.131686934 -4.210485469
H -3.038816750 -2.305595624 -2.727934108
O -0.888724528 -5.659035131 -1.304067357
H -5.145174738 -10.003075875 2.165664730
C -4.257124379 -9.452908928 1.869610586
C -3.062013509 -9.640823872 2.529537801
C -4.354961401 -8.529040274 0.818265726
C -1.927867631 -8.914898743 2.143389532
H -3.000386551 -10.342731110 3.353555333
C -1.992776968 -8.005310773 1.108311863
H -0.990297027 -9.060628575 2.668955012
C -3.211520460 -7.807021541 0.450197601
H -1.128328031 -7.426967040 0.798622864
N -3.312343340 -6.868195551 -0.557552435
C -5.578301196 -8.270872296 0.109427335
C -5.642105716 -7.323150718 -0.854878817
H -6.455111840 -8.845232242 0.383998985
C -4.454769172 -6.524812906 -1.211693227
O -6.731369533 -7.001764005 -1.584680502
O -4.480802199 -5.606938407 -2.040557699
H -2.448079938 -6.378413880 -0.844941879
C -7.860456739 -7.851776275 -1.471876756
C -8.870071905 -7.419398584 -2.521881441
H -8.289511151 -7.781653522 -0.462713566
H -7.562806225 -8.895667536 -1.638513485
C -10.113194383 -8.256422343 -2.453845329
H -9.106389729 -6.362109892 -2.355804650
H -8.406515865 -7.495552606 -3.509543397
C -10.515482598 -9.091207641 -3.403576738
H -10.699473076 -8.170931399 -1.538920495
H -9.957294106 -9.208316598 -4.328696966
H -11.417125202 -9.685359444 -3.297783197

==============================
anti-6 (conformer3/5)
==============================

C 0.336729584 -3.914552279 -2.131451419
C 0.291172446 -3.153832956 -3.459118399
C -0.861197839 -2.151067812 -3.446496588
C -0.682996659 -1.171866281 -2.259652073
C -0.307084911 -1.739477579 -0.872152592
C 0.688209153 -2.916854857 -1.013086562
H 0.161096534 -3.852080696 -4.294355232
H 1.248074649 -2.641337024 -3.615921773
H -1.568996579 -0.534985111 -2.169547526
H 0.141611662 -0.506345038 -2.538543215
H 0.762131729 -3.437198111 -0.055405045
H 1.678124360 -2.505338179 -1.242902104
C 1.388128671 -5.023717755 -2.166853710
C -0.878631326 -1.323535756 -4.734516694
C -1.025163625 -4.559845526 -1.843641842
C -2.158383398 -2.971060091 -3.407256688
N -2.094858195 -4.115176693 -2.516104417
H -2.979653205 -4.617462318 -2.361908545
H 2.381115872 -4.595321728 -2.331342551
H 1.385556636 -5.577408662 -1.227652976
H 1.182138750 -5.729659455 -2.976019005
H -0.935732447 -1.970608229 -5.614625402
H 0.026989008 -0.716905293 -4.822701100
H -1.739589899 -0.647494987 -4.757020469
C 0.347770938 -0.626805404 -0.028922233
H 1.276294926 -0.289799580 -0.498530242
H 0.575604067 -0.994055587 0.973905883
H -0.322176951 0.232711983 0.063056019
C -1.492078720 -2.226224847 -0.101155884
O -2.747359380 -2.103323621 -0.604921760
N -1.458033299 -2.894234529 1.056894574
C -4.668735261 -4.436232186 2.219596465
C -3.300293202 -4.065258174 2.361149002
C -5.401722268 -3.977741571 1.135612851
H -2.722003106 -4.437000362 3.193127519
C -2.723258532 -3.260369421 1.293750237
C -4.874111407 -3.098386434 0.107592912
H -5.483083607 -2.832421650 -0.743569809
C -3.541015036 -2.799352903 0.250151041
C -7.368556678 -6.656386262 3.640464843
C -8.646861370 -7.049065972 3.291841625
C -6.673237374 -5.727986409 2.859572097
H -9.178173713 -7.773170819 3.900121397
C -9.261360681 -6.514105343 2.152224399
C -7.306808067 -5.213296342 1.732041361
H -10.265256731 -6.823517431 1.880773176
C -8.594489203 -5.590590323 1.366500620
H -9.034361114 -5.163786488 0.472401662
H -6.857182596 -7.054114561 4.509643889
C -5.289363192 -5.338931167 3.183753925
O -6.675561460 -4.322642723 0.907792166
O -4.703512962 -5.765710993 4.174283361
H -2.375825630 -3.332153266 -4.419708358
H -2.997916512 -2.339069423 -3.101429912
O -1.120555969 -5.472434824 -1.008909017
H -5.823409029 -9.340366957 2.426252278
C -4.904240228 -8.814903110 2.185757502
C -3.812729941 -8.889122364 3.023896913
C -4.858195452 -8.035089984 1.020161088
C -2.639098504 -8.192531410 2.707613843
H -3.864616223 -9.476555470 3.933620392
C -2.562293496 -7.426095968 1.563386137
H -1.785094355 -8.245403115 3.373936998
C -3.677189784 -7.342876917 0.721731006
H -1.665659306 -6.872907829 1.303273688
N -3.639638387 -6.543394616 -0.403538971
C -5.971448785 -7.897703023 0.121803588
C -5.900919782 -7.089388251 -0.961486243
H -6.878525336 -8.443987156 0.351113045
C -4.677152780 -6.321298036 -1.255302489
O -6.875186839 -6.897538294 -1.875755714
O -4.586956389 -5.528056060 -2.200009279
H -2.739613857 -6.091909470 -0.640197918
C -7.993180726 -7.767312012 -1.821328720
C -8.818034871 -7.538108121 -3.076729027
H -8.586213825 -7.569515496 -0.917022615
H -7.654741408 -8.811016400 -1.777035477
C -10.032723045 -8.418900929 -3.095490154
H -9.105153220 -6.481158272 -3.113072016
H -8.187762164 -7.732407751 -3.949190421
C -10.233794640 -9.407008858 -3.958135318
H -10.776781251 -8.228237524 -2.322421327
H -9.513137346 -9.630500031 -4.740282625
H -11.123507112 -10.026734657 -3.920634870

==============================
anti-6 (conformer4/5)
==============================

C 0.120478084 -3.989897895 -2.170478423
C 0.118746759 -3.250180642 -3.510302496
C -0.944318023 -2.153373414 -3.488937489
C -0.687985890 -1.186161647 -2.302038108
C -0.250553242 -1.747869025 -0.926101319
C 0.624978752 -3.016373190 -1.092728398
H -0.090082916 -3.945058216 -4.332060487
H 1.113140957 -2.825845048 -3.693329738
H -1.555676291 -0.531166978 -2.170261221
H 0.132889783 -0.535396526 -2.621622872
H 0.699825618 -3.524364382 -0.128616815
H 1.636953668 -2.707050385 -1.379984768
C 1.033387547 -5.216406088 -2.211737397
C -0.900717821 -1.325422297 -4.776737116
C -1.296497415 -4.477685476 -1.834599070
C -2.302836781 -2.866486554 -3.446587125
N -2.310626751 -4.035555444 -2.587642254
H -3.230239572 -4.457273436 -2.408655431

H	2.065896094	-4.910212075	-2.404126558
H	0.988085047	-5.751874362	-1.262640812
H	0.728694704	-5.904817575	-3.005203357
H	-1.022633612	-1.964384741	-5.656135069
H	0.053942681	-0.800772176	-4.873509311
H	-1.698663135	-0.575605784	-4.791605351
C	0.561324906	-0.671968366	-0.180573838
H	1.473001077	-0.436085103	-0.736133297
H	0.835421902	-1.022179343	0.816105295
H	-0.022501063	0.246563855	-0.072504832
C	-1.393746853	-2.120058112	-0.032222748
O	-2.658889040	-2.188444914	-0.510756084
N	-1.281885105	-2.567850695	1.212320037
C	-4.305183650	-4.210079667	2.709110744
C	-2.988393687	-3.650496885	2.770511679
C	-5.092260692	-4.010781006	1.590978089
H	-2.374829025	-3.787352388	3.647605714
C	-2.508257302	-2.981559526	1.552695783
C	-4.679618451	-3.238683080	0.418402378
H	-5.300920416	-3.218852945	-0.465688241
C	-3.378572985	-2.781145433	0.480150988
C	-6.727762008	-6.432911025	4.553503655
C	-7.971454456	-6.988928813	4.325427967
C	-6.138489688	-5.598883572	3.599811401
H	-8.422790706	-7.637615670	5.068339402
C	-8.650931469	-6.719692988	3.131630679
C	-6.836958217	-5.344033293	2.425054020
H	-9.626159781	-7.159800861	2.951318739
C	-8.089001463	-5.893170435	2.174733613
H	-8.584147206	-5.671169466	1.236692062
H	-6.164278415	-6.625009757	5.459394177
C	-4.795229407	-5.031136032	3.807206030
O	-6.310970200	-4.543372241	1.442859429
O	-4.137825327	-5.254646697	4.819858764
H	-2.570076194	-3.180845817	-4.462056033
H	-3.081900413	-2.176561286	-3.104252350
O	-1.480603893	-5.285519276	-0.910407368
H	-6.425694849	-9.331610135	2.039799947
C	-5.512085745	-8.757457014	1.919953475
C	-4.518064532	-8.815823950	2.874247672
C	-5.370109743	-7.938775577	0.789839595
C	-3.349395766	-8.059944214	2.716477631
H	-4.645434089	-9.437543846	3.753390459
C	-3.181607105	-7.250678383	1.611374275
H	-2.576983528	-8.093638232	3.476378673
C	-4.197958444	-7.181441549	0.653508582
H	-2.288514835	-6.649632957	1.475460048
N	-4.071637611	-6.337808462	-0.434268953
C	-6.367994776	-7.839636966	-0.240577480
C	-6.204238858	-7.005885071	-1.293675608
H	-7.254504872	-8.456048829	-0.150284230
C	-5.013738938	-6.142100911	-1.396621171
O	-7.038071149	-6.869962655	-2.345060608
O	-4.867865060	-5.301855371	-2.291264977
H	-3.168635675	-5.854533099	-0.572640150
C	-8.072446041	-7.828855508	-2.485063679
C	-8.650299752	-7.687765493	-3.883846661
H	-8.845852997	-7.669274091	-1.719642982
H	-7.668984249	-8.840417431	-2.346262888
C	-9.770806367	-8.661029279	-4.106540150
H	-8.999178168	-6.657326232	-4.012477654
H	-7.847671536	-7.851978295	-4.608427322
C	-9.713998191	-9.698129530	-4.932413895
H	-10.674178338	8.497821340	-3.519314092
H	-8.829566503	-9.896760076	-5.531992908
H	-10.546210929	-10.384778017	-5.046589224

==============================

anti-6 (conformer5/5)

==============================

C	0.101783323	-4.001648072	-2.095173006
C	0.048549667	-3.234486685	-3.418046139
C	-0.993771062	-2.123174151	-3.328193999
C	-0.626755836	-1.158142979	-2.170653850
C	-0.126589498	-1.725977634	-0.817838806
C	0.644119908	-3.062916273	-1.005194663
H	-0.203041498	-3.912573017	-4.242355754
H	1.038926376	-2.816781614	-3.635595066
H	-1.460876278	-0.472016780	-1.989354922
H	0.191359362	-0.540141577	-2.555849631
H	0.686419666	-3.589834817	-0.048380082
H	1.678004673	-2.825986218	-1.282593094
C	1.018472739	-5.222344684	-2.203804151
C	-1.020869678	-1.298483984	-4.619466477
C	-1.293540260	-4.507639805	-1.724645643
C	-2.365293980	-2.805784460	-3.195401720
N	-2.342435766	-4.013016634	-2.388144991
H	-3.248366088	-4.459646364	-2.206262240
H	2.035714975	-4.904597940	-2.450182059
H	1.031591032	-5.771836609	-1.262054700
H	0.674028966	-5.903242217	-2.987537438
H	-1.220646662	-1.933803895	-5.487297513
H	-0.062720036	-0.797630869	-4.783844797
H	-1.798318532	-0.528285030	-4.579096722
C	0.840992548	-0.706918208	-0.181789016
H	1.720805034	-0.569198846	-0.816941329
H	1.169043379	-1.052760723	0.800836906
H	0.350155916	0.261866404	-0.055811880
C	-1.230292009	-1.888328496	0.188317865
O	-2.523121133	-2.183609014	-0.267033463
N	-0.979501405	-2.544189514	1.418544850
C	-3.610990720	-4.779056860	2.728247398
C	-2.365838701	-4.140208018	2.733342592
C	-4.518153397	-4.537962122	1.689111980
H	-1.659393285	-4.351698699	3.524502312
C	-2.060667826	-3.249096551	1.665771256

C -4.250629161 -3.653092565 0.597011223
H -4.958571960 -3.561340612 -0.215444422
C -3.043636449 -3.043684129 0.633206595
C -5.745949586 -7.278912140 4.597706308
C -6.995862474 -7.850051436 4.463081612
C -5.284827851 -6.351650950 3.659503208
H -7.348698429 -8.569704715 5.193572282
C -7.807367159 -7.501145699 3.377820233
C -6.109637241 -6.022686381 2.588335873
H -8.789073997 -7.950553240 3.269417683
C -7.371843430 -6.586516721 2.436607486
H -7.973221837 -6.305764736 1.580169899
H -5.083636975 -7.527036686 5.419231876
C -3.949213756 -5.744122587 3.784791675
O -5.719200478 -5.137642153 1.619985729
O -3.181887148 -6.024157188 4.694345801
H -2.725093489 -3.074526443 -4.194987220
H -3.091002625 -2.110462066 -2.761510119
O -1.429833245 -5.383704482 -0.853066496
H -5.905174531 -10.088645483 1.729195896
C -5.023348606 -9.465464021 1.615955306
C -3.956409808 -9.599083512 2.480415615
C -4.997951739 -8.508447259 0.591157127
C -2.829669757 -8.780370196 2.334710166
H -3.993122465 -10.332235701 3.278559479
C -2.776053445 -7.833312568 1.331834801
H -1.997945172 -8.876227512 3.023641127
C -3.865701795 -7.690861693 0.466103365
H -1.917803526 -7.181308402 1.207145934
N -3.855785437 -6.709515496 -0.505726972
C -6.087026182 -8.307367707 -0.326727882
C -6.046230709 -7.321219463 -1.252904390
H -6.944702801 -8.964678248 -0.247425947
C -4.887755893 -6.410996491 -1.338817638
O -6.990027616 -7.045638330 -2.174575624
O -4.846258001 -5.445500588 -2.109135436
H -2.996607420 -6.140846703 -0.614629649
C -8.085367770 -7.938267500 -2.276146945
C -8.903166475 -7.533312698 -3.491196385
H -8.694140210 -7.895488332 -1.361020186
H -7.723920324 -8.969126975 -2.388306511
C -10.124823095 -8.393357519 -3.632567651
H -9.182759480 -6.479766263 -3.379995743
H -8.272339178 -7.609945155 -4.381280461
C -10.333306260 -9.249004390 -4.625014693
H -10.868847337 -8.304569022 -2.841256641
H -9.613297597 -9.366682722 -5.430247599
H -11.229526726 -9.858158823 -4.676642567

DFT Optimized Geometries

==============================

syn-4

================================

C 0.322120479 -4.015341332 -2.373215594
C 0.197101643 -3.123677184 -3.619899726
C -0.838349424 -2.013056254 -3.381187209
C -0.385924963 -1.148721501 -2.174663997
C -0.015992070 -1.892308232 -0.868154307
C 0.835906399 -3.146333226 -1.192136777
H -0.097133849 -3.729310222 -4.487095554
H 1.177282343 -2.686887616 -3.853535041
H -1.145009178 -0.391709578 -1.955658422
H 0.517983375 -0.614147987 -2.492523510
H 0.947257745 -3.766462887 -0.299146212
H 1.838390352 -2.796558172 -1.468624422
C 1.310131858 -5.164095294 -2.614187963
C -0.942937617 -1.093772853 -4.608232526
C -1.052638458 -4.612234654 -2.020555770
C -2.208127451 -2.700580435 -3.187830425
N -2.146854330 -3.939339585 -2.415266046
H -3.055661740 -4.363381307 -2.179351277
H 2.306021849 -4.764839639 -2.833376143
H 1.368808906 -5.810480231 -1.736606385
H 0.994288852 -5.780019265 -3.462114632
H -1.200955702 -1.664835530 -5.506363182
H 0.006874235 -0.582544735 -4.796439704
H -1.713890247 -0.329214258 -4.461832545
C 0.790050600 -0.948499631 0.058089775
H 1.726176168 -0.648813692 -0.423710769
H 1.032710444 -1.446094576 1.002029470
H 0.211039023 -0.047722187 0.282915700
C -1.262777915 -2.270082835 -0.114743314
O -1.149457179 -3.334021571 0.767652878
N -2.444749838 -1.758488190 -0.205147878
C -5.141979481 -3.647285694 1.575998727
C -4.613965260 -2.613552818 0.786201885
C -4.276536460 -4.598046078 2.169577424
H -5.295051068 -1.919725056 0.308088516
C -3.241525773 -2.565125741 0.609213485
C -2.889459173 -4.568341447 2.002532812
H -2.263152730 -5.346847079 2.417519636
C -2.422654974 -3.542713369 1.207254861
C -8.397129455 -5.181667812 2.776753155
C -8.800261649 -6.292364335 3.501077140
C -7.033949248 -4.931756622 2.546646997
H -9.855138649 -6.481705027 3.672683086
C -7.834820190 -7.178875092 4.010105675
C -6.087939646 -5.817646534 3.081566647
H -8.147602537 -8.056283106 4.568378327
C -6.481507092 -6.947694002 3.807308611
H -5.717747591 -7.623937805 4.173982639
H -9.110164173 -4.480746521 2.355084597
C -6.605413652 -3.787122068 1.719506456
O -4.746777145 -5.647350041 2.912940261

O	-7.409128072	-3.024950839	1.184385574
H	-2.623706390	-2.939780901	-4.176075965
H	-2.910006128	-2.023886163	-2.690520767
O	-1.135926982	-5.671318465	-1.368759034
C	-4.718176387	-9.414356719	1.649456828
H	-5.646264191	-9.851756738	2.007703110
C	-3.495565444	-9.904398149	2.083057179
C	-4.783346654	-8.337486221	0.744148772
C	-2.301956093	-9.328667208	1.614763257
H	-3.459676997	-10.734056251	2.782319395
C	-3.572357242	-7.771451834	0.284537568
C	-6.019070298	-7.763465391	0.289007662
C	-2.333056360	-8.269169941	0.719368093
H	-1.345716189	-9.714687720	1.954663964
H	-1.422733817	-7.811012700	0.344244629
N	-3.629302310	-6.709368606	-0.594982293
C	-4.768966720	-6.082663787	-1.027004120
H	-2.734749896	-6.305657458	-0.924912606
C	-6.029747247	-6.672755921	-0.529605279
H	-6.945212374	-8.200134576	0.644135450
O	-7.118392390	-6.001849298	-0.958938935
O	-4.725999756	-5.096868237	-1.777476757
C	-8.403694857	-6.470036229	-0.545718979
C	-9.425541684	-5.405975312	-0.935424370
H	-8.421124868	-6.623232369	0.540281700
H	-8.622171197	-7.432340163	-1.030224451
C	-10.797980466	-5.787516502	-0.453120603
H	-9.101572099	-4.460847249	-0.480940675
H	-9.415320583	-5.273151941	-2.022216379
C	-11.824294108	-6.108956295	-1.240185551
H	-10.927262215	-5.819612393	0.629856795
H	-11.733311595	-6.095033705	-2.323884124
H	-12.792051503	-6.389915800	-0.835225496

==============================

anti-4

==============================

C	4.914935430	1.263576600	-0.228129156
C	5.810844754	0.355820705	0.632797474
C	5.319252721	-1.097909837	0.564876157
C	5.350174472	-1.575864064	-0.911516639
C	4.682297288	-0.667603915	-1.973438064
C	5.039984660	0.818465086	-1.709000948
H	5.810567746	0.701055855	1.674858587
H	6.846814184	0.425165932	0.275027791
H	4.943884464	-2.590034001	-0.983009441
H	6.407625187	-1.647996426	-1.195110898
H	4.418227712	1.457015127	-2.341131205
H	6.085146991	0.970850051	-2.007993425
C	5.342216817	2.732287998	-0.111082325
C	6.236596568	-2.026625649	1.376900439
C	3.445442730	1.139953868	0.224248936
C	3.918555244	-1.130069421	1.212539690
N	3.070140709	-0.000860492	0.839970501
H	2.082364191	-0.058316250	1.125350619
H	6.371526203	2.852000629	-0.465087840
H	4.682471299	3.369258228	-0.703044803
H	5.293596602	3.071640581	0.928085217
H	6.303790596	-1.702070268	2.420473501
H	7.249711928	-2.033338583	0.961811252
H	5.862601261	-3.056785014	1.366384848
C	5.167682121	-1.078391110	-3.384145714
H	6.253490238	-0.963465482	-3.461138252
H	4.693512775	-0.454602548	-4.146590760
H	4.917767307	-2.124061416	-3.591810411
C	3.183834317	-0.797737608	-1.970295381
O	2.627229636	-1.923329141	-1.377734234
N	2.332094520	0.055436012	-2.431044598
C	-1.251405012	-0.662443607	-1.619111116
C	-0.193616002	0.056776663	-2.198317618
C	-1.000777285	-1.878387934	-0.939811049
H	-0.408148055	1.002072585	-2.682382309
C	1.087131235	-0.456225566	-2.072694675
C	0.277347761	-2.431158433	-0.833978264
H	0.441352086	-3.350868266	-0.288154033
C	1.284530336	-1.679058694	-1.401300308
C	-4.960788659	-0.465733755	-0.842085962
C	-5.900665186	-1.195934259	-0.131609493
C	-3.630375352	-0.907097517	-0.933900023
H	-6.925559199	-0.846955383	-0.056432820
C	-5.519134307	-2.393090246	0.498474963
C	-3.267676028	-2.098842802	-0.292589592
H	-6.248970808	-2.961004728	1.066737430
C	-4.210862380	-2.850746339	0.418256961
H	-3.894732601	-3.769847796	0.899012725
H	-5.210337083	0.464087415	-1.342578286
C	-2.624389004	-0.119830077	-1.676005064
O	-1.997540350	-2.593570774	-0.330176019
O	-2.910104740	0.914329420	-2.274920104
H	4.038855609	-1.127860013	2.304334170
H	3.394706229	-2.053808989	0.945841840
O	2.630653693	2.046878834	-0.012589418
H	-4.184578887	3.761127031	0.079657846
C	-3.125612244	3.768246617	-0.163471220
C	-2.599691936	4.749005989	-0.988066564
C	-2.303014445	2.754803087	0.363310465
C	-1.230623779	4.739582581	-1.306868298
H	-3.245501268	5.520678230	-1.394959316
C	-0.393514297	3.756780924	-0.801330389
H	-0.822946731	5.505895490	-1.959221700
C	-0.929529346	2.763030943	0.033464577
H	0.665598153	3.730130207	-1.039137591
N	-0.122889668	1.761453473	0.534772560
C	-2.792546047	1.695896851	1.199439559
C	-1.967481838	0.703177257	1.639313354
H	-3.849030088	1.684808652	1.441046386

C -0.536058914 0.692945952 1.283038905
O -2.324343997 -0.355491490 2.405074415
O 0.248635389 -0.208775564 1.622747828
H 0.884823851 1.795748013 0.308934096
C -3.660375744 -0.384719013 2.907764292
C -3.784817830 -1.611375224 3.805819165
H -4.377374975 -0.442207395 2.080398064
H -3.862989067 0.536499902 3.474784394
C -5.133224043 -1.670234018 4.468613720
H -3.607818774 -2.508883686 3.205934824
H -2.990034389 -1.560048232 4.561467634
C -6.032244994 -2.637392826 4.287820917
H -5.373745194 -0.839691775 5.133964937
H -5.829955804 -3.482556307 3.634290583
H -6.993322137 -2.627913192 4.793157990

==============================

syn-7

==============================

C 0.3460434802 -3.9740838414 -2.6220447213
C 0.3022306324 -2.8997876508 -3.7220220293
C -0.8379303013 -1.9079606767 -3.4453252092
C -0.6381385483 -1.2559220684 -2.0498991487
C -0.2780886006 -2.1685691303 -0.8493861408
C 0.7055044314 -3.2921416366 -1.2806480138
H 0.1547530958 -3.3686714514 -4.7036716262
H 1.2675048429 -2.3774820758 -3.7589886425
H -1.5180366064 -0.6561852284 -1.7941977522
H 0.1992366827 -0.5549283999 -2.1555432037
H 0.7765071047 -4.0439525409 -0.4899638655
H 1.7016259727 -2.8461439038 -1.3949167322
C 1.3878062126 -5.0556003522 -2.9427984664
C -0.8516701371 -0.7832237361 -4.4946594938
C -1.0278969919 -4.6472580397 -2.4768572536
C -2.1592517260 -2.6902778547 -3.5731463300
N -2.0852448254 -4.0469640640 -3.0414903057
H -2.9957969790 -4.5033886722 -2.9046030608
H 2.3885635578 -4.6128776260 -2.9947671451
H 1.3783999335 -5.8322743497 -2.1754828971
H 1.1740794788 -5.5296222422 -3.9065926427
H -0.9382002427 -1.1922042304 -5.5072140027
H 0.0694326744 -0.1923657111 -4.4488575909
H -1.6956544284 -0.1028803969 -4.3321903167
C 0.3995272453 -1.3028029597 0.2463649287
H 1.3166727841 -0.8520541387 -0.1461973894
H 0.6565805946 -1.9082674370 1.1192894569
H -0.2682525967 -0.4978724004 0.5697347677
C -1.5034968069 -2.7655779071 -0.2065123329
O -1.2340645399 -3.6201265476 0.8549959490
N -2.7611680757 -2.5638938283 -0.4298831681
C -5.1236427987 -4.3977682712 1.8543603493
C -4.7808223310 -3.5588391689 0.7755509912
C -4.1066113883 -4.9984449067 2.6399103627
H -5.5709204191 -3.1142853149 0.1830001184
C -3.4303039447 -3.3397022142 0.5262053362
C -2.7484759665 -4.8107917414 2.3858581379
H -1.9934043004 -5.3015965130 2.9853695442
C -2.4634122867 -3.9802204500 1.3212672107
C -8.0583558706 -6.0224129620 3.6185917802
C -8.2648465623 -6.8951472219 4.6763034874
C -6.7585871000 -5.6287317895 3.2514768203
H -9.2726509810 -7.1905143370 4.9521014433
C -7.1663615924 -7.3925083762 5.3981473071
C -5.6773953691 -6.1411287817 3.9868857931
H -7.3245400285 -8.0735325703 6.2292982982
C -5.8725428209 -7.0163058600 5.0584336695
H -5.0051613489 -7.3849599041 5.5952782092
H -8.8831854116 -5.6098214354 3.0462108784
C -6.5326793465 -4.7102312179 2.1256051891
O -4.3834825404 -5.8156360497 3.7010656868
O -7.4643951474 -4.2375201529 1.4535822528
H -2.4391960668 -2.7538218481 -4.6329670599
H -2.9644690924 -2.1682865440 -3.0454566687
O -1.1380571766 -5.7184870133 -1.8446739722
C -5.0718969975 -8.4757535759 1.7979158117
H -6.0289336867 -8.7024791902 2.2568027456
C -3.9036579456 -9.0005901872 2.3007748789
C -5.0554300934 -7.5854912456 0.6712465797
C -2.6607679557 -8.6749816179 1.7132999334
H -3.9358334064 -9.6603913906 3.1625844535
C -3.7655607976 -7.2705688344 0.0830374965
C -6.2005275206 -6.9905620154 0.1678912946
C -2.5957927233 -7.8137612213 0.6151197752
H -1.7449621495 -9.0901731005 2.1222727087
H -1.6459884895 -7.5447406580 0.1632161571
N -3.7135884985 -6.4306270544 -1.0048353205
C -4.8208281762 -5.8021126869 -1.5753431501
H -2.7838250132 -6.1926387180 -1.3938995589
C -6.1015987530 -6.0970132405 -0.9754100557
H -7.1606363903 -7.1930787059 0.6220481724
O -7.1510346779 -5.5575378461 -1.5927860937
O -4.6783478006 -5.0517773349 -2.5598344349
C -8.4825202488 -5.7552700156 -1.0739301887
C -9.4274563260 -4.9947032244 -1.9992487610
H -8.5220782610 -5.3702176792 -0.0492025164
H -8.7189735248 -6.8264145495 -1.0786328347
C -10.8524247163 -5.1172887739 -1.5319956162
H -9.1134723685 -3.9432901041 -2.0067700768
H -9.3181532031 -5.3778416020 -3.0195889235
C -11.8214261983 -5.7439573409 -2.2001904542
H -11.0756261200 -4.6670745464 -0.5639672565
H -11.6387586254 -6.2084201104 -3.1667327665
H -12.8333684838 -5.8100364140 -1.8110411340

==============================

syn-7-TS

==============================

C -1.6728204328 -0.7353969629 -2.0323378787
C -2.2792985263 0.4027791912 -2.8709194360
C -3.6022379484 0.8763457348 -2.2487629328
C -3.3412396873 1.3740942878 -0.8026971870
C -2.5514301627 0.4325730380 0.1397050585
C -1.3496713599 -0.1967808790 -0.6135288741
H -2.4518835476 0.0603378684 -3.8998513599
H -1.5642720800 1.2345616974 -2.9272427533
H -4.2888732917 1.6483092747 -0.3281190083
H -2.7500550601 2.2950975810 -0.8876077607
H -0.9058378089 -0.9932680841 -0.0100705281
H -0.5872814520 0.5841090024 -0.7292478083
C -0.3872774222 -1.2732781708 -2.6756256714
C -4.1969333715 2.0436754242 -3.0535311962
C -2.6812012724 -1.8865086495 -1.8923234265
C -4.5930036958 -0.3034384808 -2.3217503979
N -3.9855615423 -1.6022709163 -2.0440762914
H -4.6474922292 -2.3691756531 -1.8597983289
H 0.3656168526 -0.4798133294 -2.7368754123
H 0.0139262703 -2.1035061229 -2.0912296656
H -0.5817434346 -1.6391539073 -3.6891593969
H -4.3398875110 1.7646807000 -4.1032951247
H -3.5354648947 2.9161028527 -3.0250726375
H -5.1695354710 2.3463815521 -2.6487476623
C -2.0209152219 1.2471554476 1.3487070442
H -1.3414974438 2.0346173391 1.0063634537
H -1.4774336269 0.6008206793 2.0444912025
H -2.8496981738 1.7153300016 1.8890487183
C -3.4520368624 -0.6259258265 0.7243159527
O -2.8108749308 -1.7143962478 1.2999054435
N -4.7398097222 -0.6371539258 0.8282413971
C -6.2031617966 -3.6712972934 2.4758238958
C -6.2418523669 -2.4271413876 1.8278984364
C -4.9596481786 -4.2836520310 2.7702283425
H -7.2037946969 -1.9862998232 1.5961437084
C -5.0377462861 -1.8339308971 1.4885364582
C -3.7337034193 -3.7061337643 2.4268703990
H -2.8007882802 -4.2193201242 2.6194926529
C -3.8263961563 -2.4907520874 1.7822314741
C -8.3957634203 -6.5221190161 3.6671017239
C -8.2260847230 -7.7934493752 4.1935676443
C -7.2870253132 -5.7063103540 3.3925752518
H -9.0877888348 -8.4213922004 4.3974024323
C -6.9316089752 -8.2684162550 4.4642870859
C -6.0043308340 -6.2045091234 3.6619389086
H -6.7947969090 -9.2632405574 4.8784572764
C -5.8186446216 -7.4799645006 4.2045780455
H -4.8078634740 -7.8245842671 4.3918815533
H -9.3771289304 -6.1171310068 3.4427542029
C -7.4663770304 -4.3591477233 2.8135316133
O -4.8702440791 -5.4913207315 3.4052209349
O -8.5727659931 -3.8608245521 2.6204545252
H -5.0299562495 -0.3387910641 -3.3292425160
H -5.4105369518 -0.1561379701 -1.6090510812
O -2.2886852783 -3.0342074022 -1.6061294761
C -3.7467927662 -7.9708256194 1.3415276354
H -4.3743868639 -8.7027750278 1.8415074894
C -2.3681434851 -8.0206423438 1.4598753727
C -4.3820289299 -6.9518768359 0.5773479317
C -1.5701314957 -7.0581221450 0.8206111480
H -1.9029263461 -8.8041073695 2.0509956282
C -3.5438886013 -5.9851674238 -0.0695044739
C -5.7703034422 -6.8321339062 0.4480284993
C -2.1593550219 -6.0424919921 0.0576486233
H -0.4893932565 -7.0953036581 0.9176425251
H -1.5561170827 -5.2822124595 -0.4298124456
N -4.1341834464 -4.9660409082 -0.8149746926
C -5.4791218249 -4.6800328572 -0.8422141149
H -3.5008399642 -4.2436441247 -1.1968667212
C -6.3606694225 -5.7252515442 -0.3108985217
H -6.4493613728 -7.5148005487 0.9429014392
O -7.6467024129 -5.4339273221 0.0204785845
O -5.8916825391 -3.6195987013 -1.3540135842
C -8.4784097514 -5.1201857176 -1.1063207653
C -8.3648635014 -6.3129228085 -2.0748884506
H -8.1423346141 -4.1914698855 -1.5733080264
H -9.4882652746 -4.9941816559 -0.7103257680
C -6.9182177028 -6.6497339275 -2.3219764809
H -8.8811830558 -7.1779232348 -1.6441714193
H -8.8692183345 -6.0551809661 -3.0155495954
C -6.4067650224 -7.9253356873 -2.1632902241
H -6.3574312268 -5.9395560996 -2.9264953913
H -6.9859682681 -8.7072551950 -1.6820684163
H -5.3903147053 -8.1675203818 -2.4532835542

==============================

syn-7-TS'

==============================

C 0.2068106213 -2.4881850966 -1.3294117735
C 0.1048602063 -1.2600473814 -2.2495817075
C -1.2175414627 -0.5198533775 -1.9973184479
C -1.2955469728 -0.0887279582 -0.5082929586
C -0.9302550409 -1.1270577380 0.5841748210
C 0.2725696128 -2.0046797771 0.1391850226
H 0.1601870367 -1.5709309300 -3.3010035319
H 0.9598800658 -0.5952023488 -2.0672013696
H -2.2871882614 0.3242944592 -0.2951513251
H -0.5811824654 0.7355286197 -0.3895988825
H 0.3685235024 -2.8648875301 0.8077601892
H 1.1856066539 -1.4057444724 0.2484777241
C 1.4581590105 -3.3187389200 -1.6490201480
C -1.3062879968 0.7500185086 -2.8600226287
C -1.0324440743 -3.3842956674 -1.4924075190
C -2.3563652802 -1.4644629387 -2.4255325874
N -2.1151643176 -2.8614639558 -2.0850238314

H -2.9505555977 -3.4632244796 -2.1465010072
H 2.3616207286 -2.7211707197 -1.4843277334
H 1.4956441859 -4.2085633236 -1.0174597990
H 1.4517228785 -3.6465366063 -2.6939122784
H -1.2014038445 0.5092352834 -3.9233559738
H -0.5148997036 1.4601600634 -2.5968589536
H -2.2699062958 1.2537285730 -2.7218122753
C -0.5396305964 -0.3629590797 1.8786072784
H 0.3256420750 0.2790848070 1.6854790001
H -0.2831682454 -1.0577804034 2.6822629791
H -1.3666884963 0.2684678345 2.2191400068
C -2.1039713371 -1.9936995461 0.9619271121
O -1.8262512034 -2.9345534964 1.9499753964
N -3.3412259805 -1.9505951089 0.5933982777
C -5.6397126090 -4.3438073930 2.3580471942
C -5.3026989423 -3.3586354527 1.4099140853
C -4.6453117135 -4.8784083013 3.2166913138
H -6.0727318320 -2.9727236279 0.7528936706
C -3.9849032225 -2.9289331311 1.3619871877
C -3.3069988826 -4.4879650755 3.1519972757
H -2.5610368210 -4.9357572106 3.7950183782
C -3.0283366071 -3.5185986104 2.2114529541
C -8.5137400431 -6.4491033595 3.6508805335
C -8.7236334332 -7.4106122108 4.6268489587
C -7.2412677040 -5.8811828090 3.4631900134
H -9.7101750454 -7.8409724605 4.7681967987
C -7.6534751680 -7.8257830935 5.4380172163
C -6.1886250342 -6.3117929888 4.2854102869
H -7.8143232743 -8.5788211108 6.2038379463
C -6.3871773726 -7.2825942527 5.2717807061
H -5.5437640364 -7.5880822467 5.8810149522
H -9.3145143303 -6.0991644201 3.0070510224
C -7.0138260998 -4.8622220432 2.4222165040
O -4.9243864625 -5.8187240914 4.1695416019
O -7.9094847820 -4.4918341555 1.6542352083
H -2.4904060903 -1.3947883021 -3.5134303677
H -3.2987973334 -1.1648038155 -1.9551392361
O -1.0132824775 -4.5570135528 -1.0659132869
C -4.7893674487 -8.1538385835 1.8100448682
H -5.7377180946 -8.5455087146 2.1667764155
C -3.6013430732 -8.5312808477 2.4211597142
C -4.8009726654 -7.2357701094 0.7358200875
C -2.3879338729 -7.9950884758 1.9716239864
H -3.6172322379 -9.2298093654 3.2522304574
C -3.5568715138 -6.7014900393 0.2940407591
C -6.0153271333 -6.8139580527 0.1061066810
C -2.3646423120 -7.0797589107 0.9173915995
H -1.4559589915 -8.2802083986 2.4511571506
H -1.4349344838 -6.6298218344 0.5814423361
N -3.5334678358 -5.7708170721 -0.7363981958
C -4.6561608465 -5.1718833406 -1.2876495757
H -2.6200824494 -5.3547322313 -0.9747664922
C -5.9306019993 -5.6867629981 -0.8418149498
H -6.9278406883 -6.9192045118 0.6789012234
O -6.9900596637 -5.1231652016 -1.4362467325
O -4.5405342769 -4.2551585725 -2.1263204027
C -8.3198544558 -5.6332487455 -1.2988288000
C -8.5354650166 -6.9117395557 -2.1602410803
H -8.9451155726 -4.8213449316 -1.6766116905
H -8.5660456309 -5.7884451473 -0.2448916742
C -8.0064189998 -8.1460404412 -1.5002097553
H -9.6125016978 -6.9998979593 -2.3431777243
H -8.0506687159 -6.7287365538 -3.1265481655
C -6.6654613096 -8.4188888721 -1.4087895902
H -8.6932239806 -8.6920445195 -0.8550537035
H -6.3102152813 -9.2811265611 -0.8526331946
H -5.9627832893 -7.9964781474 -2.1231694528

==============================

syn-8

==============================

C 0.270571639 -4.107661395 -2.638191523
C 0.201585656 -3.017314438 -3.721620614
C -0.838742151 -1.952752536 -3.340287632
C -0.443606609 -1.314833333 -1.983035307
C -0.135990094 -2.278736265 -0.811669518
C 0.730333342 -3.467430317 -1.303023406
H -0.059316050 -3.466347183 -4.689059937
H 1.191682202 -2.557143741 -3.839823301
H -1.213031577 -0.601533496 -1.670310759
H 0.472792060 -0.737100995 -2.159721979
H 0.786766865 -4.235592830 -0.526946347
H 1.749079854 -3.090516140 -1.458835443
C 1.254640217 -5.217762443 -3.033536048
C -0.888214218 -0.834687019 -4.394379187
C -1.118762706 -4.726865648 -2.429399219
C -2.219592091 -2.640472589 -3.314612369
N -2.192706035 -3.997699034 -2.774411221
H -3.113894567 -4.428524088 -2.617738756
H 2.262631652 -4.804404777 -3.147790009
H 1.273151962 -6.002138164 -2.274526636
H 0.963376320 -5.677103619 -3.983750902
H -1.095482200 -1.242318296 -5.389727548
H 0.066005733 -0.299553171 -4.442492303
H -1.671051917 -0.104817358 -4.158179660
C 0.642334417 -1.510383987 0.289694823
H 1.593623350 -1.141071577 -0.106924484
H 0.853558405 -2.162064602 1.142928476
H 0.059565490 -0.655001808 0.645210692
C -1.406654562 -2.748047728 -0.149671167
O -1.281250970 -3.851555928 0.682773233
N -2.581212208 -2.209597632 -0.165804566
C -5.165680791 -3.901834027 1.962493790
C -4.685033473 -2.937852953 1.064370679
C -4.298188152 -4.898459998 2.469269816
H -5.374200278 -2.201783829 0.668499120

C	-3.350297250	-2.996615431	0.699903971
C	-2.948326302	-4.978705426	2.112494694
H	-2.319521125	-5.777790702	2.482160734
C	-2.526170460	-4.012391826	1.224157341
C	-8.348854802	-5.251699977	3.513670227
C	-8.728557392	-6.323907631	4.306588096
C	-7.002986974	-5.057713880	3.165408996
H	-9.772084178	-6.469197359	4.568373091
C	-7.755221269	-7.223454674	4.773300849
C	-6.049619075	-5.971025460	3.636849440
H	-8.047975501	-8.063772352	5.396298179
C	-6.416819034	-7.052175465	4.445063571
H	-5.645495488	-7.734105606	4.785176304
H	-9.068160461	-4.532634885	3.134935003
C	-6.600526127	-3.923850547	2.309403697
O	-4.723563920	-5.869738775	3.334697261
O	-7.402099576	-3.080140989	1.910573280
H	-2.612642744	-2.691870010	-4.338943648
H	-2.923676429	-2.052353911	-2.716971573
O	-1.229757641	-5.863502966	-1.928581265
C	-4.658514260	-8.837772464	1.854045616
H	-5.550189175	-9.114836182	2.407803285
C	-3.423467456	-9.350490521	2.203693173
C	-4.796757009	-7.918510354	0.774952552
C	-2.271801777	-8.969626663	1.489823383
H	-3.340356824	-10.046842574	3.033001842
C	-3.609592770	-7.553243016	0.067423762
C	-6.016028177	-7.341316683	0.409507689
C	-2.366101989	-8.072689106	0.424381934
H	-1.301982063	-9.370862945	1.768284175
H	-1.486033507	-7.752372877	-0.125675299
N	-3.701290344	-6.631950817	-0.970340344
C	-4.826715192	-5.985552714	-1.367542920
H	-2.808729021	-6.338567384	-1.411830822
C	-6.161726779	-6.374082608	-0.716057602
H	-6.916706637	-7.564460471	0.972887847
O	-6.742924159	-5.170330538	-0.184556189
O	-4.794074435	-5.105068353	-2.237803880
C	-7.877485027	-4.766232419	-0.954609963
C	-8.458096643	-6.076851225	-1.473251644
H	-7.559089464	-4.118404902	-1.782479001
H	-8.528468636	-4.200245084	-0.284036388
C	-7.186039026	-6.858212586	-1.854600846
H	-9.000201412	-6.596875464	-0.674401898
H	-9.133786561	-5.952932210	-2.324042169
C	-7.342906645	-8.326692547	-1.975699248
H	-6.792573499	-6.443222034	-2.789289591
H	-6.670506933	-8.911059344	-2.593957548
H	-8.025664294	-8.864163437	-1.325334775

==============================

syn-8'

==============================

C	-4.890515082	1.401093971	-0.141482532
C	-5.875485691	0.653435155	-1.056492979
C	-5.412182451	-0.797125261	-1.262976990
C	-5.311325047	-1.510852040	0.111637422
C	-4.561528365	-0.781934256	1.254512402
C	-4.910989246	0.731055340	1.255990184
H	-5.948068137	1.160936084	-2.027397557
H	-6.878107456	0.677275029	-0.608535450
H	-4.883581875	-2.510132271	-0.021349582
H	-6.340486252	-1.652817093	0.465587311
H	-4.240196247	1.267047075	1.932572345
H	-5.927463398	0.837891394	1.655890055
C	-5.274789003	2.880550259	-0.001997240
C	-6.419486317	-1.574978884	-2.126641982
C	-3.459799822	1.307989027	-0.702822726
C	-4.076152160	-0.739707498	-2.027702590
N	-3.193319523	0.334549421	-1.586583159
H	-2.215419077	0.239013085	-1.901539473
H	-6.266539869	2.972753224	0.454613329
H	-4.544889590	3.407032102	0.616127695
H	-5.303314489	3.370461106	-0.980930389
H	-7.391071451	-1.648313758	-1.626187325
H	-6.065407516	-2.593375499	-2.324383248
H	-6.575382194	-1.078822947	-3.090809020
C	-4.986067067	-1.400039336	2.613238191
H	-6.061568488	-1.266200431	2.768478700
H	-4.454974305	-0.922603691	3.441689295
H	-4.765233841	-2.471976595	2.633805701
C	-3.070980555	-0.982793364	1.162775993
O	-2.321697426	-0.191866441	2.027367142
N	-2.380898901	-1.815070391	0.456453077
C	1.338320624	-1.643863836	0.943943200
C	0.145225130	-2.114375781	0.365150359
C	1.298913191	-0.650771501	1.954890205
H	0.199542102	-2.849323866	-0.428666014
C	-1.048709441	-1.578513163	0.816980245
C	0.105987177	-0.082644551	2.405904514
H	0.107902081	0.709060834	3.142904622
C	-1.032733157	-0.570933399	1.803775789
C	5.115101607	-1.930508258	0.762055029
C	6.218353441	-1.350412879	1.367635633
C	3.812375071	-1.557320937	1.135317398
H	7.221631710	-1.642848645	1.073936204
C	6.033401290	-0.380920935	2.369144702
C	3.652413380	-0.589480383	2.137168561
H	6.895516259	0.076951664	2.845512184
C	4.757755622	0.003356992	2.756924675
H	4.586526590	0.757378553	3.516991491
H	5.214069942	-2.687499175	-0.009380962
C	2.636529990	-2.157398516	0.472524225
O	2.428385831	-0.166255891	2.555098758
O	2.745953511	-3.001341386	-0.418433615
H	-4.282985064	-0.597813730	-3.097254392

H -3.531785977 -1.683276048 -1.917390134
O -2.578190021 2.097570897 -0.307448708
C 3.282601375 2.421511405 0.601093386
H 4.340484312 2.183691329 0.517883555
C 2.842649639 3.288694670 1.598926637
C 2.387955934 1.822830964 -0.290387896
C 1.477226518 3.572233243 1.706744737
H 3.554303245 3.735970110 2.286314270
C 1.017981169 2.107590846 -0.162345853
C 2.891581233 0.900596381 -1.383154432
C 0.565809311 2.987304244 0.835094535
H 1.119673197 4.246247932 2.480423033
H -0.500511047 3.176408606 0.918921000
N 0.093717986 1.494903475 -0.998986547
C 0.376537485 0.425412975 -1.824034624
H -0.903205097 1.708689550 -0.843157258
C 1.774082748 0.084971453 -1.969253295
H 3.629559369 0.223477759 -0.933075679
O 1.981816888 -0.990629315 -2.748751016
O -0.540785057 -0.197768271 -2.395938364
C 3.303616568 -1.389982755 -3.130681414
C 3.981340896 -0.355872153 -4.058476623
H 3.143793485 -2.330566849 -3.662062917
H 3.906462222 -1.605970541 -2.242723891
C 4.531378577 0.838377441 -3.346562912
H 4.777686239 -0.870422901 -4.607970009
H 3.221349357 -0.062493268 -4.799524508
C 3.659655358 1.709807158 -2.503894528
H 5.609049028 0.945797654 -3.263323513
H 2.895988741 2.227883701 -3.104350616
H 4.256145086 2.484551879 -2.012701956

==============================
os(syn-8)
==============================

C 0.275979539 -4.105217409 -2.638765999
C 0.212164328 -3.007244849 -3.714524246
C -0.835007837 -1.949020572 -3.334300811
C -0.451214065 -1.317887389 -1.970754531
C -0.149343209 -2.287402335 -0.802436374
C 0.721048154 -3.472938422 -1.294091570
H -0.039014299 -3.450407597 -4.687233385
H 1.201669714 -2.542896302 -3.821070745
H -1.224221074 -0.608018415 -1.659366316
H 0.465260608 -0.737421514 -2.137958422
H 0.769845214 -4.246328640 -0.522496541
H 1.741419355 -3.095167077 -1.436737114
C 1.269628182 -5.207247495 -3.032666222
C -0.880054613 -0.825061676 -4.382560869
C -1.112540366 -4.733533839 -2.450108027
C -2.213683089 -2.641592161 -3.322535000
N -2.186451827 -4.002622030 -2.791667707
H -3.107863755 -4.439182188 -2.651146796
H 2.276449660 -4.787694883 -3.133613889
H 1.284543086 -5.997008710 -2.279205774
H 0.990209432 -5.661323777 -3.988927547
H -1.078606980 -1.227446575 -5.382008041
H 0.072974466 -0.286739944 -4.420281300
H -1.666765780 -0.098917192 -4.147795106
C 0.622635758 -1.523301033 0.306602351
H 1.575448682 -1.151042374 -0.083700439
H 0.830462450 -2.178650639 1.157772962
H 0.037091165 -0.670298469 0.663132972
C -1.421161851 -2.761485507 -0.146812688
O -1.292993728 -3.860074936 0.691505424
N -2.597404896 -2.227123811 -0.166423822
C -5.178003240 -3.918743982 1.967955639
C -4.700615821 -2.958575023 1.063310333
C -4.306408172 -4.906994779 2.483727704
H -5.392904225 -2.229285076 0.660328252
C -3.365159774 -3.013883642 0.700804116
C -2.956042079 -4.983797859 2.128777233
H -2.323997671 -5.777147586 2.505004556
C -2.537564789 -4.022625360 1.233177798
C -8.358914592 -5.280532203 3.516147738
C -8.733600325 -6.347403406 4.318890678
C -7.013039692 -5.080372531 3.170219638
H -9.776816111 -6.497146267 4.579473800
C -7.755488807 -7.235688518 4.797139253
C -6.054944761 -5.982526051 3.653618242
H -8.044375463 -8.071625350 5.427822242
C -6.417465616 -7.059025370 4.470179921
H -5.642632465 -7.732091591 4.819964661
H -9.081953086 -4.569722849 3.128993633
C -6.614479140 -3.950198268 2.306990907
O -4.728722007 -5.874313292 3.355403512
O -7.421260238 -3.117346532 1.895412752
H -2.599266906 -2.687586735 -4.349950212
H -2.923983768 -2.059764006 -2.726163951
O -1.223381965 -5.879012208 -1.968940038
C -4.644280653 -8.788111180 1.855330699
H -5.534208798 -9.056589497 2.416387906
C -3.407693478 -9.293673872 2.211095119
C -4.786063549 -7.887086802 0.761584078
C -2.258266629 -8.922733528 1.488476277
H -3.322057711 -9.975938448 3.051855287
C -3.601185786 -7.531131806 0.045931763
C -6.007814397 -7.319545348 0.388170315
C -2.356382172 -8.043111387 0.408460329
H -1.287157073 -9.317827473 1.771358810
H -1.477897162 -7.729793049 -0.147961134
N -3.696320266 -6.628871918 -1.008393842
C -4.821255744 -5.984714554 -1.410746474
H -2.805683790 -6.352205784 -1.464547429
C -6.154877351 -6.350159594 -0.741311889
H -6.906825530 -7.538988250 0.955101352

O	-6.706861317	-5.134248537	-0.207571441
O	-4.789391134	-5.121361270	-2.298284739
C	-7.856317196	-4.727746097	-0.955188354
C	-8.457829811	-6.037356481	-1.454265554
H	-7.551455316	-4.085660117	-1.793469644
H	-8.490368954	-4.154164773	-0.273931917
C	-7.196538059	-6.832902228	-1.850738009
H	-8.990142402	-6.547005836	-0.641726803
H	-9.148163650	-5.914024810	-2.293773308
C	-7.363031368	-8.307834086	-1.929492336
H	-6.824810597	-6.440593997	-2.803467270
H	-6.775315088	-8.905787215	-2.623075164
H	-7.968024792	-8.832080552	-1.192447195

===============================

os(syn-8')

===============================

C	-4.913149766	1.395249433	-0.190619844
C	-5.915045158	0.597794348	-1.042315005
C	-5.405136257	-0.836830279	-1.247047763
C	-5.196851854	-1.518553614	0.133070441
C	-4.473992865	-0.729358112	1.256560844
C	-4.866814870	0.774566845	1.226140578
H	-6.053496671	1.082791956	-2.017281259
H	-6.894585983	0.593362375	-0.545301551
H	-4.699742128	-2.484652478	-0.005007648
H	-6.199974189	-1.736513744	0.520212792
H	-4.182268676	1.346219688	1.859223593
H	-5.869091910	0.871035553	1.662660983
C	-5.324091044	2.870949504	-0.084645684
C	-6.427697704	-1.674962928	-2.033337782
C	-3.502693648	1.319716916	-0.804383097
C	-4.120941693	-0.741586543	-2.092508705
N	-3.269411494	0.387872153	-1.738528033
H	-2.304292378	0.318120571	-2.100516531
H	-6.298362619	2.958388822	0.409247155
H	-4.580819066	3.431891398	0.485538065
H	-5.403194894	3.326163137	-1.077454040
H	-7.367359601	-1.770134056	-1.478530783
H	-6.046276201	-2.684792785	-2.224231909
H	-6.653922549	-1.211917050	-2.999902050
C	-4.889308886	-1.329296248	2.626791034
H	-5.971753320	-1.237641376	2.760416123
H	-4.393410549	-0.809751265	3.450453275
H	-4.625633334	-2.390655105	2.680271727
C	-2.976782034	-0.872208669	1.177967384
O	-2.283459342	-0.215026125	2.192852311
N	-2.232248305	-1.546226540	0.367392828
C	1.454851356	-1.411355051	1.067454374
C	0.303262307	-1.779083644	0.346748825
C	1.343945425	-0.617277891	2.236825625
H	0.409512797	-2.363141150	-0.559708977
C	-0.922836553	-1.343745006	0.820923488
C	0.119100433	-0.144057308	2.710127064
H	0.064011169	0.490159986	3.584909932
C	-0.977264125	-0.523685193	1.966838922
C	5.237176641	-1.653240077	1.061857819
C	6.294764256	-1.208287345	1.838966856
C	3.910802764	-1.356023984	1.417642235
H	7.316974689	-1.441148720	1.558012299
C	6.039056895	-0.452757637	2.996472255
C	3.680222737	-0.598216984	2.575130518
H	6.865392077	-0.101839877	3.607773720
C	4.738241919	-0.145955652	3.369735827
H	4.514348526	0.442030060	4.253059484
H	5.392252921	-2.238849344	0.161571556
C	2.785008680	-1.806775024	0.578962804
O	2.429193968	-0.248145794	2.981219387
O	2.955693450	-2.437099605	-0.468691720
H	-4.396493316	-0.647008933	-3.151454835
H	-3.524145979	-1.653601225	-1.985759259
O	-2.613465988	2.105281467	-0.416597743
C	3.278497034	2.011913287	0.517315842
H	4.319309692	1.714048038	0.413092916
C	2.885846166	2.797948722	1.599449261
C	2.354461146	1.574174739	-0.434609993
C	1.541575318	3.158976049	1.731874895
H	3.617553155	3.113890256	2.336572824
C	1.003709500	1.930033291	-0.279779030
C	2.802155191	0.746702330	-1.623144714
C	0.601563295	2.729827866	0.801579486
H	1.221378613	3.768404382	2.572566256
H	-0.451353243	2.976110080	0.905512307
N	0.048519491	1.463448753	-1.175764783
C	0.267045787	0.444270822	-2.081527202
H	-0.932875243	1.721232120	-0.994618974
C	1.641684227	0.031981456	-2.251466248
H	3.521617837	0.002203426	-1.262875589
O	1.799450458	-0.979957691	-3.120516867
O	-0.683672986	-0.074330993	-2.701673421
C	3.118052604	-1.394345158	-3.509087978
C	3.813946011	-0.347950022	-4.393047490
H	2.942905207	-2.307349885	-4.082488309
H	3.705068286	-1.656945200	-2.623588035
C	4.343295684	0.860348981	-3.682615111
H	4.635380868	-0.850018426	-4.919125967
H	3.084894904	-0.061697043	-5.172804252
C	3.564266153	1.636509913	-2.671395910
H	5.258745253	1.307867441	-4.062503096
H	2.812416125	2.291683056	-3.145934804
H	4.238224302	2.304257005	-2.122526888

===============================

syn-9

===============================

C	-1.5204839	-0.6413449	-1.9461852

C -2.0711799 0.51778245 -2.7964084
C -3.4750533 0.9109183 -2.311099
C -3.4160695 1.3158903 -0.81274396
C -2.6489248 0.39207 0.16746746
C -1.3481014 -0.14522256 -0.48934332
H -2.1140747 0.2248285 -3.853629
H -1.3874785 1.3748267 -2.7300007
H -4.430755 1.4809511 -0.4348056
H -2.9054623 2.2865245 -0.7737987
H -0.92434835 -0.9427062 0.12688439
H -0.61846113 0.6744784 -0.5047854
C -0.16659203 -1.1275202 -2.4825232
C -4.012282 2.1140475 -3.104875
C -2.5075476 -1.8238641 -1.9521716
C -4.402033 -0.2902051 -2.581376
N -3.7688527 -1.5816271 -2.3387847
H -4.411801 -2.3854253 -2.3284347
H 0.57227415 -0.3199059 -2.4320807
H 0.19135922 -1.9778396 -1.8986439
H -0.25183254 -1.4467422 -3.5266306
H -4.0285273 1.9002519 -4.179258
H -3.3859425 2.9988241 -2.9480534
H -5.0332036 2.366508 -2.7955825
C -2.2718425 1.2110099 1.4320395
H -1.6229113 2.0487838 1.1568885
H -1.7430964 0.58765936 2.1582408
H -3.169464 1.6142236 1.9117875
C -3.5009584 -0.748798 0.6627234
O -2.8178267 -1.6754717 1.44563
N -4.7697444 -0.964735 0.55707103
C -6.054385 -3.979722 2.4018188
C -6.159362 -2.8517673 1.5659937
C -4.795731 -4.35766 2.9359481
H -7.1236744 -2.6065264 1.138937
C -5.00565 -2.1286578 1.3040898
C -3.6223857 -3.6481252 2.672268
H -2.670788 -3.9713264 3.0727828
C -3.7779973 -2.5503933 1.8544759
C -8.011549 -6.907977 3.803065
C -7.7584405 -8.051316 4.544893
C -6.9817533 -5.9964266 3.5155292
H -8.557952 -8.75394 4.758458
C -6.460353 -8.296169 5.0264072
C -5.6933556 -6.2666492 3.9970868
H -6.2581735 -9.189809 5.610015
C -5.4268827 -7.409337 4.758855
H -4.414301 -7.5794635 5.108035
H -9.00116 -6.6759787 3.421757
C -7.2536 -4.7787023 2.721702
O -4.6357617 -5.4436884 3.7520614
O -8.389133 -4.4807763 2.352744
H -4.7313356 -0.26164976 -3.6289191
H -5.295523 -0.22974987 -1.9505553
O -2.1346307 -2.9609756 -1.598464
C -4.3888974 -7.7150173 1.3489443
H -5.1320405 -8.391332 1.7635785
C -3.0649827 -7.784387 1.782145
C -4.79413 -6.769097 0.40373337
C -2.1241345 -6.8908844 1.2646753
H -2.773352 -8.52109 2.5248108
C -3.8371456 -5.875702 -0.10344447
C -6.2130384 -6.6910496 -0.07350293
C -2.5048916 -5.937453 0.32397512
H -1.091034 -6.9299617 1.5987399
H -1.7918421 -5.2198477 -0.07250729
N -4.189311 -4.88422 -1.0369493
C -5.443638 -4.5878863 -1.4624013
H -3.4386797 -4.2409253 -1.3383151
C -6.577797 -5.478348 -0.9883867
H -6.889205 -6.73256 0.7837023
O -7.615955 -4.644446 -0.52741456
O -5.6732655 -3.6481767 -2.2308679
C -8.841172 -5.3628545 -0.6897173
C -8.676813 -6.124418 -2.0176754
H -9.652821 -4.6323204 -0.6843593
H -9.0007925 -6.0466557 0.1567616
C -7.1770983 -6.4599147 -2.0549495
H -9.316602 -7.010576 -2.079659
H -8.92065 -5.4614463 -2.853128
C -6.675196 -7.6635203 -1.2072991
H -6.7618484 -6.39388 -3.062355
H -7.4369783 -8.392526 -0.9138608
H -5.8427825 -8.197878 -1.6723047

==============================
syn-7m-TS
==============================
C -1.524121771 -1.012013249 -1.757275979
C -2.008648902 -0.011812113 -2.821367307
C -3.327491805 0.641863101 -2.378766583
C -3.113802131 1.373932878 -1.026188787
C -2.448333632 0.566517762 0.116278750
C -1.243003039 -0.240079066 -0.439628944
H -2.150921090 -0.524212771 -3.782074658
H -1.236475668 0.753044679 -2.979617298
H -4.065050140 1.784189719 -0.672109429
H -2.454004036 2.228042611 -1.227153587
H -0.871218240 -0.932749840 0.319783293
H -0.437575444 0.475700237 -0.647771818
C -0.246719345 -1.730319362 -2.212645031
C -3.797729279 1.678184616 -3.412050838
C -2.612698737 -2.058506817 -1.468945488
C -4.387268144 -0.475984475 -2.308222100
N -3.887985528 -1.725240460 -1.738187989
H -4.614011755 -2.392166610 -1.441911766
H 0.561263713 -1.006601889 -2.366082401

H 0.065931807 -2.463895830 -1.466995805
H -0.413865294 -2.260732415 -3.156008337
H -3.904699137 1.224356131 -4.403366530
H -3.079798534 2.501082620 -3.495186131
H -4.766822610 2.105140709 -3.129323303
C -1.945071655 1.539130348 1.212947498
H -1.193917064 2.220386267 0.799776107
H -1.490154206 0.987238677 2.041309086
H -2.773179904 2.134664939 1.609159366
C -3.451556303 -0.340490475 0.784564633
O -2.921078858 -1.426128961 1.466346163
N -4.740182498 -0.247676798 0.830162977
C -6.490792975 -3.084996879 2.551555233
C -6.412782618 -1.872657389 1.844564470
C -5.308140120 -3.772857530 2.924260695
H -7.329369628 -1.377875495 1.546986614
C -5.155661761 -1.378935873 1.541272955
C -4.031244032 -3.299006855 2.612629459
H -3.141893324 -3.854850210 2.880177538
C -4.009434097 -2.104696547 1.924690174
C -8.938382893 -5.574459726 4.037511962
C -8.887398343 -6.782296226 4.714818122
C -7.757803775 -4.922639601 3.643975366
H -9.803433790 -7.282319685 5.013046762
C -7.643104071 -7.363721765 5.009798016
C -6.528133813 -5.519679413 3.955743293
H -7.599419173 -8.314097123 5.533732291
C -6.462053534 -6.739250057 4.634923583
H -5.489691103 -7.171617844 4.844314066
H -9.879854742 -5.099512452 3.780920406
C -7.807872930 -3.671119958 2.862499282
O -5.333250282 -4.966334060 3.594720736
O -8.868611236 -3.177337746 2.474423427
H -4.755962258 -0.682440871 -3.322294283
H -5.240729552 -0.151030074 -1.704731281
O -2.315290972 -3.146981122 -0.941960330
C -4.081414404 -8.549108527 -2.408781028
H -4.737137997 -9.330644837 -2.781373793
C -2.710604193 -8.741791525 -2.368049034
C -4.667878962 -7.329586376 -1.967340769
C -1.869466487 -7.724437486 -1.891786101
H -2.285778276 -9.681349381 -2.709442541
C -3.785454911 -6.310509933 -1.478104007
C -6.046535034 -7.077750728 -1.986186406
C -2.408918300 -6.509807516 -1.450875675
H -0.794059155 -7.873003548 -1.866611841
H -1.768882078 -5.705612254 -1.100096223
N -4.324778067 -5.100390029 -1.036599844
C -5.639107904 -4.718437301 -1.189416597
H -3.652697236 -4.344555681 -0.834536516
C -6.578828475 -5.793576964 -1.516466609
H -6.755991919 -7.808959886 -2.354727974
O -7.838474364 -5.491344121 -1.929419873
O -5.975249248 -3.530471045 -1.004584798
C -8.651472624 -4.906509191 -0.894256191
C -8.637449513 -5.889120792 0.287641948
H -9.648705792 -4.783640550 -1.322912072
H -8.251909860 -3.932672218 -0.601582249
C -7.220731259 -6.251044187 0.634600753
H -9.199891604 -6.791256878 0.022779730
H -9.137120687 -5.408606915 1.136725467
C -6.784676432 -7.554010892 0.775991553
H -6.613792740 -5.460670709 1.065167900
H -7.409460658 -8.391966646 0.483607374
H -5.786910170 -7.778666128 1.135741937

===============================

syn-7m-TS'

===============================

C 0.149469404 -2.482143889 -1.271077429
C -0.059834076 -1.359695092 -2.302696922
C -1.487149542 -0.805689804 -2.191882834
C -1.733424975 -0.282914869 -0.749670495
C -1.259741779 -1.140520474 0.454387776
C 0.075480596 -1.869112759 0.143176136
H 0.111398962 -1.741572863 -3.317261683
H 0.676682959 -0.563298482 -2.131425948
H -2.793409120 -0.038750285 -0.621446704
H -1.185137271 0.663802423 -0.673048366
H 0.257957842 -2.642367634 0.894486364
H 0.889562350 -1.138431993 0.229323770
C 1.518982397 -3.154136625 -1.460501580
C -1.695924529 0.373344962 -3.158309815
C -0.934142854 -3.565669301 -1.445837099
C -2.447642554 -1.936176404 -2.615951590
N -2.004879191 -3.257095056 -2.198882313
H -2.560862455 -4.051633137 -2.538197597
H 2.321684334 -2.423025735 -1.312683902
H 1.642995742 -3.973373315 -0.749583503
H 1.614619072 -3.566958751 -2.470100334
H -1.477643305 0.077013260 -4.189934538
H -1.039008329 1.211986061 -2.903915845
H -2.730379850 0.734631782 -3.122580421
C -1.046065635 -0.195935115 1.669965902
H -0.281745863 0.549727704 1.429805912
H -0.720167931 -0.754863124 2.550569747
H -1.973343005 0.330696768 1.918486210
C -2.297460107 -2.138674722 0.897189971
O -1.905702943 -2.905741433 1.992105764
N -3.513282003 -2.336625426 0.508531166
C -5.450964487 -4.863285691 2.510458169
C -5.255539092 -3.925110435 1.481160781
C -4.409631751 -5.139968350 3.428296677
H -6.075490485 -3.709422007 0.806790773
C -4.017737780 -3.311426849 1.382084392
C -3.154216647 -4.533211092 3.345469038

H	-2.368564219	-4.769410845	4.051516460
C	-3.004935683	-3.643547740	2.304217741
C	-7.990790964	-7.258677592	3.989267039
C	-8.063857446	-8.151475418	5.046930472
C	-6.822826318	-6.513642789	3.759636693
H	-8.969196282	-8.725369744	5.217994143
C	-6.959109357	-8.313175287	5.899600645
C	-5.732285968	-6.694293638	4.621342626
H	-7.012588431	-9.012000372	6.729421588
C	-5.792727013	-7.588767157	5.693925747
H	-4.925432386	-7.698913647	6.335903377
H	-8.820390342	-7.107715485	3.306122074
C	-6.735645264	-5.578081908	2.622513081
O	-4.553267701	-6.025335570	4.459922889
O	-7.665024474	-5.413068093	1.831183276
H	-2.539942127	-1.938981811	-3.710064148
H	-3.445894619	-1.753483464	-2.198696924
O	-0.790802501	-4.694643634	-0.941150402
C	-5.404081497	-8.451842310	0.784589369
H	-6.366596036	-8.950014511	0.706002628
C	-4.660222827	-8.546040337	1.956864713
C	-4.935835904	-7.713125754	-0.322325234
C	-3.426025652	-7.897247538	2.050740885
H	-5.043843776	-9.114663763	2.798325350
C	-3.682598202	-7.043138628	-0.198339576
C	-5.676182941	-7.567182804	-1.538860833
C	-2.939862681	-7.144495071	0.976018697
H	-2.845774184	-7.959262822	2.966149144
H	-1.988670610	-6.624375498	1.043132991
N	-3.203580990	-6.274634000	-1.262267720
C	-3.782248378	-6.204017721	-2.519275703
H	-2.306427630	-5.780937897	-1.148154725
C	-4.978990651	-6.978707495	-2.703555362
H	-6.493293395	-8.246040601	-1.756789273
O	-5.633926148	-7.033331490	-3.876733969
O	-3.288421464	-5.457737240	-3.400066308
C	-5.939273624	-5.809402709	-4.578120822
C	-6.476216967	-4.731405674	-3.594328551
H	-6.706285054	-6.106997877	-5.298528130
H	-5.054973778	-5.437727558	-5.100169253
C	-7.354576586	-5.321189122	-2.536120622
H	-5.617384279	-4.229668304	-3.138023062
H	-7.013303173	-3.989701813	-4.201124185
C	-6.884752167	-5.665897237	-1.283917209
H	-8.336986852	-5.675563050	-2.846258835
H	-7.546306346	-6.050695826	-0.516109593
H	-5.981026395	-5.191270936	-0.913477248

==============================

syn-8m

==============================

C	-1.500670244	-0.981801512	-1.787968793
C	-2.029241040	0.016836842	-2.833422331
C	-3.354702887	0.636228479	-2.362215723
C	-3.126462427	1.371287861	-1.014655250
C	-2.441947758	0.562307329	0.114878927
C	-1.231639370	-0.223900261	-0.458268809
H	-2.174860702	-0.490599723	-3.796314947
H	-1.278869342	0.801817405	-2.997540396
H	-4.074899529	1.774652968	-0.645776861
H	-2.474869567	2.229528812	-1.224808168
H	-0.847417104	-0.922609896	0.289718163
H	-0.436346245	0.505198993	-0.658420743
C	-0.206400229	-1.650156502	-2.269103679
C	-3.873547172	1.661321013	-3.383123894
C	-2.553116935	-2.062778840	-1.502003475
C	-4.385687168	-0.507205527	-2.267826254
N	-3.844780273	-1.744485695	-1.705550631
H	-4.542889409	-2.418167982	-1.363317478
H	0.573727232	-0.896725039	-2.422673167
H	0.141512794	-2.382941056	-1.538576099
H	-0.368165505	-2.172523040	-3.217869913
H	-3.992772635	1.206342469	-4.372572651
H	-3.178069510	2.501680447	-3.481291442
H	-4.845729675	2.064275190	-3.076810955
C	-1.938656895	1.530466746	1.215545256
H	-1.200167014	2.225130813	0.801798538
H	-1.468540215	0.975583669	2.033472027
H	-2.770111885	2.110976613	1.626221422
C	-3.439625584	-0.350686260	0.783805549
O	-2.920633514	-1.470907256	1.415605187
N	-4.722792336	-0.220414408	0.875202876
C	-6.507888754	-3.057174786	2.557503572
C	-6.412259122	-1.824324679	1.890981284
C	-5.336539909	-3.788215607	2.878757511
H	-7.321715236	-1.296837913	1.629300800
C	-5.149616763	-1.358063464	1.568170109
C	-4.055041348	-3.343285786	2.544269460
H	-3.176264191	-3.933658842	2.769364997
C	-4.015831367	-2.129360642	1.892137575
C	-8.992728859	-5.553664553	3.967573843
C	-8.960598928	-6.776021420	4.619281389
C	-7.802573224	-4.911172804	3.587925657
H	-9.884528871	-7.267981067	4.906720713
C	-7.725164473	-7.381139696	4.904419512
C	-6.582471161	-5.535815511	3.880428497
H	-7.695896527	-8.341746983	5.410572432
C	-6.535206877	-6.768134257	4.539170528
H	-5.569569067	-7.220534676	4.737531349
H	-9.926335674	-5.056836871	3.723736126
C	-7.835171461	-3.629841343	2.854738970
O	-5.379522392	-4.999879037	3.517226023
O	-8.888404252	-3.100903271	2.497488513
H	-4.768981164	-0.725150592	-3.274000563
H	-5.234761712	-0.201849313	-1.648606147
O	-2.213147841	-3.162496132	-1.025357451

C	-4.121548695	-8.582598348	-2.354602498
H	-4.823977376	-9.347591725	-2.672840577
C	-2.758460968	-8.798109163	-2.448917781
C	-4.641413193	-7.361234481	-1.839958526
C	-1.855875551	-7.800627636	-2.040702577
H	-2.384411682	-9.738494110	-2.843127936
C	-3.698242642	-6.365183101	-1.435189247
C	-6.011270170	-7.088996561	-1.737776064
C	-2.326773418	-6.585828495	-1.539042837
H	-0.786166305	-7.968063015	-2.122922152
H	-1.642786162	-5.793631844	-1.248816411
N	-4.172045771	-5.134382873	-0.980234175
C	-5.478641912	-4.760588152	-0.947561672
H	-3.473494275	-4.377916451	-0.861453214
C	-6.540003420	-5.857529202	-1.088217923
H	-6.746817967	-7.808880882	-2.081950462
O	-7.636790122	-5.365169783	-1.854394022
O	-5.809289885	-3.583520276	-0.750285588
C	-8.670307630	-4.914156733	-0.969462269
C	-8.646724923	-5.922369123	0.174047076
H	-9.604277021	-4.902214255	-1.538285633
H	-8.450330197	-3.902242747	-0.606494938
C	-7.138469915	-6.120952603	0.403091908
H	-9.108653193	-6.866191245	-0.138821568
H	-9.152821849	-5.563203936	1.073290870
C	-6.739121584	-7.403841403	1.020941611
H	-6.767415176	-5.291433388	1.009252370
H	-7.292831891	-8.313903818	0.814171984
H	-5.802751969	-7.491538599	1.559905243

==============================
os-syn-8m
==============================

C	-1.503508000	-0.977638000	-1.791929000
C	-2.036537000	0.021109000	-2.834988000
C	-3.361939000	0.637591000	-2.359840000
C	-3.130980000	1.372494000	-1.012735000
C	-2.443490000	0.563207000	0.114747000
C	-1.233583000	-0.221368000	-0.461401000
H	-2.183483000	-0.485656000	-3.798043000
H	-1.288103000	0.807715000	-3.000150000
H	-4.078746000	1.775163000	-0.641415000
H	-2.480354000	2.231155000	-1.224178000
H	-0.847681000	-0.920864000	0.285014000
H	-0.439142000	0.508637000	-0.661625000
C	-0.208620000	-1.642279000	-2.276550000
C	-3.885577000	1.662071000	-3.378918000
C	-2.552907000	-2.061201000	-1.505519000
C	-4.390673000	-0.507776000	-2.262786000
N	-3.846028000	-1.744378000	-1.702359000
H	-4.541257000	-2.419764000	-1.357576000
H	0.569185000	-0.886681000	-2.431229000
H	0.142917000	-2.374787000	-1.547474000
H	-0.371183000	-2.164237000	-3.225400000
H	-4.006615000	1.207290000	-4.368240000
H	-3.191960000	2.503792000	-3.478621000
H	-4.857676000	2.063069000	-3.069773000
C	-1.938771000	1.530929000	1.215167000
H	-1.201793000	2.226643000	0.800472000
H	-1.466422000	0.975788000	2.031642000
H	-2.769938000	2.110257000	1.628075000
C	-3.439703000	-0.350464000	0.784994000
O	-2.920364000	-1.472341000	1.413451000
N	-4.722384000	-0.218320000	0.880747000
C	-6.506821000	-3.054625000	2.564169000
C	-6.411137000	-1.820719000	1.899741000
C	-5.335670000	-3.788114000	2.880425000
H	-7.320509000	-1.291306000	1.641613000
C	-5.148723000	-1.356095000	1.573750000
C	-4.054387000	-3.344498000	2.543267000
H	-3.175964000	-3.936887000	2.764463000
C	-4.015160000	-2.129643000	1.892941000
C	-8.991943000	-5.556275000	3.964454000
C	-8.959969000	-6.781980000	4.609909000
C	-7.801739000	-4.912013000	3.588184000
H	-9.883971000	-7.275121000	4.895123000
C	-7.724667000	-7.388928000	4.891597000
C	-6.581697000	-5.538780000	3.876595000
H	-7.695530000	-8.352085000	5.392932000
C	-6.534613000	-6.774608000	4.528761000
H	-5.569053000	-7.228473000	4.724139000
H	-9.925477000	-5.057935000	3.723496000
C	-7.834181000	-3.626775000	2.862005000
O	-5.378748000	-5.001415000	3.515636000
O	-8.887527000	-3.094729000	2.509554000
H	-4.776343000	-0.726129000	-3.267971000
H	-5.238500000	-0.203955000	-1.641136000
O	-2.209008000	-3.161938000	-1.034009000
C	-4.117168000	-8.602681000	-2.321201000
H	-4.821074000	-9.377994000	-2.611329000
C	-2.754543000	-8.808489000	-2.445594000
C	-4.634567000	-7.378041000	-1.811717000
C	-1.850175000	-7.798318000	-2.072749000
H	-2.382528000	-9.751304000	-2.835922000
C	-3.689603000	-6.368813000	-1.444141000
C	-6.004370000	-7.119086000	-1.674818000
C	-2.319009000	-6.580188000	-1.576639000
H	-0.781072000	-7.958553000	-2.177432000
H	-1.634560000	-5.779238000	-1.311282000
N	-4.161882000	-5.137413000	-0.988820000
C	-5.468400000	-4.768052000	-0.933540000
H	-3.463792000	-4.379428000	-0.875751000
C	-6.534873000	-5.862809000	-1.064518000
H	-6.741334000	-7.852921000	-1.986466000
O	-7.610606000	-5.376144000	-1.868806000
O	-5.798172000	-3.591422000	-0.730048000

C	-8.654443000	-4.892827000	-1.011228000
C	-8.663993000	-5.876775000	0.155303000
H	-9.578483000	-4.881064000	-1.596420000
H	-8.426767000	-3.876011000	-0.666879000
C	-7.161936000	-6.102023000	0.402120000
H	-9.142475000	-6.817369000	-0.142854000
H	-9.173078000	-5.487256000	1.040879000
C	-6.790482000	-7.401477000	1.015254000
H	-6.784485000	-5.284143000	1.020760000
H	-7.405602000	-8.285913000	0.861776000
H	-5.832206000	-7.524664000	1.510465000

==============================
syn-9m
==============================

C	-1.507753495	-0.881368895	-1.843387942
C	-2.028068637	0.181444839	-2.827329577
C	-3.338287234	0.794236093	-2.308011751
C	-3.085100150	1.451228224	-0.924804771
C	-2.397915745	0.573743446	0.149883265
C	-1.207628084	-0.197908866	-0.480585925
H	-2.191942117	-0.268963298	-3.815264977
H	-1.266109929	0.962115177	-2.955432633
H	-4.024145526	1.845264759	-0.523117433
H	-2.425044564	2.311833183	-1.095069354
H	-0.822896067	-0.939393021	0.224614831
H	-0.403950748	0.528819423	-0.654971032
C	-0.228449993	-1.543883641	-2.371590339
C	-3.848992145	1.884117951	-3.264387183
C	-2.576232911	-1.961912567	-1.609661921
C	-4.387665263	-0.336867709	-2.270105888
N	-3.862847723	-1.618691234	-1.800800568
H	-4.568866969	-2.315142721	-1.522181610
H	0.563438088	-0.796269770	-2.490565069
H	0.112675547	-2.320484755	-1.684314464
H	-0.405625231	-2.011508903	-3.345724684
H	-3.981301874	1.489126510	-4.277506588
H	-3.141742893	2.718559924	-3.319087135
H	-4.813019381	2.282962855	-2.928351867
C	-1.869961644	1.475641840	1.295686274
H	-1.128209324	2.183159544	0.910463680
H	-1.396709781	0.873662658	2.077810961
H	-2.689715265	2.043433560	1.746421189
C	-3.398936611	-0.362422639	0.778776661
O	-2.881811018	-1.512488424	1.356515275
N	-4.680222689	-0.228097654	0.887326514
C	-6.463538810	-3.100203067	2.511749442
C	-6.368440268	-1.849991825	1.877385935
C	-5.293893611	-3.854668145	2.781860115
H	-7.276570370	-1.302209220	1.656181087
C	-5.107553020	-1.387871389	1.542144822
C	-4.014759488	-3.413181036	2.434936015
H	-3.137704359	-4.018122040	2.625307654
C	-3.975675482	-2.179524742	1.821577204
C	-8.941149365	-5.596095258	3.935187627
C	-8.908931826	-6.845624805	4.534007124
C	-7.753517831	-4.960967515	3.537592631
H	-9.830988371	-7.331591175	4.836798498
C	-7.676532350	-7.486261707	4.743597846
C	-6.534795766	-5.618285542	3.760982621
H	-7.647916461	-8.467829261	5.207660210
C	-6.487878978	-6.880361686	4.360432699
H	-5.524495123	-7.357180919	4.505308555
H	-9.872912727	-5.072741590	3.746181789
C	-7.789041581	-3.654255443	2.848233182
O	-5.335651878	-5.086122726	3.381533103
O	-8.845070368	-3.092815736	2.555904474
H	-4.788478342	-0.480661176	-3.282773627
H	-5.222808097	-0.057897212	-1.620022921
O	-2.251328648	-3.090223745	-1.193991309
C	-4.114098397	-8.707547693	-1.832106773
H	-4.793939950	-9.547604414	-1.954175885
C	-2.741938132	-8.891923895	-2.003506718
C	-4.643119371	-7.458392826	-1.497299821
C	-1.876114597	-7.807266065	-1.846033782
H	-2.351614827	-9.872294553	-2.260118605
C	-3.758964298	-6.376022106	-1.354181526
C	-6.109144867	-7.254599650	-1.245553792
C	-2.379645424	-6.549441479	-1.526816529
H	-0.806024485	-7.937126971	-1.981244338
H	-1.724496946	-5.688753761	-1.424147350
N	-4.233141730	-5.084720453	-1.054023897
C	-5.530389481	-4.691058124	-1.065511835
H	-3.526228373	-4.330864315	-1.007774176
C	-6.591503823	-5.778379969	-1.109130727
H	-6.712670047	-7.767825157	-1.999205558
O	-7.647635994	-5.422986362	-1.978281489
O	-5.855576131	-3.497610916	-0.979405706
C	-8.729155570	-4.953201614	-1.155139857
C	-8.715851732	-5.879105833	0.071508128
H	-9.644675931	-5.010986727	-1.750389067
H	-8.550743969	-3.911235973	-0.860442570
C	-7.209064914	-6.098741165	0.287903802
H	-9.215262735	-6.825634572	-0.162907251
H	-9.206759024	-5.430211770	0.938784562
C	-6.618786242	-7.532037459	0.212789082
H	-6.821772382	-5.492624741	1.101396838
H	-7.336675050	-8.350461607	0.316970407
H	-5.799496746	-7.694950549	0.918073290

Computed Vibrational Frequencies

==============================
syn-6 (conformer1/12)
==============================

22.48 23.01 28.54 40.53 42.75 48.08 55.08 63.29 72.98
79.63 83.94 88.67 89.28 101.27 115.11 118.79 123.60
126.45 143.79 149.27 161.85 182.23 188.67 201.63 217.84
224.83 240.66 241.44 243.73 247.63 253.78 260.14 261.95
267.76 276.90 280.92 301.85 306.01 314.75 320.36 323.05
335.73 345.90 349.43 356.81 380.25 393.13 397.21 420.65
445.51 448.96 449.58 451.09 462.08 465.41 468.95 488.54
496.61 504.20 507.26 512.67 528.59 530.78 555.67 570.99
576.62 591.82 595.58 608.22 619.81 621.50 646.72 650.20
665.05 681.49 685.18 696.92 706.09 710.48 717.92 729.85
733.05 752.47 755.93 761.25 771.64 777.32 778.03 780.89
797.13 798.59 816.21 843.35 845.85 881.31 883.09 889.19
898.18 899.89 903.48 907.42 914.73 923.91 928.29 938.05
952.69 956.70 960.88 963.43 967.32 970.07 972.00 981.74
989.06 1005.76 1010.56 1023.17 1025.93 1031.17 1031.55
1043.13 1055.85 1066.84 1078.57 1084.29 1088.49 1110.43
1117.75 1118.34 1133.87 1138.48 1140.51 1144.35 1153.86
1167.68 1176.17 1185.92 1187.88 1198.21 1198.75 1199.68
1220.16 1240.62 1250.96 1255.21 1258.08 1266.80 1267.66
1273.50 1289.17 1292.59 1306.54 1314.97 1320.14 1323.89
1334.06 1339.69 1343.41 1351.52 1354.60 1355.44 1358.33
1360.68 1364.61 1381.48 1388.43 1394.08 1402.96 1408.90
1428.66 1434.33 1437.80 1443.86 1445.44 1446.66 1450.84
1466.67 1479.82 1493.90 1500.36 1504.47 1509.30 1512.11
1514.49 1517.07 1518.01 1519.68 1520.68 1523.61 1528.61
1531.56 1538.37 1539.74 1543.88 1544.84 1555.04 1568.05
1582.90 1599.71 1615.32 1667.04 1669.66 1697.26 1709.41
1716.53 1730.60 1750.45 1767.63 1787.32 3036.36 3054.82
3057.64 3062.20 3067.90 3068.93 3071.92 3075.63 3081.16
3101.83 3103.53 3122.21 3124.87 3135.33 3140.53 3141.30
3144.30 3145.06 3150.58 3161.95 3167.07 3177.37 3178.56
3195.69 3206.94 3215.19 3218.00 3226.29 3231.69 3235.80
3244.16 3248.26 3249.37 3260.91 3287.55 3291.81 3292.27

==============================
syn-7
==============================
17.34 26.26 29.88 32.50 38.83 51.67 54.20 60.63 64.69
74.09 78.36 83.95 89.22 110.66 112.96 115.46 123.40
127.40 131.58 153.93 158.47 182.02 188.23 189.03 202.74
229.09 233.41 243.10 244.75 248.42 256.65 257.15 259.16
264.04 276.21 288.68 293.37 301.94 312.36 326.03 334.01
338.55 343.24 358.37 381.83 388.13 395.39 415.29 420.31
434.69 438.35 440.50 444.72 449.37 455.38 468.73 475.85
490.71 510.10 516.06 522.40 534.53 537.78 554.65 581.79
595.07 602.62 607.33 618.54 627.67 630.94 653.98 664.69
665.86 669.81 688.06 710.93 714.02 727.91 731.85 741.07
747.42 758.83 760.90 763.52 770.26 782.76 792.40 811.02
814.69 822.88 836.31 843.43 851.55 855.56 861.62 871.13
888.77 896.12 903.27 905.39 912.27 921.72 923.30 937.47
944.82 954.61 957.29 959.41 964.89 971.45 973.07 975.93
984.80 1003.93 1011.87 1024.10 1025.62 1029.33 1030.53
1044.24 1050.16 1057.12 1063.83 1070.88 1099.93 1104.54
1120.66 1131.01 1136.43 1139.14 1142.01 1147.48 1167.52
1175.96 1177.17 1184.85 1192.59 1193.26 1194.28 1213.51
1222.36 1226.76 1243.18 1256.54 1261.09 1265.19 1276.90
1282.10 1292.88 1297.43 1309.07 1317.91 1319.44 1325.41
1330.55 1332.14 1335.44 1338.40 1352.15 1359.29 1360.18
1363.27 1369.22 1388.21 1389.89 1401.19 1409.63 1429.46
1431.81 1435.65 1438.05 1444.52 1445.00 1448.14 1476.38
1484.60 1490.66 1496.76 1502.10 1512.40 1513.63 1517.02
1518.28 1518.54 1520.21 1523.08 1527.31 1528.27 1531.70
1533.19 1535.80 1539.50 1544.29 1550.98 1557.28 1572.38
1587.46 1616.69 1629.38 1661.95 1678.33 1700.20 1707.52
1727.43 1731.40 1766.44 1769.44 1789.81 3051.93 3055.12
3060.22 3066.00 3066.32 3069.38 3072.32 3076.48 3079.30
3097.41 3123.43 3126.98 3130.18 3140.68 3144.42 3144.63
3144.86 3145.74 3152.61 3158.19 3164.06 3177.79 3178.02
3206.49 3215.76 3216.06 3224.15 3229.60 3237.96 3242.80
3251.85 3261.32 3261.67 3263.38 3272.44 3281.44 3337.10

==============================
anti-6 (conformer1/5)
==============================
15.42 20.72 27.96 33.28 39.92 53.37 58.64 62.99 64.57
69.52 80.50 95.56 99.34 109.53 117.98 122.01 127.78
133.34 149.26 153.10 167.71 178.01 193.22 210.24 218.44
231.14 237.48 241.22 245.63 247.37 247.94 259.50 263.78
264.89 274.20 276.32 299.29 306.20 311.35 316.39 320.55
340.29 343.29 350.45 360.82 377.65 396.25 399.78 419.43
443.93 446.08 449.94 450.74 461.10 464.88 470.36 489.35
490.01 498.11 505.30 514.89 530.32 533.29 555.58 564.79
572.92 589.49 595.43 600.08 621.31 623.41 636.29 647.70
660.22 679.69 681.03 694.81 706.12 710.58 717.14 728.83
733.21 758.49 760.71 762.82 773.41 777.80 777.96 781.22
794.69 802.05 818.38 840.73 843.27 879.02 883.86 891.16
894.99 903.29 903.68 906.64 916.80 920.18 928.45 937.48
948.10 954.51 955.33 959.39 967.14 968.85 974.49 974.67
993.39 1008.87 1016.79 1020.73 1020.92 1029.77 1032.07
1041.74 1061.04 1065.87 1078.41 1089.70 1091.95 1110.94
1112.49 1114.16 1134.13 1137.12 1140.56 1142.68 1152.77
1168.64 1173.49 1185.07 1187.14 1195.80 1197.51 1198.36
1222.12 1237.64 1249.72 1252.91 1258.82 1264.43 1270.09
1271.20 1282.45 1287.07 1302.05 1312.46 1322.62 1324.46
1329.59 1333.70 1340.26 1346.03 1350.74 1353.80 1355.01
1357.27 1360.53 1379.63 1388.26 1393.56 1407.11 1408.95
1431.29 1435.66 1440.37 1443.20 1445.16 1448.56 1464.01
1476.88 1478.05 1486.30 1496.13 1501.91 1504.52 1511.32
1512.04 1514.06 1515.65 1517.04 1522.58 1523.38 1527.63
1528.89 1536.83 1540.49 1546.48 1548.87 1550.56 1567.58
1584.71 1597.43 1612.65 1670.11 1670.57 1697.07 1712.14
1719.22 1752.35 1758.23 1769.13 1785.16 3042.23 3052.94
3055.14 3062.54 3065.64 3067.58 3071.73 3076.46 3081.39
3095.44 3099.86 3119.39 3124.42 3135.80 3140.25 3144.59
3145.22 3146.23 3147.85 3160.18 3167.69 3179.23 3182.11
3198.26 3206.48 3214.98 3215.29 3227.75 3229.27 3236.72
3243.13 3243.44 3246.46 3263.37 3288.86 3294.68 3321.05

==============================
anti-7
==============================
13.45 23.68 28.54 34.12 41.06 56.36 58.35 60.66 63.88
74.08 80.54 93.29 94.57 107.99 114.86 117.38 125.89
134.55 137.43 150.17 171.85 187.77 190.09 195.81 199.38
225.65 235.24 241.95 242.97 246.37 253.60 257.59 259.64
262.75 274.63 283.26 289.62 304.37 312.84 323.82 337.99

341.14 343.45 357.82 381.94 387.49 395.26 414.07 421.24
430.85 439.50 440.49 444.36 446.51 452.09 469.51 478.37
491.98 510.42 516.42 524.02 530.48 540.02 552.92 572.69
596.47 600.01 608.37 617.80 630.35 631.11 654.24 663.65
666.37 671.76 685.38 713.79 714.05 728.70 729.73 741.39
746.91 754.42 759.38 762.17 770.47 782.92 786.14 791.40
809.69 824.48 835.43 844.33 850.74 855.04 863.31 869.58
892.01 897.49 902.49 904.35 912.12 922.65 925.03 939.12
941.12 956.30 957.32 961.21 964.32 971.05 974.44 977.37
981.67 1002.12 1019.40 1021.72 1024.59 1025.71 1034.80
1044.33 1052.20 1059.09 1066.84 1069.94 1098.11 1108.84
1116.35 1134.45 1136.29 1137.64 1142.17 1148.00 1166.27
1175.10 1175.58 1181.60 1191.36 1192.36 1195.59 1212.63
1223.99 1225.07 1241.51 1253.47 1259.58 1263.65 1274.57
1280.93 1288.35 1298.05 1308.85 1312.68 1321.12 1323.59
1327.44 1329.23 1335.82 1336.41 1346.95 1352.60 1356.10
1357.43 1367.31 1383.97 1387.42 1400.09 1408.58 1427.23
1432.07 1438.61 1443.35 1444.43 1449.11 1449.58 1476.10
1483.27 1488.05 1497.68 1501.76 1510.63 1511.99 1512.58
1516.51 1517.77 1521.05 1523.82 1526.84 1526.98 1528.81
1536.34 1539.80 1544.27 1546.67 1548.49 1556.43 1569.58
1590.03 1618.61 1628.90 1660.96 1677.06 1699.58 1705.01
1723.14 1729.42 1769.19 1773.77 1800.42 3050.25 3053.53
3062.38 3066.55 3067.83 3069.66 3072.42 3076.36 3081.02
3098.50 3121.27 3123.63 3124.44 3139.68 3142.37 3144.08
3145.76 3147.15 3148.80 3164.86 3168.87 3180.19 3183.12
3209.61 3214.26 3218.87 3227.30 3230.11 3238.80 3242.34
3246.06 3263.89 3264.44 3267.63 3271.88 3290.52 3344.57

==============================
syn-6 (conformer2/12)
==============================
21.68 22.80 28.25 39.62 42.10 48.09 54.92 63.07 72.44
79.50 83.84 88.22 88.95 101.34 114.77 118.63 123.57
125.94 143.60 148.85 162.07 182.15 188.74 201.92 217.67
224.62 240.41 241.72 244.16 247.44 253.47 260.03 262.06
267.02 276.48 280.85 302.39 305.92 314.78 320.40 323.05
335.90 345.70 348.42 356.86 380.20 393.20 397.22 420.60
445.43 448.96 449.18 450.98 461.72 465.18 468.94 488.62
495.28 504.39 507.30 512.65 528.69 530.74 555.65 570.95
576.03 591.70 595.56 608.60 619.88 621.42 646.61 650.12
665.11 681.42 685.06 696.93 706.02 710.56 717.92 729.45
733.08 752.20 755.73 760.71 771.59 777.22 777.95 780.93
797.28 798.60 815.95 843.31 845.75 880.87 882.42 889.06
897.75 900.47 903.24 907.00 914.52 924.10 928.21 938.20
952.65 956.87 961.02 963.53 967.32 969.98 971.97 981.65
989.46 1005.92 1010.72 1023.32 1025.59 1031.37 1031.62
1043.19 1055.78 1066.86 1078.51 1084.31 1088.29 1110.36
1117.46 1117.94 1133.74 1138.42 1140.51 1144.32 1153.77
1167.67 1175.94 1185.96 1187.85 1198.17 1198.76 1199.66
1219.78 1240.46 1250.75 1255.19 1257.99 1266.85 1267.70
1273.55 1289.11 1292.42 1306.59 1315.01 1320.11 1323.92
1334.07 1339.79 1343.51 1351.53 1354.52 1355.57 1358.34
1360.81 1364.51 1381.52 1388.16 1393.75 1402.90 1408.60
1428.86 1434.76 1437.85 1443.79 1445.48 1446.70 1450.74
1466.68 1479.83 1493.99 1500.25 1504.45 1509.15 1512.05
1514.40 1516.95 1518.06 1519.66 1520.69 1523.94 1528.75
1531.58 1538.21 1539.71 1544.00 1544.83 1555.18 1567.93
1582.81 1599.57 1615.51 1666.90 1669.72 1697.33 1709.31
1716.71 1730.19 1750.48 1767.62 1787.31 3036.44 3054.66
3057.51 3062.18 3067.84 3068.86 3072.03 3075.61 3081.19
3101.73 3103.74 3122.10 3124.70 3135.39 3140.57 3141.27
3144.53 3145.18 3150.63 3161.95 3167.02 3177.56 3178.50
3193.14 3206.96 3215.21 3217.99 3226.35 3231.70 3235.86
3244.27 3248.27 3249.45 3260.88 3286.22 3291.71 3292.30

==============================
syn-6 (conformer3/12)
==============================
21.20 22.74 28.06 39.06 41.82 48.22 54.88 62.86 72.38
79.35 83.74 88.01 88.71 101.46 114.63 118.52 123.52
125.77 143.43 148.69 162.16 182.11 188.73 201.89 217.63
224.58 240.21 241.63 244.59 246.95 253.36 259.98 262.22
266.79 275.96 280.75 302.35 305.67 314.57 320.45 323.01
335.87 345.57 348.06 356.73 380.14 393.13 397.08 420.60
445.37 448.83 449.01 450.93 461.55 465.21 468.90 488.58
494.71 504.30 506.99 512.60 528.59 530.71 555.58 570.92
575.71 591.60 595.55 608.68 619.87 621.38 646.55 650.06
665.08 681.46 685.06 696.97 705.97 710.57 717.92 729.34
733.03 752.07 755.56 760.43 771.58 777.23 777.97 781.00
797.34 798.63 815.83 843.17 845.71 880.78 882.11 888.83
897.41 900.79 903.08 906.88 914.48 924.24 928.15 938.31
952.55 956.81 960.97 963.21 967.38 970.04 971.66 981.29
989.69 1005.95 1010.98 1023.37 1025.29 1031.47 1031.66
1043.22 1055.69 1066.96 1078.50 1084.26 1088.07 1110.29
1117.20 1117.50 1133.71 1138.21 1140.44 1144.28 1153.74
1167.65 1175.82 1185.98 1187.76 1198.16 1198.70 1199.64
1219.51 1240.37 1250.64 1255.09 1258.01 1266.87 1267.72
1273.47 1289.06 1292.36 1306.52 1315.05 1320.10 1323.93
1333.91 1339.79 1343.39 1351.37 1354.49 1355.62 1358.30
1360.79 1364.41 1381.57 1388.03 1393.57 1402.75 1408.41
1429.35 1435.18 1437.85 1443.96 1445.44 1446.69 1451.14
1466.69 1479.80 1494.06 1500.05 1504.53 1508.96 1512.04
1514.33 1516.87 1518.03 1519.63 1520.72 1524.02 1528.75
1531.54 1538.09 1539.65 1544.03 1544.93 1555.17 1567.85
1582.89 1599.18 1615.42 1667.15 1669.57 1697.46 1709.26
1716.54 1730.65 1750.68 1767.59 1787.16 3036.54 3054.48
3057.36 3062.15 3067.79 3068.83 3072.08 3075.59 3081.28
3101.53 3103.83 3122.01 3124.62 3135.47 3140.68 3141.23
3144.59 3145.17 3150.61 3161.87 3167.03 3177.76 3178.51
3196.23 3206.93 3215.12 3217.95 3226.20 3231.67 3235.80
3244.33 3248.32 3249.53 3260.75 3286.00 3291.62 3292.15

==============================
syn-6 (conformer4/12)
==============================
20.55 22.69 27.86 38.24 41.46 48.25 54.70 62.71 71.79
79.25 83.59 87.77 88.46 101.58 114.36 118.41 123.40
125.36 143.27 148.44 162.27 182.00 188.73 201.99 217.30
224.39 239.78 241.43 245.55 246.64 253.28 259.89 262.39
266.33 275.49 280.66 302.69 305.64 314.52 320.48 323.00
335.92 345.25 347.47 356.74 380.08 393.19 397.04 420.61
445.31 448.38 448.94 450.88 461.41 465.14 468.92 488.55
493.64 504.30 506.74 512.51 528.65 530.69 555.48 570.92
575.19 591.50 595.52 608.54 619.85 621.33 646.47 649.98
664.91 681.43 685.01 696.97 705.86 710.62 717.92 729.30

733.01 751.90 755.43 760.01 771.56 777.14 777.87 780.98
797.47 798.76 815.91 843.11 845.65 880.94 881.83 888.83
897.43 901.31 902.97 906.69 914.55 924.43 928.13 938.46
952.53 956.90 961.04 963.37 967.33 969.96 971.71 981.27
990.01 1006.03 1011.30 1023.50 1025.03 1031.65 1031.70
1043.27 1055.67 1067.01 1078.44 1084.40 1088.00 1110.26
1117.07 1117.12 1133.61 1138.06 1140.47 1144.30 1153.67
1167.65 1175.67 1185.99 1187.78 1198.13 1198.75 1199.59
1219.13 1240.35 1250.55 1255.04 1257.84 1266.87 1267.71
1273.50 1288.96 1292.29 1306.48 1315.08 1320.03 1323.97
1333.79 1339.85 1343.40 1351.37 1354.28 1355.70 1358.25
1360.75 1364.35 1381.49 1387.88 1393.31 1402.66 1408.20
1429.84 1435.71 1437.95 1443.99 1445.42 1446.73 1451.40
1466.78 1479.87 1493.96 1499.71 1504.52 1509.00 1512.02
1514.23 1516.71 1517.98 1519.65 1520.82 1524.24 1528.81
1531.51 1538.01 1539.54 1544.05 1544.96 1555.11 1567.74
1582.84 1599.18 1615.30 1667.11 1669.60 1697.51 1709.23
1716.69 1730.79 1750.65 1767.56 1787.21 3036.50 3054.38
3057.35 3062.19 3067.81 3068.76 3072.14 3075.59 3081.21
3101.50 3103.96 3121.97 3124.56 3135.46 3140.74 3141.38
3144.69 3145.23 3150.60 3161.76 3166.97 3177.89 3178.45
3194.92 3206.98 3215.19 3217.98 3226.34 3231.71 3235.93
3244.40 3248.24 3249.59 3260.80 3285.66 3291.68 3292.26

==============================
syn-6 (conformer5/12)
==============================
20.69 22.49 27.93 38.35 41.62 48.07 54.56 62.69 71.76
79.23 83.68 87.50 88.50 101.00 114.32 118.36 123.33
125.37 143.13 148.34 161.90 181.99 188.61 201.56 217.52
224.47 239.99 241.50 244.79 246.90 253.19 259.82 262.17
266.43 275.76 280.60 302.18 305.45 314.29 320.37 322.91
335.81 345.37 347.60 356.64 380.11 393.02 397.07 420.53
445.21 448.76 449.11 450.85 461.20 465.06 468.81 488.54
493.97 504.10 506.66 512.58 528.57 530.70 555.46 570.92
575.57 591.57 595.57 608.75 619.88 621.40 646.56 650.05
665.05 681.45 685.12 696.98 705.86 710.40 717.90 729.41
733.02 751.89 755.50 760.29 771.53 777.24 777.81 780.98
797.46 798.75 815.98 843.13 845.66 881.06 881.31 888.78
896.85 901.10 903.01 906.28 914.65 924.28 928.17 938.43
952.57 956.76 960.96 963.15 967.43 970.09 971.50 981.04
989.87 1006.14 1011.05 1023.48 1024.99 1031.47 1031.77
1043.29 1055.71 1066.96 1078.51 1084.45 1088.10 1110.33
1116.98 1117.43 1133.61 1138.19 1140.47 1144.34 1153.72
1167.65 1175.78 1185.98 1187.75 1198.16 1198.75 1199.70
1219.46 1240.47 1250.63 1255.11 1258.07 1266.86 1267.66
1273.49 1289.06 1292.35 1306.47 1315.05 1320.10 1323.95
1333.92 1339.79 1343.38 1351.43 1354.62 1355.65 1358.43
1360.74 1364.39 1381.54 1387.96 1393.59 1402.81 1408.33
1429.43 1435.22 1437.58 1443.81 1445.46 1446.60 1450.93
1466.65 1479.78 1494.12 1500.19 1504.47 1509.15 1512.11
1514.28 1516.85 1518.09 1519.60 1520.63 1524.15 1528.73
1531.58 1538.14 1539.78 1544.04 1544.92 1555.03 1567.84
1582.88 1599.43 1615.24 1667.15 1669.62 1697.44 1709.22
1716.54 1730.60 1750.66 1767.64 1787.32 3036.38 3054.52
3057.37 3062.08 3067.73 3068.91 3072.08 3075.63 3081.24
3101.54 3103.65 3121.89 3124.51 3135.42 3140.63 3141.15
3144.65 3145.14 3150.67 3161.89 3166.95 3177.78 3178.41
3195.05 3206.79 3215.02 3218.03 3226.09 3231.74 3235.72
3244.39 3248.09 3249.62 3260.72 3286.47 3291.61 3292.15

==============================
syn-6 (conformer6/12)
==============================
18.03 22.62 26.96 35.16 40.45 48.20 53.91 61.80 69.67
78.82 82.94 86.64 87.77 101.89 113.50 117.92 122.82
124.42 142.40 147.64 162.45 181.56 188.62 200.80 216.53
223.84 236.43 240.65 245.07 249.25 253.13 259.42 263.08
265.35 273.84 280.20 302.57 305.68 313.88 320.42 322.88
335.80 343.53 346.32 356.48 379.76 392.76 396.45 420.87
445.49 446.50 448.68 450.71 460.54 465.00 468.82 488.02
489.94 504.30 506.11 512.15 528.56 530.66 554.96 570.83
573.74 591.01 595.39 609.02 619.88 621.13 645.96 649.48
664.75 681.28 684.92 697.01 705.35 710.84 717.86 728.51
733.04 751.36 755.03 759.05 771.42 777.22 777.52 781.09
797.44 799.42 815.54 842.64 845.16 880.12 880.40 888.10
897.34 902.69 903.22 905.42 914.32 924.78 928.01 938.87
952.30 956.65 961.20 962.76 967.40 969.97 970.82 980.14
991.31 1006.45 1012.95 1023.13 1024.08 1031.69 1032.10
1043.31 1055.54 1067.17 1078.24 1084.61 1087.86 1110.33
1115.36 1116.15 1133.50 1137.57 1140.35 1144.26 1153.64
1167.58 1175.00 1186.11 1187.57 1197.99 1198.70 1199.53
1217.59 1239.95 1250.04 1254.50 1257.33 1266.91 1267.74
1273.41 1288.68 1291.91 1306.39 1315.09 1319.83 1323.99
1333.22 1339.94 1343.41 1351.01 1353.54 1355.65 1358.26
1360.92 1364.02 1381.56 1387.43 1392.48 1402.31 1407.34
1431.95 1436.69 1438.15 1443.56 1445.60 1446.60 1451.74
1466.88 1480.03 1493.98 1498.52 1504.40 1508.43 1511.99
1513.52 1516.42 1517.74 1519.40 1520.89 1525.05 1529.01
1531.28 1537.36 1539.36 1544.11 1545.02 1555.23 1567.39
1583.04 1598.80 1615.42 1667.17 1669.51 1697.70 1708.99
1716.80 1730.75 1751.18 1767.58 1787.13 3036.51 3053.61
3056.95 3062.13 3067.60 3068.50 3072.37 3075.58 3081.07
3100.87 3103.80 3121.62 3124.22 3135.40 3141.02 3141.54
3145.00 3145.53 3150.54 3161.37 3166.88 3178.39 3178.66
3196.95 3206.99 3215.10 3218.00 3226.17 3231.80 3236.01
3244.85 3248.37 3250.03 3260.65 3284.71 3291.56 3292.22

==============================
syn-6 (conformer7/12)
==============================
19.98 22.18 28.97 34.53 42.19 47.72 55.70 63.51 75.00
78.09 84.89 86.42 88.20 101.97 115.18 117.18 120.51
131.17 144.74 150.13 162.32 181.86 189.87 203.51 217.79
224.97 239.97 241.40 244.18 247.64 253.77 260.35 261.99
271.32 272.66 278.44 300.49 304.31 313.54 318.51 322.26
334.83 344.77 346.95 354.55 380.96 392.81 396.82 421.38
445.90 449.86 450.89 452.17 461.89 468.29 474.66 488.37
490.72 502.92 511.34 515.42 529.29 530.99 556.25 569.57
575.66 593.58 595.74 610.88 619.67 623.36 645.39 648.67
664.95 680.66 685.35 696.30 703.75 708.31 717.40 731.94
732.33 750.73 755.80 759.65 771.18 775.13 777.88 779.40
795.92 802.06 817.39 843.35 843.50 876.78 883.35 890.12
898.66 900.07 902.87 904.98 914.20 923.83 926.87 937.95
951.72 956.46 960.61 962.44 967.11 969.78 970.18 978.59
988.24 1004.25 1010.35 1023.22 1026.21 1030.78 1031.27

1043.21 1055.55 1066.68 1075.21 1081.80 1089.11 1110.04
1111.64 1118.99 1129.47 1138.51 1140.93 1144.75 1153.62
1165.75 1176.75 1185.62 1190.62 1196.85 1198.31 1198.93
1221.11 1239.31 1250.03 1254.77 1257.63 1266.02 1267.28
1273.92 1288.59 1293.33 1305.42 1315.55 1320.21 1324.28
1334.84 1338.64 1342.67 1345.26 1354.80 1355.29 1358.19
1360.75 1374.92 1378.73 1388.86 1394.33 1397.28 1409.62
1422.50 1428.47 1433.54 1436.20 1442.90 1444.10 1446.28
1463.29 1477.89 1494.17 1501.31 1504.19 1510.21 1513.78
1514.95 1516.24 1517.88 1520.18 1521.33 1523.55 1527.12
1532.01 1534.67 1541.27 1542.95 1548.78 1562.63 1568.75
1578.62 1592.31 1632.42 1647.00 1665.37 1676.20 1695.25
1712.18 1732.27 1749.13 1767.64 1778.00 3041.95 3054.66
3057.18 3061.71 3067.51 3069.18 3071.76 3075.65 3081.88
3101.55 3110.29 3122.57 3125.21 3136.85 3140.25 3142.29
3144.03 3144.70 3151.03 3161.61 3166.93 3177.42 3178.41
3207.45 3215.97 3216.77 3217.82 3226.22 3231.52 3236.28
3244.27 3249.91 3253.44 3260.78 3284.60 3285.74 3288.47

==============================
syn-6 (conformer8/12)
==============================
20.11 22.20 28.97 34.52 42.29 47.75 55.75 63.59 75.16
78.17 84.94 86.44 88.23 102.03 115.22 117.25 120.51
131.26 144.82 150.16 162.40 181.87 189.78 203.50 217.70
224.88 239.75 241.42 244.18 247.90 253.91 260.33 261.93
271.36 272.65 278.75 300.57 304.41 313.57 318.51 322.27
334.75 344.77 346.99 354.52 380.97 392.76 396.79 421.39
445.89 449.85 450.92 452.17 461.89 468.31 474.68 488.34
490.94 502.93 511.31 515.36 529.31 530.98 556.22 569.56
575.67 593.58 595.68 611.05 619.72 623.32 645.43 648.67
665.06 680.69 685.38 696.30 703.76 708.38 717.38 731.94
732.33 750.75 755.83 759.69 771.19 775.17 777.86 779.48
795.87 802.05 817.34 843.36 843.53 876.84 883.33 890.07
898.37 899.98 902.99 904.88 914.17 923.76 926.80 937.88
951.72 956.46 960.17 962.45 967.11 969.77 970.12 978.41
988.21 1004.27 1010.25 1023.18 1026.28 1030.72 1031.25
1043.18 1055.54 1066.63 1075.25 1081.93 1089.16 1109.99
1111.65 1119.15 1129.47 1138.65 1140.98 1144.79 1153.66
1165.76 1176.74 1185.60 1190.62 1196.84 1198.34 1198.94
1221.15 1239.28 1249.83 1254.81 1257.53 1266.06 1267.31
1273.92 1288.67 1293.24 1305.40 1315.55 1320.21 1324.24
1334.85 1338.64 1342.52 1345.29 1354.70 1355.29 1358.16
1360.78 1374.85 1378.80 1388.88 1394.33 1397.25 1409.64
1422.65 1428.23 1433.33 1436.19 1442.87 1444.09 1446.29
1463.33 1477.88 1494.14 1501.35 1504.13 1510.17 1513.77
1514.96 1516.28 1517.88 1520.16 1521.35 1523.53 1527.18
1532.00 1534.76 1541.26 1542.96 1548.69 1562.72 1568.70
1578.49 1592.35 1632.48 1646.95 1665.37 1676.20 1695.29
1712.16 1732.24 1748.94 1767.63 1777.99 3041.77 3054.73
3057.26 3061.72 3067.52 3069.18 3071.75 3075.65 3081.88
3101.63 3110.10 3122.59 3125.23 3136.82 3140.18 3142.33
3144.04 3144.72 3151.06 3161.65 3166.94 3177.31 3178.42
3207.49 3216.26 3217.56 3217.81 3226.27 3231.50 3236.30
3244.24 3249.89 3253.56 3260.80 3285.29 3286.71 3288.47

==============================
syn-6 (conformer9/12)
==============================
20.10 22.22 28.96 34.66 42.28 47.83 55.80 63.61 75.32
78.26 84.92 86.44 88.29 101.99 115.23 117.22 120.41
131.25 144.86 150.16 162.39 181.89 189.70 203.25 217.67
224.82 239.75 241.38 244.11 247.93 253.92 260.34 261.94
271.42 272.63 278.80 300.64 304.44 313.58 318.54 322.27
334.74 344.93 347.09 354.51 380.99 392.78 396.81 421.28
445.89 449.86 450.91 452.18 461.89 468.32 474.80 488.36
491.25 502.91 511.32 515.53 529.36 531.01 556.30 569.55
575.78 593.55 595.66 611.15 619.71 623.35 645.46 648.71
665.07 680.68 685.35 696.30 703.77 708.36 717.41 731.94
732.34 750.79 755.79 759.75 771.22 775.15 777.89 779.50
795.80 802.07 817.32 843.35 843.54 877.12 883.25 890.05
898.38 899.87 903.13 904.88 914.13 923.76 926.79 937.88
951.75 956.49 960.20 962.48 967.11 969.77 970.12 978.47
988.08 1004.23 1010.22 1023.09 1026.27 1030.72 1031.24
1043.18 1055.53 1066.66 1075.23 1081.77 1089.15 1109.98
1111.67 1119.17 1129.45 1138.68 1140.95 1144.74 1153.60
1165.71 1176.73 1185.61 1190.73 1196.83 1198.32 1198.94
1221.10 1239.30 1249.87 1254.67 1257.50 1266.02 1267.30
1273.92 1288.74 1293.30 1305.47 1315.55 1320.21 1324.25
1334.89 1338.63 1342.62 1345.32 1354.68 1355.25 1358.16
1360.71 1374.93 1378.67 1388.87 1394.34 1397.18 1409.60
1422.27 1428.12 1433.28 1436.25 1443.00 1444.07 1446.29
1463.27 1477.85 1494.13 1501.36 1504.12 1510.17 1513.76
1514.99 1516.27 1517.89 1520.16 1521.35 1523.55 1527.12
1531.99 1534.71 1541.24 1542.90 1548.70 1562.80 1568.78
1578.52 1592.27 1632.51 1646.98 1665.26 1676.21 1695.25
1712.16 1732.03 1748.95 1767.63 1777.99 3041.84 3054.75
3057.30 3061.76 3067.56 3069.19 3071.75 3075.66 3081.89
3101.66 3110.30 3122.61 3125.25 3136.86 3140.20 3142.31
3144.06 3144.76 3151.06 3161.73 3166.94 3177.32 3178.41
3207.50 3216.21 3217.18 3217.80 3226.31 3231.51 3236.31
3244.24 3249.88 3253.49 3260.79 3285.25 3286.59 3288.47

==============================
syn-6 (conformer10/12)
==============================
20.08 22.20 28.97 34.50 42.28 47.84 55.77 63.56 75.20
78.19 84.94 86.41 88.25 102.02 115.19 117.22 120.49
131.26 144.80 150.15 162.36 181.86 189.77 203.40 217.69
224.86 239.74 241.37 244.15 247.95 253.90 260.32 261.93
271.34 272.63 278.77 300.58 304.42 313.58 318.56 322.26
334.75 344.79 346.98 354.52 380.97 392.76 396.79 421.37
445.94 449.86 450.92 452.17 461.89 468.30 474.59 488.35
490.95 502.93 511.31 515.40 529.31 531.00 556.26 569.55
575.70 593.58 595.68 611.15 619.71 623.31 645.43 648.67
665.09 680.65 685.38 696.32 703.75 708.36 717.38 731.94
732.35 750.74 755.81 759.69 771.18 775.15 777.86 779.53
795.89 802.08 817.32 843.35 843.49 876.84 883.33 890.08
898.37 900.02 902.99 904.89 914.17 923.74 926.78 937.86
951.71 956.47 960.22 962.47 967.14 969.78 970.12 978.45
988.17 1004.29 1010.23 1023.17 1026.26 1030.72 1031.29
1043.17 1055.53 1066.66 1075.20 1081.80 1089.11 1109.96
1111.65 1119.17 1129.42 1138.65 1140.97 1144.72 1153.63
1165.73 1176.73 1185.60 1190.65 1196.82 1198.35 1198.97
1221.12 1239.28 1249.82 1254.72 1257.52 1266.04 1267.30
1273.92 1288.66 1293.23 1305.43 1315.53 1320.20 1324.23

1334.86 1338.62 1342.53 1345.28 1354.70 1355.26 1358.12
1360.74 1374.83 1378.64 1388.87 1394.32 1397.17 1409.63
1422.52 1428.20 1433.31 1436.21 1442.92 1444.07 1446.29
1463.29 1477.88 1494.17 1501.37 1504.13 1510.17 1513.76
1514.98 1516.27 1517.89 1520.15 1521.35 1523.54 1527.12
1532.01 1534.79 1541.27 1542.94 1548.73 1562.79 1568.70
1578.52 1592.33 1632.45 1647.00 1665.29 1676.25 1695.26
1712.11 1732.30 1748.97 1767.64 1778.00 3041.81 3054.72
3057.26 3061.72 3067.52 3069.16 3071.75 3075.63 3081.87
3101.62 3110.22 3122.54 3125.18 3136.84 3140.20 3142.30
3144.05 3144.75 3151.04 3161.67 3166.93 3177.32 3178.40
3207.44 3216.17 3217.27 3217.75 3226.18 3231.48 3236.22
3244.21 3249.84 3253.51 3260.71 3285.25 3286.62 3288.33

==============================
syn-6 (conformer11/12)
==============================
21.63 24.59 29.58 38.39 43.07 47.35 58.65 65.80 71.65
80.42 83.88 88.86 91.63 99.13 115.22 118.01 122.57
132.05 143.79 149.76 159.54 182.38 185.51 191.01 216.24
222.69 229.45 242.76 246.86 249.69 251.10 261.72 263.09
272.30 273.72 278.46 298.21 304.15 312.23 318.56 319.70
334.54 347.53 348.85 355.68 379.64 391.64 396.14 423.37
443.53 448.24 449.10 451.45 462.36 468.48 476.05 487.06
492.37 502.42 510.27 514.88 529.11 530.91 556.48 570.03
576.33 591.17 596.27 613.49 620.12 624.95 646.78 649.54
664.36 683.32 685.35 696.04 703.56 712.54 718.19 732.35
735.39 752.42 755.67 762.33 771.53 775.83 777.64 780.27
794.39 801.82 819.27 842.97 843.39 873.26 883.23 888.23
895.24 897.27 900.70 902.09 914.91 924.21 928.29 939.94
951.68 953.49 959.42 962.39 968.53 970.61 973.54 973.88
986.47 1002.57 1010.04 1020.66 1023.37 1028.45 1031.92
1044.95 1054.84 1066.54 1076.18 1084.85 1087.20 1110.58
1112.18 1117.83 1129.54 1135.25 1140.73 1145.85 1153.58
1165.14 1175.56 1185.75 1190.59 1196.17 1198.31 1199.59
1218.98 1240.01 1250.67 1253.87 1257.82 1267.03 1268.05
1274.15 1287.94 1295.08 1306.24 1316.44 1318.37 1325.23
1333.22 1339.78 1343.55 1346.19 1354.01 1354.72 1356.62
1358.98 1376.97 1379.56 1387.43 1394.36 1396.50 1407.35
1421.29 1428.68 1435.20 1438.71 1442.74 1443.38 1446.63
1463.90 1477.84 1492.52 1498.02 1501.57 1510.19 1511.81
1514.83 1515.70 1518.53 1519.49 1521.00 1524.83 1527.49
1532.53 1538.43 1540.95 1543.07 1550.75 1560.59 1569.37
1577.79 1590.91 1632.25 1644.18 1666.50 1673.99 1695.31
1711.62 1733.89 1747.58 1766.73 1777.69 3043.82 3054.20
3058.66 3062.19 3068.60 3069.78 3072.48 3076.53 3082.73
3100.37 3103.97 3122.67 3125.60 3136.56 3139.47 3139.77
3144.13 3144.72 3151.64 3163.84 3167.58 3178.15 3178.83
3202.36 3207.01 3215.69 3217.98 3226.12 3231.70 3235.99
3242.99 3249.17 3255.28 3260.50 3285.73 3287.92 3290.41

==============================
syn-6 (conformer12/12)
==============================
21.55 24.72 29.85 38.45 43.01 47.41 58.74 66.13 71.87
80.48 84.07 88.62 91.79 99.32 115.27 117.97 122.73
131.93 143.91 149.37 159.69 182.48 185.54 191.13 216.35
222.90 229.69 242.78 247.28 250.02 251.66 261.76 263.08
272.11 273.94 278.44 298.45 304.31 312.25 318.64 319.75
334.58 347.38 348.55 355.61 379.68 391.70 396.05 423.31
443.52 448.02 449.22 451.46 462.35 468.46 475.94 487.03
492.21 502.42 510.25 515.09 529.19 530.90 556.60 569.98
576.10 591.05 596.34 613.53 620.14 624.88 646.78 649.55
664.39 683.34 685.33 696.04 703.76 712.59 718.18 732.28
735.21 752.43 755.59 762.30 771.57 775.99 777.69 780.44
794.49 801.82 819.12 842.97 843.20 873.14 883.14 887.92
894.72 897.39 900.74 902.00 914.88 924.20 928.32 940.09
951.81 953.63 959.44 962.42 968.61 970.64 973.58 974.16
986.50 1002.69 1010.20 1020.56 1023.02 1028.38 1032.06
1044.96 1054.93 1066.56 1076.24 1084.91 1087.35 1110.57
1112.10 1117.68 1129.48 1135.26 1140.80 1145.91 1153.68
1165.17 1175.46 1185.75 1190.51 1196.16 1198.34 1199.57
1218.79 1239.93 1250.68 1253.98 1257.91 1266.98 1268.00
1274.11 1287.84 1295.13 1306.14 1316.38 1318.39 1325.20
1333.15 1339.79 1343.54 1346.20 1353.94 1354.65 1356.58
1359.07 1376.97 1379.59 1387.44 1394.30 1396.61 1407.24
1421.69 1429.05 1435.27 1438.81 1442.77 1443.39 1446.74
1464.01 1477.94 1492.26 1497.85 1501.48 1510.17 1511.90
1514.80 1515.77 1518.51 1519.53 1520.94 1524.98 1527.49
1532.37 1538.39 1540.89 1543.13 1550.76 1560.47 1569.38
1577.79 1590.80 1632.16 1644.34 1666.50 1674.08 1695.32
1711.67 1733.94 1747.30 1766.82 1777.91 3043.73 3054.22
3058.58 3062.20 3068.65 3069.82 3072.57 3076.54 3082.65
3100.45 3103.83 3122.65 3125.60 3136.43 3139.55 3139.84
3144.19 3144.88 3151.62 3163.91 3167.68 3178.26 3178.95
3202.35 3207.02 3215.80 3218.01 3226.31 3231.66 3236.09
3242.97 3249.17 3255.30 3260.59 3285.58 3288.01 3291.51

==============================
anti-6 (conformer2/5)
==============================
19.04 20.65 24.43 29.26 41.28 43.27 55.19 60.88 64.80
73.52 80.14 84.81 90.89 105.51 110.79 112.34 125.64
129.83 144.70 148.20 162.45 177.97 180.83 192.63 215.29
230.70 236.33 239.82 242.59 247.63 250.52 259.78 260.92
266.94 274.14 275.40 298.07 302.79 310.90 318.10 318.45
338.61 340.78 350.86 359.92 376.38 393.92 396.25 417.64
444.19 445.05 448.25 449.51 463.38 465.48 471.23 486.46
488.21 500.47 503.55 516.12 529.34 531.83 552.17 562.60
569.24 584.90 594.14 596.82 620.27 621.45 639.62 647.52
652.10 680.22 681.66 694.24 702.38 705.23 715.58 723.41
732.18 754.17 757.93 760.63 771.86 774.65 776.03 777.12
786.02 800.37 811.84 838.62 843.27 872.10 878.52 886.90
891.57 900.03 901.83 912.66 921.95 923.98 929.55 937.25
943.83 955.04 956.27 959.25 968.15 969.40 973.84 977.11
989.27 1009.53 1015.17 1021.15 1024.74 1026.46 1030.62
1041.46 1052.97 1065.28 1078.66 1089.72 1092.60 1107.37
1109.28 1113.62 1133.98 1137.02 1138.57 1143.50 1155.57
1169.57 1176.00 1185.74 1186.80 1194.66 1196.48 1198.77
1220.27 1238.11 1252.29 1252.59 1260.47 1262.53 1271.15
1271.20 1285.91 1288.24 1304.14 1313.05 1322.42 1325.09
1330.04 1334.46 1339.70 1349.37 1352.87 1353.87 1353.97
1356.35 1362.05 1379.57 1388.99 1395.27 1408.70 1409.81
1430.85 1435.39 1440.00 1442.09 1444.78 1448.48 1463.03
1476.86 1478.67 1487.94 1495.33 1498.26 1501.01 1513.41
1513.75 1514.37 1515.28 1517.39 1520.71 1522.54 1529.44

1530.25 1533.53 1543.17 1543.97 1547.05 1552.23 1567.10
1584.50 1599.72 1618.85 1670.61 1671.45 1698.97 1712.78
1720.32 1752.98 1757.79 1769.02 1785.18 3033.33 3054.92
3057.01 3062.66 3065.61 3067.80 3071.55 3073.80 3074.76
3080.16 3100.99 3114.54 3117.73 3132.85 3139.80 3143.67
3144.41 3145.16 3147.86 3162.75 3165.29 3178.30 3181.04
3203.43 3207.27 3215.88 3217.61 3229.20 3229.75 3240.36
3242.13 3242.63 3247.07 3264.24 3289.61 3297.49 3309.03

==============================
anti-6 (conformer3/5)
==============================
12.05 19.97 24.95 31.57 38.07 42.98 50.77 57.70 69.79
72.45 79.55 87.91 91.57 105.16 111.64 113.03 125.52
127.14 143.56 151.88 163.99 176.08 178.68 192.10 214.84
225.12 240.08 244.51 247.30 249.50 258.09 259.92 262.31
271.15 274.03 292.06 298.39 307.63 314.85 317.77 321.02
335.00 342.65 356.20 358.93 376.49 392.67 394.56 417.82
444.69 448.02 449.42 449.81 463.51 465.36 471.07 485.84
497.97 502.26 503.24 517.00 530.22 532.31 552.14 561.15
572.18 582.19 589.25 596.35 620.07 622.34 641.30 647.60
649.86 680.14 681.34 693.42 701.43 702.51 716.13 720.92
732.27 755.43 760.03 766.21 770.79 774.36 775.49 777.32
782.92 798.49 809.21 842.66 842.82 867.59 881.46 884.02
893.69 896.50 902.21 911.38 923.14 923.82 931.01 935.54
944.81 953.71 956.30 959.12 965.92 968.35 975.48 976.49
988.59 1008.75 1012.37 1018.69 1020.67 1027.29 1030.62
1041.54 1051.49 1066.12 1079.70 1090.18 1092.85 1100.16
1110.50 1116.15 1132.89 1135.43 1138.24 1142.63 1154.69
1170.50 1173.71 1185.61 1187.66 1194.02 1196.13 1200.28
1218.07 1237.09 1253.30 1253.64 1258.86 1265.92 1270.77
1271.63 1285.73 1289.85 1303.99 1314.34 1322.76 1324.21
1329.13 1339.36 1339.65 1350.39 1350.86 1353.72 1356.46
1357.82 1362.04 1379.53 1385.34 1389.86 1406.88 1410.17
1427.39 1433.95 1438.60 1438.79 1444.95 1446.84 1462.60
1477.59 1480.81 1489.88 1494.39 1496.99 1501.05 1513.39
1514.10 1516.43 1517.50 1518.29 1518.94 1524.24 1529.37
1530.30 1533.16 1542.92 1545.46 1546.99 1552.98 1568.78
1585.27 1599.89 1619.40 1671.15 1672.58 1699.33 1713.07
1721.99 1752.87 1759.09 1767.52 1785.13 3031.65 3053.96
3055.99 3061.87 3065.43 3067.51 3072.35 3074.39 3075.55
3080.86 3100.83 3108.35 3116.63 3136.02 3137.74 3143.84
3144.99 3146.23 3147.39 3165.29 3166.75 3178.15 3181.48
3199.02 3208.91 3215.75 3218.48 3229.66 3229.97 3240.67
3243.42 3243.90 3248.07 3263.33 3288.40 3296.16 3314.33

==============================
anti-6 (conformer4/5)
==============================
12.96 20.15 26.70 31.56 41.43 42.99 57.33 59.77 65.84
74.85 79.63 87.83 90.26 94.81 111.19 115.97 125.02
130.42 137.77 147.64 162.27 168.42 179.50 189.61 206.97
221.33 240.44 241.18 247.72 253.39 261.13 263.74 264.18
267.71 277.33 292.44 298.33 311.26 317.72 322.68 325.46
332.57 340.09 353.99 358.66 377.77 390.42 392.40 416.77
444.06 447.22 447.86 451.12 464.05 465.35 471.63 483.07
488.34 489.71 504.08 517.32 528.10 533.19 552.16 556.59
570.36 582.32 592.60 595.68 621.25 622.70 647.24 652.80
658.18 681.04 686.02 694.16 705.69 709.91 715.35 723.64
736.61 755.51 756.19 761.63 774.04 777.13 779.71 781.72
792.47 801.29 821.54 836.90 841.83 871.32 879.17 885.15
893.55 898.80 901.68 907.66 922.51 924.82 925.82 936.91
940.86 954.44 956.04 958.17 967.08 970.80 974.67 977.56
990.06 1004.83 1015.47 1020.92 1023.89 1025.35 1031.92
1043.55 1053.84 1067.99 1079.55 1087.60 1100.84 1109.21
1110.42 1114.66 1130.43 1136.10 1140.09 1146.89 1157.69
1170.72 1174.25 1185.99 1188.36 1195.59 1201.96 1202.31
1217.21 1240.37 1244.76 1248.22 1256.87 1264.05 1266.65
1269.47 1289.68 1290.02 1301.65 1313.74 1321.44 1323.42
1329.48 1337.37 1338.38 1346.90 1349.05 1352.08 1352.65
1353.42 1356.80 1382.25 1386.52 1394.31 1406.66 1408.71
1431.94 1438.15 1439.51 1441.90 1442.47 1447.36 1462.42
1479.22 1488.96 1496.41 1499.73 1503.36 1509.63 1513.53
1514.89 1515.35 1517.75 1520.03 1521.30 1523.67 1529.60
1533.47 1537.62 1539.35 1541.42 1542.93 1554.98 1568.51
1582.77 1594.92 1602.57 1672.32 1675.69 1703.33 1712.76
1721.01 1753.58 1759.27 1768.74 1784.63 3024.52 3056.68
3057.72 3062.92 3066.24 3070.25 3072.16 3073.88 3075.97
3080.61 3099.84 3104.02 3116.37 3134.14 3137.89 3142.98
3144.50 3145.49 3150.15 3168.89 3174.54 3178.87 3180.14
3210.29 3214.73 3223.66 3226.94 3229.48 3231.74 3235.80
3245.18 3245.52 3249.84 3264.06 3280.69 3299.82 3337.99

==============================
anti-6 (conformer5/5)
==============================
15.61 17.00 27.54 34.52 45.11 46.81 58.50 63.59 76.93
81.07 84.70 89.46 95.80 103.51 112.90 121.88 135.04
138.93 143.26 153.16 164.46 169.53 172.58 202.98 215.14
228.25 238.53 240.24 248.48 257.74 258.98 261.77 265.02
271.59 278.68 287.05 305.33 320.37 321.48 326.71 334.60
343.35 354.70 358.97 381.05 385.50 395.00 418.34 421.63
433.93 448.31 448.67 455.62 461.03 463.99 465.11 481.44
495.17 501.18 514.23 530.40 534.46 545.31 548.28 576.47
587.43 592.63 602.07 621.81 627.17 645.81 648.37 679.26
681.49 690.78 706.39 711.21 716.36 733.28 739.63 759.61
761.25 764.32 767.11 773.67 777.44 782.02 784.22 798.35
801.30 828.47 838.41 840.14 842.38 845.01 861.39 878.55
884.57 898.41 898.91 904.60 907.07 921.23 925.40 931.51
951.01 953.53 955.38 963.03 967.30 971.21 975.31 976.35
993.07 1004.76 1013.46 1022.08 1022.16 1026.44 1027.83
1039.00 1048.04 1053.48 1070.86 1079.79 1089.19 1096.20
1109.43 1110.97 1120.03 1137.53 1142.38 1145.59 1164.45
1170.69 1178.73 1190.32 1196.45 1199.44 1202.41 1211.03
1215.32 1234.34 1243.45 1248.42 1253.80 1263.92 1279.56
1286.55 1289.49 1290.39 1294.69 1313.70 1316.30 1323.77
1329.97 1337.44 1347.28 1348.84 1349.62 1351.56 1353.32
1358.15 1373.94 1383.83 1387.04 1400.97 1406.79 1416.07
1428.10 1432.54 1437.45 1442.44 1444.60 1463.33 1473.36
1476.85 1478.66 1491.29 1497.12 1504.12 1512.53 1514.59
1515.20 1516.40 1517.88 1520.58 1523.58 1527.60 1530.83
1534.60 1538.98 1541.85 1542.58 1545.24 1562.33 1569.46
1582.08 1595.31 1671.85 1678.15 1705.62 1709.02 1712.19
1719.30 1753.32 1769.19 1784.12 1792.06 3022.60 3055.38
3059.86 3061.59 3063.27 3067.72 3070.84 3071.39 3073.61
3080.05 3101.15 3108.21 3113.93 3132.27 3134.95 3136.60

3143.24 3144.88 3146.86 3166.27 3167.21 3171.57 3179.79
3180.04 3208.59 3216.69 3222.03 3230.99 3232.22 3234.44
3242.86 3245.61 3248.94 3265.00 3273.52 3273.99 3362.51

==============================
syn-4
==============================
24.74 25.90 29.95 31.54 40.32 53.77 54.48 64.11 74.37
78.65 83.85 89.25 94.61 113.56 118.13 121.73 128.78
132.60 149.70 155.50 163.19 187.60 200.98 213.28 226.91
232.22 247.17 250.03 253.91 256.60 257.73 260.96 267.53
276.58 287.67 298.53 305.18 320.53 322.88 331.06 338.93
340.68 347.07 378.68 384.18 394.83 413.90 417.15 430.89
441.59 443.94 452.58 458.79 462.44 471.51 474.03 484.97
485.80 504.45 516.18 524.61 531.45 545.58 567.85 574.67
588.97 595.93 616.73 619.59 638.88 644.93 651.53 671.66
678.08 703.43 708.27 714.06 729.71 743.99 745.80 749.58
751.37 753.98 759.42 765.56 768.53 777.15 799.22 807.82
827.72 839.12 840.22 849.90 859.90 868.37 879.30 882.10
891.22 894.29 899.96 906.57 911.54 923.75 933.83 935.97
940.68 941.45 942.58 945.42 953.71 958.94 965.08 969.21
977.36 982.79 999.65 999.94 1012.33 1020.21 1020.91
1038.41 1038.63 1049.68 1056.55 1064.16 1069.38 1082.48
1099.00 1108.22 1119.79 1124.20 1130.66 1150.77 1154.74
1158.77 1165.00 1185.16 1187.85 1187.96 1196.52 1197.76
1234.18 1235.50 1236.41 1241.54 1253.77 1257.94 1261.44
1270.55 1274.14 1277.37 1292.45 1299.05 1309.55 1312.66
1322.44 1323.57 1332.37 1338.48 1339.10 1342.51 1344.20
1350.94 1363.72 1383.85 1384.52 1390.87 1395.29 1411.14
1414.98 1420.41 1422.73 1426.47 1432.36 1441.66 1466.79
1472.02 1485.30 1488.57 1493.48 1495.61 1501.30 1506.71
1510.46 1511.51 1512.95 1514.06 1515.36 1517.59 1519.12
1522.15 1525.21 1530.28 1536.56 1544.08 1560.01 1573.46
1616.91 1631.96 1632.83 1656.34 1665.95 1668.67 1673.08
1687.41 1727.45 1732.29 1738.38 1752.80 3017.14 3021.40
3023.90 3033.59 3039.58 3042.65 3044.14 3044.94 3049.42
3065.66 3071.15 3092.39 3102.77 3103.90 3110.55 3113.58
3115.02 3118.47 3123.58 3129.72 3135.91 3146.81 3149.72
3167.39 3177.80 3188.55 3189.05 3200.42 3203.66 3210.29
3212.91 3217.88 3226.14 3234.45 3236.93 3247.04 3280.77

==============================
anti-4
==============================
17.73 24.20 31.17 40.50 42.89 54.64 63.45 68.95 74.81
77.58 85.17 97.22 108.23 121.57 127.35 128.50 134.39
140.34 155.57 161.20 173.72 183.55 196.75 209.96 222.14
235.27 243.57 251.10 256.26 256.81 258.69 265.18 273.70
277.33 281.68 287.99 312.94 324.11 332.77 339.24 339.90
341.34 344.13 379.34 386.62 393.72 414.90 416.44 431.93
442.69 446.56 448.76 456.80 459.86 461.92 471.51 479.84
489.00 505.88 518.17 526.04 532.94 543.46 563.95 569.37
589.63 593.90 616.84 622.30 638.86 644.80 651.69 671.02
675.42 703.93 708.21 713.44 731.11 740.27 744.07 747.31
748.87 753.53 761.45 763.66 770.86 776.34 798.46 806.67
828.51 838.16 840.20 843.44 861.23 874.67 880.80 885.92
889.07 889.43 900.37 911.21 912.57 924.19 926.09 928.90
937.05 940.21 942.28 944.65 946.92 951.19 955.92 962.27
979.77 981.43 995.62 1003.48 1010.10 1018.66 1024.08
1038.77 1043.47 1052.39 1059.30 1064.72 1069.51 1087.96
1092.22 1110.43 1118.64 1127.11 1133.53 1151.98 1156.37
1157.25 1160.52 1185.82 1187.76 1190.10 1193.12 1197.01
1232.80 1234.42 1237.83 1242.92 1255.72 1259.10 1262.21
1267.11 1275.69 1276.29 1297.63 1299.05 1306.27 1311.64
1317.30 1330.27 1333.50 1334.32 1339.14 1341.11 1343.19
1344.47 1365.06 1381.70 1382.53 1392.16 1398.21 1410.22
1413.57 1421.97 1423.12 1431.61 1436.64 1448.07 1462.86
1465.26 1483.67 1493.52 1494.11 1497.94 1501.24 1505.01
1506.96 1508.36 1512.12 1515.14 1517.28 1521.15 1522.56
1523.01 1526.03 1530.31 1535.14 1543.30 1562.21 1573.64
1616.41 1630.57 1633.10 1655.58 1664.41 1669.45 1675.43
1686.76 1730.34 1730.83 1741.99 1751.10 3005.85 3018.79
3024.00 3035.05 3036.77 3040.18 3043.43 3044.78 3051.26
3066.65 3081.21 3086.26 3096.13 3111.12 3114.54 3116.71
3117.63 3119.44 3123.92 3134.35 3137.98 3150.40 3151.88
3179.17 3188.55 3193.40 3201.75 3205.16 3206.60 3211.06
3216.46 3217.92 3222.26 3235.67 3235.98 3246.77 3297.07

==============================
syn-7
==============================
19.65 26.37 31.19 38.93 43.58 46.43 55.87 67.55 69.46
72.70 80.74 90.86 97.19 105.13 115.45 117.71 126.59
129.03 132.03 143.73 160.61 172.62 185.84 202.71 203.95
212.70 216.20 227.00 245.66 250.72 253.51 254.87 259.95
267.91 271.30 278.20 290.98 293.51 304.15 311.26 320.95
325.61 327.12 347.60 364.98 376.58 386.25 404.81 410.67
414.21 428.97 433.17 441.53 445.61 450.28 461.97 470.57
480.34 495.08 496.15 503.37 526.42 526.85 543.30 562.59
576.79 583.94 586.29 607.67 614.35 641.23 643.79 654.86
658.57 661.96 677.01 677.72 694.52 700.71 709.49 711.33
728.16 737.85 742.31 753.03 759.80 763.77 770.09 792.79
793.18 809.82 813.71 817.57 830.68 836.06 838.37 845.75
868.13 879.45 880.01 886.91 897.85 901.45 905.10 907.55
914.73 922.56 927.33 933.18 943.13 949.91 955.09 959.29
961.24 968.66 994.84 1000.20 1002.64 1012.25 1021.30
1022.60 1033.18 1035.35 1043.30 1046.65 1067.98 1083.15
1093.55 1100.70 1112.41 1114.74 1123.38 1125.66 1139.58
1152.44 1160.60 1168.13 1176.73 1181.01 1184.76 1192.45
1193.37 1213.59 1225.49 1230.15 1236.15 1247.79 1252.42
1261.43 1263.74 1268.84 1288.49 1292.74 1305.57 1309.42
1315.11 1320.50 1325.73 1330.97 1334.36 1336.22 1340.73
1347.54 1368.18 1373.04 1375.94 1381.07 1384.91 1390.51
1412.31 1413.75 1424.91 1426.94 1430.87 1433.72 1460.22
1469.62 1476.67 1478.65 1482.33 1488.38 1493.98 1496.44
1502.37 1505.29 1506.15 1509.36 1510.86 1511.10 1513.82
1516.21 1520.01 1522.25 1526.01 1527.28 1536.54 1549.41
1560.63 1575.88 1594.83 1606.40 1622.52 1639.96 1652.37
1661.35 1686.32 1694.06 1727.03 1729.21 3022.34 3024.26
3032.10 3032.58 3037.87 3039.48 3044.58 3046.88 3048.05
3066.96 3086.81 3088.77 3094.28 3106.29 3109.37 3110.17
3110.93 3114.70 3120.34 3136.94 3141.66 3147.11 3151.36
3157.78 3184.55 3185.50 3195.57 3199.27 3204.40 3211.82
3212.12 3217.45 3234.86 3240.42 3245.73 3247.09 3311.29

==============================

syn-7-TS

==============================

-346.95 20.10 23.48 30.22 40.39 48.87 55.29 59.28 62.4
77.26 89.07 93.00 95.30 106.96 112.70 126.85 131.03
137.28 148.11 157.25 159.96 182.11 197.77 216.74 218.81
224.92 249.29 253.66 255.19 256.80 258.28 271.30 274.05
277.39 289.53 299.11 302.56 305.99 320.45 327.31 332.69
341.32 364.84 367.79 377.81 388.76 394.85 412.35 424.68
430.15 434.49 442.41 446.01 454.44 459.68 466.12 470.80
485.20 498.98 512.96 526.73 541.05 545.92 562.39 570.34
581.80 588.99 592.09 599.92 608.88 624.83 643.38 650.67
673.10 676.92 691.21 697.14 711.12 712.62 714.04 731.29
736.75 744.46 748.63 757.96 759.31 768.82 775.69 787.17
789.14 811.97 814.95 824.98 834.91 846.82 851.61 862.59
866.55 873.10 882.92 888.20 891.58 896.12 905.82 910.57
924.10 929.12 932.26 934.28 936.44 942.05 944.21 961.12
961.76 964.81 976.16 976.91 998.75 1000.47 1002.52
1010.15 1022.43 1023.54 1035.09 1054.64 1057.75 1066.17
1096.31 1104.06 1119.02 1125.11 1127.58 1138.00 1143.32
1154.46 1160.08 1173.92 1182.24 1182.75 1186.91 1187.85
1194.89 1201.01 1220.23 1228.56 1232.71 1237.75 1255.40
1255.75 1259.03 1269.08 1271.36 1272.96 1276.95 1289.05
1300.47 1303.14 1313.52 1320.47 1334.76 1335.54 1336.81
1342.41 1358.91 1367.78 1375.39 1382.17 1383.41 1389.76
1400.00 1409.40 1413.90 1425.10 1426.82 1434.17 1436.26
1448.49 1475.13 1477.12 1486.24 1488.61 1491.95 1494.32
1497.91 1505.64 1508.03 1510.36 1511.68 1513.84 1516.22
1517.06 1518.86 1519.25 1522.30 1524.46 1528.64 1545.56
1562.18 1571.52 1604.19 1615.86 1622.47 1634.11 1656.82
1667.21 1687.82 1709.85 1738.15 1748.77 3016.14 3021.68
3031.28 3036.87 3037.28 3038.74 3044.01 3047.00 3064.63
3085.94 3092.14 3094.73 3098.04 3106.47 3110.47 3112.12
3114.55 3121.06 3127.40 3148.47 3151.15 3162.04 3165.02
3178.70 3186.30 3188.61 3190.63 3199.47 3202.09 3208.67
3217.90 3222.71 3231.53 3239.45 3248.47 3260.67 3292.79

==============================

syn-7-TS'

==============================

-488.49 18.35 26.05 33.00 40.50 46.66 53.86 65.76 70.18
78.71 85.15 93.96 101.67 105.36 113.13 115.64 128.85
131.81 146.01 155.59 164.53 176.18 193.06 203.51 211.52
219.03 245.85 248.43 251.31 254.99 259.73 268.16 278.56
286.32 288.33 300.20 312.79 314.26 318.48 320.79 323.79
327.15 349.33 359.44 370.48 377.51 386.90 395.10 405.58
419.50 430.01 442.59 443.92 449.69 460.91 470.34 472.37
480.98 498.17 500.06 507.88 524.65 543.83 549.69 566.33
580.81 585.65 588.34 614.07 623.23 644.45 646.32 653.10
669.25 676.32 688.89 693.86 702.69 713.07 718.98 737.08
741.52 745.57 752.29 755.74 765.51 774.87 796.37 800.82
804.85 814.21 820.73 824.75 830.64 841.70 850.18 867.48
870.63 883.80 885.89 890.20 891.25 904.59 912.51 916.67
919.41 922.99 929.18 935.63 939.91 943.62 947.72 958.90
967.62 969.98 976.13 988.32 1000.46 1002.31 1003.29
1013.06 1022.65 1034.69 1035.49 1056.05 1056.12 1065.92
1092.10 1099.21 1121.64 1125.64 1128.29 1136.05 1142.65
1143.87 1155.29 1162.28 1176.65 1186.97 1187.49 1191.47
1203.35 1222.41 1228.83 1232.78 1237.19 1251.97 1252.76
1262.22 1263.27 1268.55 1271.56 1278.08 1288.85 1298.84
1303.69 1304.92 1320.17 1332.13 1333.50 1335.28 1336.63
1341.17 1355.10 1368.58 1378.85 1382.47 1384.46 1390.10
1394.77 1412.89 1414.33 1424.85 1427.33 1434.12 1437.79
1462.52 1478.71 1479.55 1487.50 1490.31 1491.43 1494.11
1505.03 1505.85 1508.45 1509.86 1511.16 1513.91 1516.77
1517.20 1519.67 1520.30 1523.31 1529.48 1533.09 1553.69
1571.73 1574.63 1609.90 1613.30 1629.06 1631.33 1648.34
1661.83 1677.00 1698.03 1710.62 1734.03 3019.72 3022.52
3032.03 3037.34 3038.92 3044.13 3045.01 3047.91 3065.99
3081.35 3086.31 3093.51 3096.75 3106.72 3109.92 3110.34
3111.82 3120.09 3137.97 3138.07 3139.27 3147.28 3155.27
3177.73 3186.22 3188.04 3198.22 3201.95 3206.43 3213.64
3214.07 3214.36 3221.30 3235.01 3239.59 3248.43 3260.69

==============================

syn-8

==============================

15.22 23.58 31.70 36.49 38.95 46.18
56.24 57.48 72.78 79.15 88.00 96.76
105.61 113.25 123.24 131.71 142.14 152.33
159.54 165.53 173.83 190.67 210.17 211.99
219.43 219.63 241.20 248.97 253.33 256.53
257.39 268.22 278.15 286.70 289.95 295.93
301.12 303.17 307.98 321.19 324.22 331.66
354.89 358.23 375.68 378.26 390.11 406.82
412.21 429.73 438.37 444.06 451.78 459.15
462.75 470.09 482.45 498.26 507.60 525.68
542.61 544.86 550.18 558.54 562.62 572.64
586.47 590.32 613.65 615.24 634.06 640.51
651.15 667.41 672.92 682.22 695.82 712.00
713.83 716.53 721.62 738.81 744.66 746.77
754.49 764.55 769.32 775.33 795.79 797.92
810.38 823.81 825.93 848.43 854.95 861.69
873.99 878.44 883.57 889.40 891.11 906.39
911.72 923.03 927.26 928.88 935.05 936.08
937.17 942.18 943.32 958.83 967.83 977.26
978.57 984.90 998.95 1000.43 1003.29 1006.93
1010.96 1024.00 1034.64 1046.85 1054.41 1064.43
1083.88 1095.83 1102.22 1116.06 1124.97 1126.59
1129.67 1140.73 1141.97 1151.37 1152.91 1159.69
1180.59 1184.48 1186.31 1188.83 1195.62 1219.68
1227.05 1229.03 1234.40 1237.90 1241.50 1250.82
1259.84 1269.71 1270.97 1287.90 1297.70 1301.96
1305.44 1311.15 1320.10 1322.66 1331.51 1336.85
1342.98 1368.22 1368.81 1379.90 1381.98 1382.56
1389.98 1394.47 1396.39 1409.48 1414.26 1424.82
1427.17 1433.20 1434.58 1473.72 1479.92 1483.61
1489.01 1492.12 1493.71 1494.45 1499.10 1505.39
1507.69 1508.98 1510.10 1514.17 1515.15 1516.55
1517.72 1519.28 1522.49 1529.98 1535.63 1561.48
1574.25 1611.76 1614.43 1631.97 1638.44 1652.83
1665.29 1686.93 1720.29 1740.26 1750.62 3020.19
3023.11 3030.26 3032.08 3037.95 3039.58 3044.84
3047.89 3051.29 3065.01 3065.97 3088.96 3097.71
3107.48 3111.84 3112.95 3114.64 3116.21 3120.60
3122.34 3124.93 3128.02 3148.82 3158.04 3180.87
3186.06 3192.43 3194.91 3201.06 3201.87 3209.11

3215.38 3221.44 3238.35 3249.05 3263.09 3305.44

==============================
syn-8'
==============================
1.05 21.58 32.34 38.93 44.95 56.65
64.63 72.55 76.12 84.27 88.32 101.32
104.54 107.21 117.76 123.44 132.47 138.45
150.35 159.77 185.62 195.70 200.34 215.69
224.60 241.08 247.76 252.78 255.44 259.70
269.71 281.57 284.28 287.89 301.73 307.45
309.62 319.83 327.34 333.66 337.95 347.76
372.66 373.39 376.54 391.03 408.13 414.10
429.17 433.99 434.89 445.58 457.78 459.45
462.61 471.02 474.15 484.92 504.36 507.42
524.79 544.60 550.90 566.70 568.68 586.31
588.84 594.57 628.07 630.68 633.79 641.80
654.70 670.15 675.57 686.59 712.10 714.61
715.63 743.56 746.52 748.94 759.44 766.38
770.87 776.72 782.38 803.08 807.97 812.29
826.84 834.61 843.79 845.86 871.57 878.14
882.64 884.60 887.55 896.15 906.00 915.76
922.57 923.14 924.50 929.13 940.10 941.94
946.38 954.92 961.00 964.27 982.18 983.47
989.72 1002.22 1002.86 1011.71 1023.40 1035.77
1039.91 1047.79 1061.69 1067.03 1070.17 1095.03
1101.71 1115.73 1125.64 1127.40 1141.60 1144.97
1155.69 1161.94 1167.43 1172.75 1179.30 1186.74
1194.56 1197.47 1215.76 1223.23 1226.98 1227.76
1236.89 1247.38 1253.33 1259.12 1259.34 1267.29
1270.87 1285.83 1298.07 1300.97 1304.33 1316.38
1319.97 1320.88 1336.84 1337.63 1338.99 1341.20
1345.42 1358.62 1368.67 1380.19 1382.41 1382.93
1389.91 1393.38 1405.28 1413.67 1414.23 1425.52
1425.84 1434.27 1474.65 1475.99 1476.23 1483.99
1488.50 1489.85 1494.44 1498.98 1500.65 1505.92
1508.64 1511.05 1511.27 1513.59 1514.27 1516.70
1519.60 1523.64 1527.10 1529.66 1546.50 1563.12
1580.83 1614.39 1630.17 1645.52 1654.03 1664.07
1664.38 1687.82 1710.07 1727.35 1738.19 2999.26
3002.40 3017.85 3021.53 3024.24 3030.91 3036.60
3038.51 3043.79 3046.56 3064.67 3069.76 3083.55
3088.65 3094.31 3094.48 3106.12 3109.36 3110.51
3116.34 3120.95 3130.18 3130.57 3147.56 3169.50
3180.01 3186.63 3187.99 3196.90 3201.93 3205.14
3214.61 3215.27 3223.31 3242.80 3252.43 3264.85

==============================
os(syn-8)
==============================
13.85 24.60 35.86 39.50 45.35 51.71
57.51 59.26 77.38 78.36 90.39 94.05
103.13 112.52 120.96 129.60 134.27 141.69
162.14 172.68 181.54 190.65 209.73 212.25
219.52 232.66 244.50 249.19 253.59 256.05
257.84 268.51 277.37 281.80 290.43 295.11
296.61 301.93 308.07 312.54 324.43 331.34
353.87 357.89 375.61 378.29 390.21 403.13
411.80 430.33 438.44 444.29 451.43 459.80
468.36 470.46 482.90 498.30 507.56 525.65
533.59 543.82 551.76 557.35 562.60 572.09
586.72 590.62 613.65 614.83 632.71 640.54
652.33 670.98 673.82 682.32 693.66 708.42
714.40 716.16 719.65 736.61 744.68 747.57
754.82 764.46 766.04 776.26 795.98 799.40
811.55 824.76 826.79 848.75 861.12 862.50
874.17 880.05 884.44 889.39 890.98 906.35
911.15 923.09 927.45 932.18 935.42 936.58
938.11 942.36 944.00 958.78 968.31 977.71
979.46 988.20 996.93 1000.81 1003.51 1003.88
1011.28 1024.00 1034.69 1046.81 1054.29 1063.96
1079.49 1095.67 1101.98 1106.07 1116.23 1125.04
1126.66 1131.32 1142.18 1151.20 1152.67 1159.97
1180.53 1184.44 1186.74 1189.22 1195.45 1216.03
1227.08 1228.89 1234.20 1237.57 1241.78 1250.86
1259.70 1269.69 1270.89 1288.14 1298.20 1301.95
1305.03 1311.60 1320.33 1324.69 1331.47 1336.55
1343.33 1366.50 1368.22 1380.49 1382.88 1383.06
1390.23 1393.00 1394.31 1409.96 1414.27 1425.16
1427.43 1432.32 1434.45 1473.39 1479.78 1486.80
1488.79 1491.88 1493.79 1494.86 1499.31 1505.82
1507.99 1508.86 1510.28 1514.02 1514.91 1516.40
1517.52 1519.73 1522.64 1529.52 1535.79 1560.84
1574.21 1611.48 1614.80 1632.13 1638.60 1652.96
1664.89 1686.77 1719.46 1739.21 1750.69 3020.33
3022.94 3024.00 3031.73 3037.47 3039.28 3044.85
3047.89 3048.56 3065.83 3070.42 3089.50 3098.36
3106.95 3110.78 3112.55 3112.71 3113.78 3118.46
3120.43 3122.02 3128.48 3128.56 3148.84 3180.18
3185.86 3192.11 3197.61 3200.92 3201.75 3208.94
3215.33 3220.99 3232.93 3238.30 3249.56 3301.89

==============================
os(syn-8')
==============================
19.66 23.10 33.14 40.67 47.83 56.82
71.45 76.52 86.64 89.67 100.74 104.37
107.49 113.47 125.03 130.68 134.52 151.07
155.53 162.75 184.12 198.14 202.12 211.51
225.44 243.11 246.60 250.96 255.25 262.39
270.23 283.91 288.93 301.53 307.17 315.31
316.06 322.88 327.19 335.52 338.93 350.61
370.33 373.33 379.43 390.85 400.32 411.24
431.60 438.66 441.59 445.17 460.40 463.75
474.26 477.96 483.82 487.37 506.55 509.53
526.33 544.17 554.90 569.75 583.77 585.71
587.22 602.84 627.16 641.39 645.95 657.65
658.79 672.40 677.20 688.35 714.78 717.43
718.49 742.57 747.23 748.05 759.57 766.69
771.67 777.06 782.82 802.71 809.74 814.70
826.98 838.36 842.91 849.51 868.37 877.53
885.28 885.88 890.26 891.32 905.82 919.34
923.78 929.48 932.11 936.22 941.20 943.18
951.81 955.48 961.74 974.78 980.00 981.37
992.17 1001.78 1002.56 1013.20 1022.95 1036.24
1043.06 1054.40 1058.43 1069.13 1069.85 1092.72

1100.14 1122.04 1125.37 1129.33 1141.46 1146.93
1157.33 1158.60 1164.26 1174.09 1179.56 1187.91
1192.86 1196.73 1216.62 1224.86 1226.09 1229.11
1236.41 1241.42 1255.97 1258.75 1260.68 1267.70
1274.13 1286.90 1300.98 1301.62 1307.18 1316.44
1319.75 1323.51 1336.53 1339.73 1341.13 1342.01
1357.07 1368.38 1373.28 1382.44 1384.64 1385.50
1390.46 1393.39 1409.10 1413.73 1420.63 1424.67
1427.83 1434.24 1464.88 1476.22 1478.13 1479.70
1484.49 1488.24 1494.04 1502.24 1502.65 1506.14
1510.06 1511.49 1513.82 1516.03 1517.09 1519.02
1520.40 1524.17 1529.97 1533.62 1545.82 1562.36
1579.83 1616.18 1633.65 1645.88 1656.06 1664.95
1666.40 1686.71 1709.52 1719.62 1738.62 2951.88
2956.92 3019.91 3022.37 3031.83 3037.01 3038.79
3043.78 3046.88 3048.18 3060.06 3066.16 3068.42
3080.14 3085.87 3092.78 3105.79 3109.49 3109.96
3111.42 3120.30 3131.69 3138.51 3146.80 3168.98
3171.90 3179.31 3188.56 3196.56 3202.44 3203.06
3204.95 3216.65 3221.02 3238.69 3248.46 3266.74

==============================
syn-9
==============================
12.17 31.22 40.82 50.39 56.00 63.27 67.01 75.87 83.56
88.91 97.34 99.46 109.92 116.95 122.81 135.71 142.61
161.98 178.14 189.75 198.75 201.09 206.31 222.41 246.44
247.49 253.56 256.28 261.21 269.94 274.54 284.69 287.67
303.14 303.72 309.07 319.92 323.46 327.80 334.44 360.32
369.11 376.37 383.84 395.96 409.25 417.21 431.72 436.98
448.95 460.50 472.59 478.69 483.60 486.91 507.47 525.55
531.22 543.14 558.64 567.20 582.01 587.31 590.09 601.94
608.74 641.35 644.85 652.46 662.03 677.48 680.99 698.02
703.51 713.30 715.24 737.49 744.23 746.35 754.89 758.95
768.64 771.33 777.31 781.53 799.55 808.36 812.17 827.34
844.29 855.50 868.72 877.28 883.43 885.21 886.69 895.06
906.26 906.93 914.05 919.25 922.64 926.65 934.63 938.96
943.34 948.26 952.16 961.92 966.92 968.29 982.27 984.83
1001.55 1002.90 1012.64 1017.33 1023.97 1035.99 1039.99
1054.55 1064.76 1066.20 1072.78 1094.13 1097.57 1099.38
1112.74 1114.09 1124.43 1125.76 1126.28 1140.73 1154.36
1155.11 1161.15 1176.06 1176.69 1186.69 1192.09 1197.40
1206.92 1224.08 1227.26 1235.79 1236.57 1244.15 1250.03
1253.55 1260.27 1267.92 1268.20 1271.96 1274.18 1283.39
1287.23 1300.56 1303.00 1320.87 1321.79 1332.58 1335.89
1340.34 1341.75 1352.32 1368.28 1373.94 1381.93 1383.27
1383.53 1390.78 1406.03 1413.92 1425.64 1426.72 1433.54
1434.79 1474.16 1476.78 1486.91 1488.95 1490.98 1492.13
1494.72 1501.15 1506.09 1509.03 1509.99 1511.29 1514.18
1515.73 1516.66 1520.22 1523.39 1529.68 1539.51 1545.61
1563.42 1586.52 1615.08 1632.35 1649.20 1656.58 1665.56
1671.37 1684.61 1727.06 1733.41 1765.16 3019.11 3019.83
3023.53 3032.50 3037.57 3039.37 3044.36 3047.61 3065.08
3066.49 3067.02 3083.90 3093.96 3098.18 3107.04 3110.79
3110.84 3111.28 3116.31 3117.64 3121.72 3126.71 3130.74
3134.51 3148.10 3175.03 3182.77 3186.60 3194.74 3200.86
3204.51 3213.57 3219.92 3221.37 3248.90 3250.77 3299.74

==============================
syn-7m-TS
==============================
-339.48 22.41 26.29 35.99 37.29 42.09
50.13 52.37 54.79 67.84 78.94 91.90
100.99 111.89 125.99 129.88 132.72 147.97
158.84 163.62 170.24 189.36 198.95 215.67
224.59 226.33 248.86 253.39 254.94 257.52
258.83 270.39 272.08 278.89 289.35 299.47
303.56 305.31 319.11 328.46 335.88 338.83
366.01 373.04 378.89 390.23 397.24 414.66
432.12 432.93 440.15 446.21 446.68 451.05
461.14 470.81 472.49 485.81 496.23 515.12
528.21 542.88 545.26 562.26 571.69 583.80
588.82 592.90 604.85 614.35 626.71 642.67
659.65 661.63 672.99 689.86 692.36 716.43
722.45 725.96 736.88 743.05 743.58 752.88
758.91 765.68 772.64 778.02 789.39 795.69
815.50 824.12 834.86 846.25 852.48 859.68
864.16 872.21 876.09 887.17 888.56 894.31
903.06 906.04 910.63 925.02 933.24 934.82
940.00 942.51 943.38 949.07 962.15 973.89
984.05 986.37 987.77 1000.42 1001.55 1008.21
1011.44 1017.94 1023.38 1035.31 1058.44 1058.58
1068.53 1097.00 1106.59 1119.57 1124.88 1127.84
1138.63 1147.83 1155.23 1161.12 1173.25 1182.77
1183.43 1188.84 1189.16 1195.83 1201.27 1221.95
1229.11 1234.06 1238.07 1255.93 1260.20 1260.62
1268.84 1269.53 1272.87 1280.75 1290.49 1300.24
1308.11 1312.38 1320.16 1334.99 1336.74 1338.82
1341.43 1359.18 1366.80 1372.58 1380.70 1385.33
1388.51 1399.21 1411.34 1413.80 1424.98 1426.70
1434.19 1437.71 1449.42 1470.65 1481.18 1486.68
1489.69 1490.54 1494.48 1501.01 1505.72 1508.14
1510.45 1512.74 1513.82 1516.21 1517.18 1518.59
1519.17 1521.87 1527.25 1528.49 1547.08 1557.72
1570.21 1604.08 1613.24 1621.14 1632.58 1657.28
1667.41 1687.06 1698.77 1730.40 1736.75 3018.66
3022.03 3031.19 3037.19 3039.33 3043.90 3047.06
3052.18 3064.66 3087.92 3091.35 3097.46 3104.37
3107.08 3110.72 3111.72 3117.34 3121.66 3126.98
3147.87 3149.63 3163.57 3178.33 3189.15 3189.83
3196.76 3199.84 3203.63 3208.99 3216.44 3220.90
3222.69 3237.72 3239.55 3245.36 3262.41 3295.27

==============================
syn-7m-TS'
==============================
-510.63 21.40 28.12 29.91 41.17 41.76
55.89 62.25 73.56 78.66 94.36 98.19
99.93 107.53 114.07 127.80 136.79 145.04
156.69 167.16 174.13 191.31 195.83 202.79
223.47 230.18 245.67 253.00 255.40 263.82
270.09 273.95 282.42 287.87 293.77 303.00
304.93 318.00 323.35 325.03 327.81 335.48
353.67 368.28 382.46 383.94 399.76 407.16
430.28 434.30 440.94 444.46 449.09 461.21
468.56 471.59 475.40 480.68 497.89 513.19

528.76 543.01 550.82 555.56 575.57 583.50
588.16 606.56 618.55 622.60 647.88 658.97
672.90 681.47 695.39 699.81 719.34 725.89
728.08 743.42 748.60 750.81 752.37 759.02
760.23 769.06 772.20 779.13 797.10 799.48
815.43 827.92 835.23 835.40 858.82 861.08
866.42 869.81 885.48 886.97 888.47 891.23
896.22 908.97 924.10 928.70 931.90 936.14
937.72 944.17 950.86 953.93 964.92 977.05
979.41 986.74 997.68 1004.17 1008.85 1010.89
1015.32 1019.97 1023.72 1033.64 1058.39 1064.71
1065.40 1090.34 1099.43 1123.67 1126.07 1131.71
1136.36 1147.89 1158.52 1165.89 1171.06 1179.36
1188.12 1189.05 1191.66 1196.08 1206.11 1226.89
1231.10 1232.68 1236.08 1256.00 1256.94 1265.22
1269.99 1270.65 1273.81 1284.58 1288.76 1296.12
1308.21 1311.04 1320.52 1332.32 1332.91 1340.78
1342.93 1352.42 1367.36 1383.59 1384.50 1387.41
1391.70 1397.97 1412.49 1417.74 1425.19 1427.84
1434.02 1439.02 1465.91 1473.33 1484.10 1484.63
1487.74 1495.32 1501.64 1503.72 1505.97 1509.71
1511.29 1513.58 1514.67 1516.15 1516.53 1520.53
1520.93 1521.62 1522.35 1528.31 1543.73 1559.53
1561.05 1614.69 1617.83 1626.53 1635.16 1660.56
1668.26 1689.17 1691.26 1733.13 1734.49 3019.76
3024.60 3031.88 3032.90 3038.51 3040.13 3044.56
3048.74 3060.73 3069.34 3076.19 3092.65 3103.63
3105.25 3110.78 3111.23 3113.13 3120.48 3139.67
3142.23 3148.15 3154.27 3159.84 3178.84 3187.24
3188.58 3197.61 3203.45 3205.51 3210.08 3216.90
3220.50 3238.30 3242.99 3260.45 3264.96 3321.33

==============================

syn-8m

==============================

20.57 25.32 33.85 35.21 43.36 47.43
52.81 55.92 73.20 75.24 90.52 98.87
108.51 118.06 126.24 131.38 159.53 164.63
165.76 184.79 188.45 197.86 211.05 225.61
227.64 229.02 249.45 253.68 254.33 258.76
268.13 271.45 278.78 289.26 290.26 298.79
304.95 314.68 318.95 329.16 332.06 337.16
363.22 373.26 378.39 391.07 401.90 415.40
432.32 439.88 440.63 445.50 448.67 461.57
462.41 470.61 486.20 496.08 516.07 528.30
543.52 544.72 555.56 564.43 571.60 585.61
592.61 598.65 614.55 620.53 642.02 651.96
659.38 666.38 673.59 689.98 701.43 713.36
722.02 726.76 733.83 744.74 746.35 752.29
765.74 767.78 773.39 778.67 795.27 815.16
824.66 835.06 838.56 857.54 858.19 863.62
873.97 876.50 888.12 889.25 894.59 906.21
922.59 924.91 931.53 933.54 938.18 940.70
942.36 948.32 948.93 961.69 974.09 982.34
987.73 988.20 995.71 1001.79 1008.26 1012.19
1015.45 1023.56 1035.25 1057.68 1058.44 1068.03
1092.79 1098.13 1107.12 1118.62 1124.92 1127.61
1138.94 1147.76 1153.24 1155.02 1159.88 1161.67

1183.95 1187.38 1189.16 1189.80 1197.17 1228.70
1229.77 1231.88 1234.65 1238.56 1246.23 1255.41
1259.49 1269.69 1272.35 1290.56 1300.23 1307.85
1310.36 1312.87 1319.90 1327.53 1335.07 1338.08
1342.58 1366.85 1367.42 1380.75 1383.81 1384.28
1385.45 1387.07 1388.93 1410.82 1413.94 1424.92
1427.14 1434.55 1439.02 1469.67 1480.88 1482.25
1488.49 1491.01 1494.14 1498.30 1501.10 1505.63
1508.17 1510.17 1512.44 1513.85 1516.31 1518.62
1519.14 1519.52 1522.37 1529.26 1533.51 1558.60
1574.43 1611.54 1612.90 1632.46 1638.31 1657.52
1667.92 1687.76 1717.61 1734.76 1750.52 3019.38
3022.29 3031.25 3037.23 3039.67 3044.32 3045.54
3047.63 3054.28 3064.70 3094.75 3098.83 3107.20
3107.23 3110.67 3112.35 3112.43 3115.24 3121.00
3128.12 3134.15 3148.46 3158.51 3159.74 3179.40
3188.19 3190.04 3198.79 3200.82 3202.78 3209.27
3215.69 3219.75 3237.83 3244.93 3266.04 3302.92

==============================

os-syn-8m

==============================

21.59 22.03 26.65 35.06 43.96 49.31
52.57 53.35 67.84 77.43 92.86 99.41
102.79 116.97 125.01 131.41 134.04 159.68
164.52 179.60 187.77 197.78 213.43 225.09
227.03 229.31 244.85 249.73 253.85 254.49
258.88 270.88 278.69 288.84 290.25 294.28
298.84 305.09 318.94 329.20 331.65 337.08
363.46 373.19 378.40 391.06 398.14 415.46
432.22 439.89 441.42 445.35 449.30 460.40
461.52 470.54 486.21 496.05 514.28 516.09
528.27 543.50 553.35 567.34 571.81 585.55
592.50 598.34 614.58 621.63 641.81 647.52
659.20 672.15 678.81 690.07 692.00 714.17
722.02 726.62 732.96 744.87 746.37 752.29
765.16 766.03 773.42 778.60 795.29 814.99
824.67 835.06 839.86 856.95 858.22 864.62
874.18 876.52 888.16 889.11 894.59 906.19
924.54 926.38 929.37 933.58 936.79 940.49
942.35 948.84 949.52 961.69 975.01 983.13
987.73 989.42 997.01 1001.81 1008.25 1010.30
1012.06 1023.55 1035.19 1056.95 1058.45 1067.99
1086.19 1098.13 1106.99 1108.95 1118.44 1124.86
1127.60 1147.74 1152.48 1154.98 1159.45 1160.24
1182.44 1184.16 1189.05 1189.52 1197.21 1223.19
1229.31 1232.59 1234.70 1238.60 1245.37 1255.40
1259.44 1269.70 1272.33 1290.57 1300.34 1307.73
1310.05 1313.44 1319.82 1326.81 1335.02 1338.05
1342.62 1365.76 1366.84 1380.71 1380.78 1383.69
1385.45 1387.61 1389.30 1410.70 1413.88 1424.86
1427.12 1434.47 1437.84 1469.60 1480.79 1482.14
1488.14 1490.74 1494.07 1499.06 1501.24 1505.54
1508.06 1510.06 1512.38 1513.75 1516.19 1518.39
1518.54 1519.22 1522.07 1529.19 1532.51 1558.27
1573.44 1610.82 1612.88 1632.43 1637.54 1657.56
1667.97 1687.73 1717.55 1734.60 1749.80 3019.33
3022.28 3031.26 3037.21 3039.66 3044.32 3044.51

3047.65 3051.32 3064.68 3094.94 3098.96 3102.02
3107.22 3110.45 3110.67 3112.41 3115.08 3120.92
3128.17 3128.54 3136.41 3148.33 3154.87 3177.51
3187.13 3188.03 3195.58 3197.78 3202.70 3207.27
3215.76 3219.74 3237.63 3244.80 3245.48 3303.05

===============================
syn-9m
===============================
17.18 19.15 33.15 35.30 41.92 47.72
49.64 58.17 67.00 80.91 92.54 97.10
110.07 113.69 124.32 129.90 157.52 159.53
180.78 186.80 198.19 202.92 225.23 227.37
240.54 249.58 253.78 254.91 258.54 269.85
275.94 279.26 288.92 299.27 304.92 312.87
319.39 328.32 333.44 336.53 359.87 373.13
379.30 389.18 403.15 414.69 432.00 439.02
441.21 446.39 461.69 470.59 483.21 486.22
497.67 515.66 525.30 528.21 543.51 568.14
571.87 582.64 591.46 595.43 602.18 615.47
626.27 642.86 659.56 673.85 683.87 693.91
716.52 722.34 722.91 726.64 744.98 751.41
752.93 765.36 766.33 772.21 773.21 779.43
795.66 814.33 815.82 824.37 836.40 859.53
865.23 875.89 877.02 886.59 889.42 889.88
896.38 906.34 912.41 920.82 924.70 933.45
940.30 941.93 945.60 950.17 954.08 959.05
961.83 962.64 975.23 988.48 997.28 1001.78
1008.38 1011.17 1012.52 1023.43 1035.17 1036.73
1058.90 1066.73 1072.02 1078.03 1095.12 1097.19
1106.79 1113.35 1118.55 1124.68 1128.14 1141.68
1147.80 1155.22 1158.99 1160.51 1183.83 1184.95
1189.11 1196.03 1198.75 1214.06 1229.23 1234.75
1235.21 1236.46 1238.58 1253.69 1255.66 1259.53
1261.10 1269.99 1272.78 1273.62 1287.46 1290.70
1300.16 1308.44 1315.99 1320.07 1335.40 1337.78
1342.56 1344.06 1348.70 1366.48 1367.63 1381.07
1383.60 1384.24 1388.79 1411.09 1413.78 1424.13
1425.44 1427.45 1434.35 1472.22 1481.97 1489.27
1491.09 1494.37 1495.50 1501.19 1505.67 1506.84
1508.10 1510.23 1513.08 1514.01 1516.23 1518.63
1519.25 1521.99 1528.99 1530.84 1544.44 1561.53
1584.28 1613.45 1632.72 1648.93 1658.23 1667.52
1670.70 1688.43 1724.10 1736.50 1752.91 3018.89
3021.84 3031.26 3037.31 3038.84 3043.63 3044.36
3047.02 3058.41 3064.50 3069.57 3089.27 3093.07
3098.28 3107.49 3111.03 3112.04 3113.54 3115.46
3120.59 3125.88 3126.33 3128.48 3147.95 3170.75
3182.37 3189.32 3193.69 3194.24 3196.99 3204.00
3205.65 3216.60 3220.56 3238.82 3245.45 3287.25
